# Photocleavable *Ortho*-Nitrobenzyl-Protected DNA Architectures and Their Applications

**DOI:** 10.1021/acs.chemrev.3c00016

**Published:** 2023-04-20

**Authors:** Michael P. O’Hagan, Zhijuan Duan, Fujian Huang, Shay Laps, Jiantong Dong, Fan Xia, Itamar Willner

**Affiliations:** † Institute of Chemistry, The Hebrew University of Jerusalem, Jerusalem 91904, Israel; ‡ State Key Laboratory of Biogeology and Environmental Geology, Engineering Research Center of Nano-Geomaterials of Ministry of Education, Faculty of Materials Science and Chemistry, China University of Geosciences, Wuhan 430074, China

## Abstract

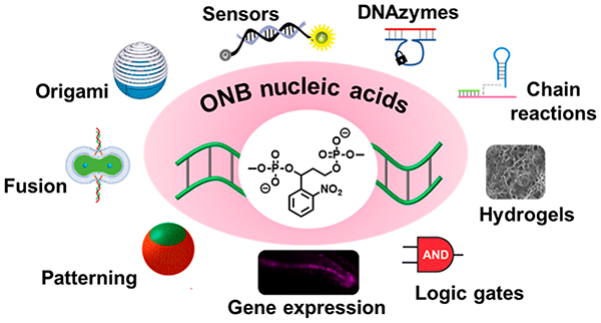

This review article introduces mechanistic aspects and applications of photochemically deprotected *ortho*-nitrobenzyl (ONB)-functionalized nucleic acids and their impact on diverse research fields including DNA nanotechnology and materials chemistry, biological chemistry, and systems chemistry. Specific topics addressed include the synthesis of the ONB-modified nucleic acids, the mechanisms involved in the photochemical deprotection of the ONB units, and the photophysical and chemical means to tune the irradiation wavelength required for the photodeprotection process. Principles to activate ONB-caged nanostructures, ONB-protected DNAzymes and aptamer frameworks are introduced. Specifically, the use of ONB-protected nucleic acids for the phototriggered spatiotemporal amplified sensing and imaging of intracellular mRNAs at the single-cell level are addressed, and control over transcription machineries, protein translation and spatiotemporal silencing of gene expression by ONB-deprotected nucleic acids are demonstrated. In addition, photodeprotection of ONB-modified nucleic acids finds important applications in controlling material properties and functions. These are introduced by the phototriggered fusion of ONB nucleic acid functionalized liposomes as models for cell–cell fusion, the light-stimulated fusion of ONB nucleic acid functionalized drug-loaded liposomes with cells for therapeutic applications, and the photolithographic patterning of ONB nucleic acid-modified interfaces. Particularly, the photolithographic control of the stiffness of membrane-like interfaces for the guided patterned growth of cells is realized. Moreover, ONB-functionalized microcapsules act as light-responsive carriers for the controlled release of drugs, and ONB-modified DNA origami frameworks act as mechanical devices or stimuli-responsive containments for the operation of DNA machineries such as the CRISPR-Cas9 system. The future challenges and potential applications of photoprotected DNA structures are discussed.

## 1 Introduction

**Figure 1:**
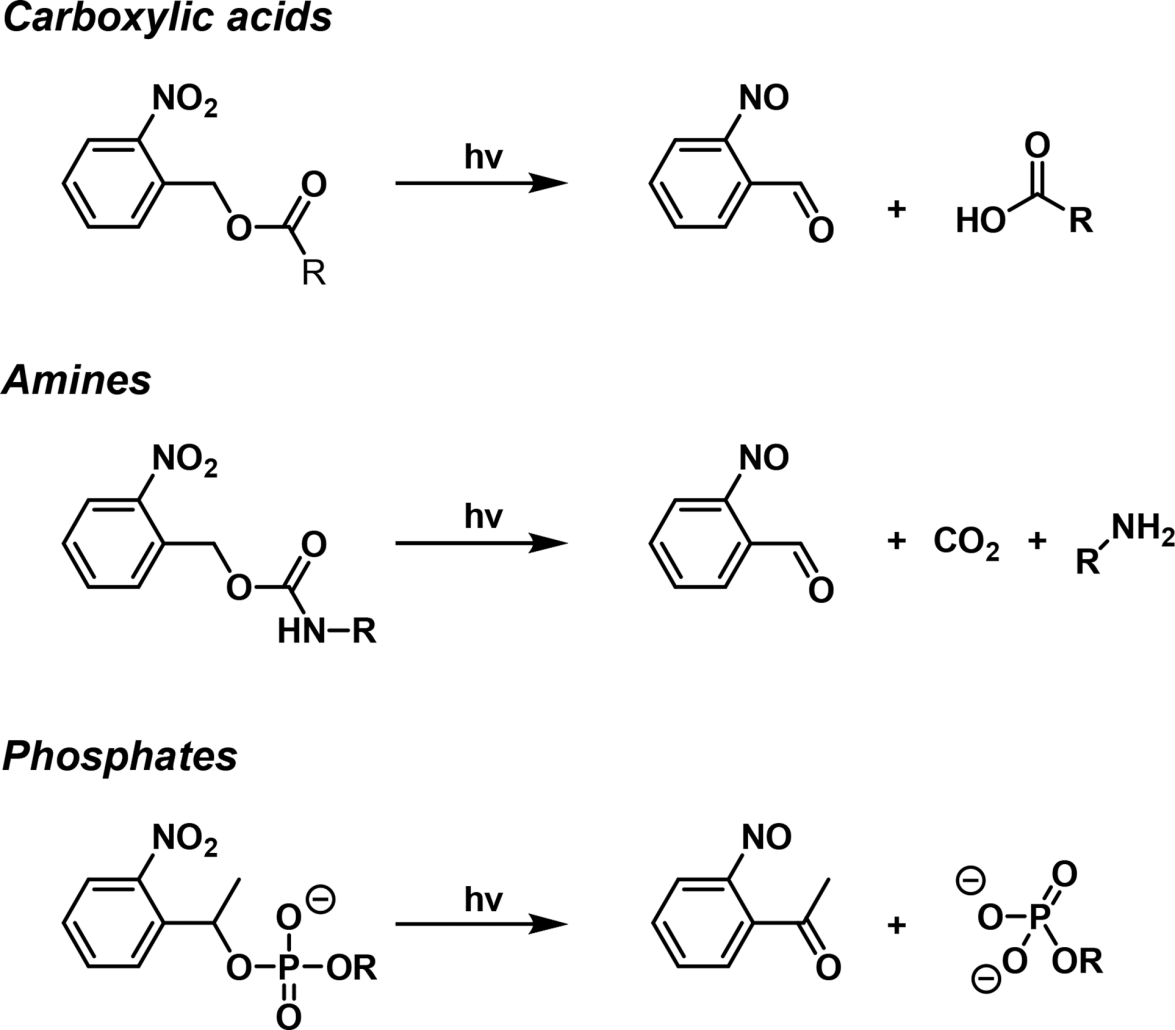
Examples of *ortho*-nitrobenzyl-protected chemical functionalities and light-induced cleavage to release the deprotected products.

Protecting groups for chemical functionalities play a key role in organic synthesis, particularly in the synthesis of biopolymers such as polypeptides,^1−6^ polysaccharides,^7,8^ and nucleic acids.^9,10^ The use of protecting groups involves the primary protection of a chemical functionality, the subsequent performance of the desired chemical transformation, and finally the deprotection of the protecting group to recover the parent chemical functionality.^11^ Diverse chemical means to protect and deprotect reactive functional groups were developed, and several monographs and review articles addressed this topic.^11−13^ An important approach in the development of protecting groups involves the application of photoresponsive moieties such as coumarins,^14,15^ phenacyls,^16,17^ benzoins,^18,19^ and arylsulfonyl esters^20,21^ that can be removed by light. The use of light as a deprotective means has significant advantages compared to chemical methods since it is often highly spatiotemporally controllable, selective to the photocleavable moiety and nonharmful to other chemical functionalities.^22^ A major class of photoresponsive protecting groups includes the *ortho*-nitrobenzyl (ONB) group^23^ that protects a range of functional groups including amines,^23,24^ carboxylic acids,^23,25^ phosphates^26^ and more. Figure 1 exemplifies several reaction schemes demonstrating the protection and photochemical deprotection of these chemical functionalities using this strategy (for further mechanistic aspects related to the photochemical deprotection, see Section 3.3).

For over half a century, numerous studies have applied the ONB photoresponsive protecting group to control synthetic transformations, such as in peptide synthesis,^27,28^ oligonucleotide synthesis,^29,30^ glycoside synthesis,^31,32^ and more. Moreover, the photoresponsive *ortho*-nitrobenzyl unit has been applied to develop a variety of technologies, including caged light-responsive ion carriers such as Ca^2+^ and Mg^2+^ ionophores^33^ and cryptands,^34^ photoresponsive polymer networks^35^ and photoresponsive hydrogels,^36,37^ photoactivated prodrugs and drug delivery systems,^38^ photocaged bioconjugates,^39^ such as insulin,^40^ for controlled release, aggregation of nanoparticles,^41^ and surface patterning.^42^ The advances in using the *ortho*-nitrobenzyl protective group in chemical synthesis and diverse applications were summarized in several comprehensive review articles.^35,37,38,43,44^

The base sequence comprising oligonucleotide biopolymers encodes substantial functional and structural information into these biopolymers. Besides controlling the stability of duplex nucleic acids by the number and chemical nature of bases comprising the biopolymer,^45,46^ the base composition of oligonucleotides dictates structural features of DNA, particularly the dynamically triggered reconfiguration of the polymer secondary structure. This includes, for example, the fuel/antifuel displacement of nucleic acid duplexes,^47,48^ the pH-stimulated formation and dissociation of cytosine rich strands into i-motif structures,^49−52^ the reversible pH-induced formation of C-G•C^+^ or T-A•T triplex assemblies,^53−56^ the K^+^-ion or Pb^2+^-ion stimulated formation of guanosine-rich G-quadruplex systems and their separation by crown ethers and cryptands,^57−60^ and the metal ion bridging of mismatched bases in duplex nucleic acids, e.g. the formation of T-Hg^2+^-T or C-Ag^+^-C bridges, and their separation by ligands such as cysteine.^61−67^ In addition to the base-guided structural information encoded in the base sequence of oligonucleotides, base-dictated functions are also embedded into the biopolymer. Sequence-specific recognition properties (aptamers)^68−74^ of low-molecular weight substrates (e.g., cocaine^75^ or ATP^76^) or biomacromolecules (e.g., thrombin^77^ or VEGF^78^) and the sequence-dictated catalytic properties of nucleic acids (DNAzymes),^79−81^ such as the hemin/G-quadruplex peroxidase-mimicking DNAzyme^82^ or metal-ion cofactor DNAzymes,^83^ were demonstrated. Also, sequence-guided reactions with enzymes, such as specific cleavage by nicking enzymes,^84^ endonucleases,^85^ or polymerases, were reported.^86,87^ These unique features of oligonucleotides provide a rich tool-box that paves the way to the development of the topic of DNA nanotechnology.^88−90^ The reconfigurable properties of oligonucleotides were broadly used to develop DNA-based switches and machines,^91−95^ such as tweezers,^67,96^ walkers,^97,98^ interlocked catenanes,^99^ or transporters.^100^ In addition, the triggered reconfiguration of nucleic acid strands provides versatile means to guide the formation of DNA nanostructures, to control the aggregation/disaggregation of DNA nanoparticles^50,101^ or to stimulate the oligomerization of DNA scaffolds such as the switchable dimerization/trimerization of origami tiles^102,103^ or DNA tetrahedra.^104^ Diverse applications of reconfigurable DNA structures were suggested, including the development of sensors,^105−109^ gated drug carriers for controlled release,^110−117^ and stimuli-responsive DNA-based materials such as hydrogels revealing controlled stiffness properties,^118−120^ shape memory,^121−123^ self-healing,^124,125^ and mechanical applications.^126,127^ In addition, the triggered reconfiguration of DNA nanostructures and the control over the optical properties of the systems found extensive applications in intracellular sensing,^128^ imaging,^129−131^ and therapeutic applications.^132,133^

The interaction of light and nucleic acid structures has attracted specific interest. In contrast to the interaction of auxiliary triggers such as pH, chemical agents, enzymes or oligonucleotide fuel/antifuel strands to manipulate DNA structures, all of which alter the composition of the systems by generating waste products, light provides a clean energy source to control the structures and properties of oligonucleotides.^134−136^ One approach to couple light to DNA structures involves the binding of π-conjugated chromophores as intercalators or groove binders into duplex DNA^137−140^ or the affinity complexation of metal–organic complexes, such as Ru(II)-polypyridine complexes, to minor/major groove domains of duplex DNA structures or as intercalators.^141−144^ Photoinduced electron transport across duplex DNA scaffolds and the probing of the conductivity features along the structures,^145,146^ and photoinduced electron transfer accompanied by DNA cleavage^147,148^ attracted substantial research efforts. In addition, the discovery that photoisomerizable molecular organic agents such as *trans*/*cis* azobenzene compounds exhibit light-controlled binding intercalation affinities toward duplex DNA structures played a key role in the development of the area of DNA nanotechnology.^149^ The effective intercalation of *trans*-azobenzene constituents into the double-stranded oligonucleotides and the accompanying stabilization of duplex DNA, while the lack of binding affinities of *cis*-azobenzene constituents to duplex nucleic acid structures led to versatile means to reversibly reconfigure duplex DNA nanostructures.^134^ Indeed, many different light-induced DNA-based switches and machines relying on the reversible *trans*/*cis* azobenzene reconfiguration of DNA structures were developed.^150−152^ Also, the light-controlled oligomerization of DNA nanostructures, such as origami frames,^153^ or the switchable control over material properties by means of photoresponsive DNA-functionalized constituents, such as controlled stiffness of azobenzene-functionalized hydrogel matrices, were reported.^154−156^ Indeed, the light-induced switchable stiffness properties of azobenzene-functionalized hydrogels were broadly applied to develop shape memory, self-healing, and controlled drug release matrices.^157,158^ In addition, azobenzene-modified DNA networks demonstrated dynamic light-induced reconfiguration functionalities^159^ and coupled control over dynamic catalytic transformations.^160^

**Figure 2:**
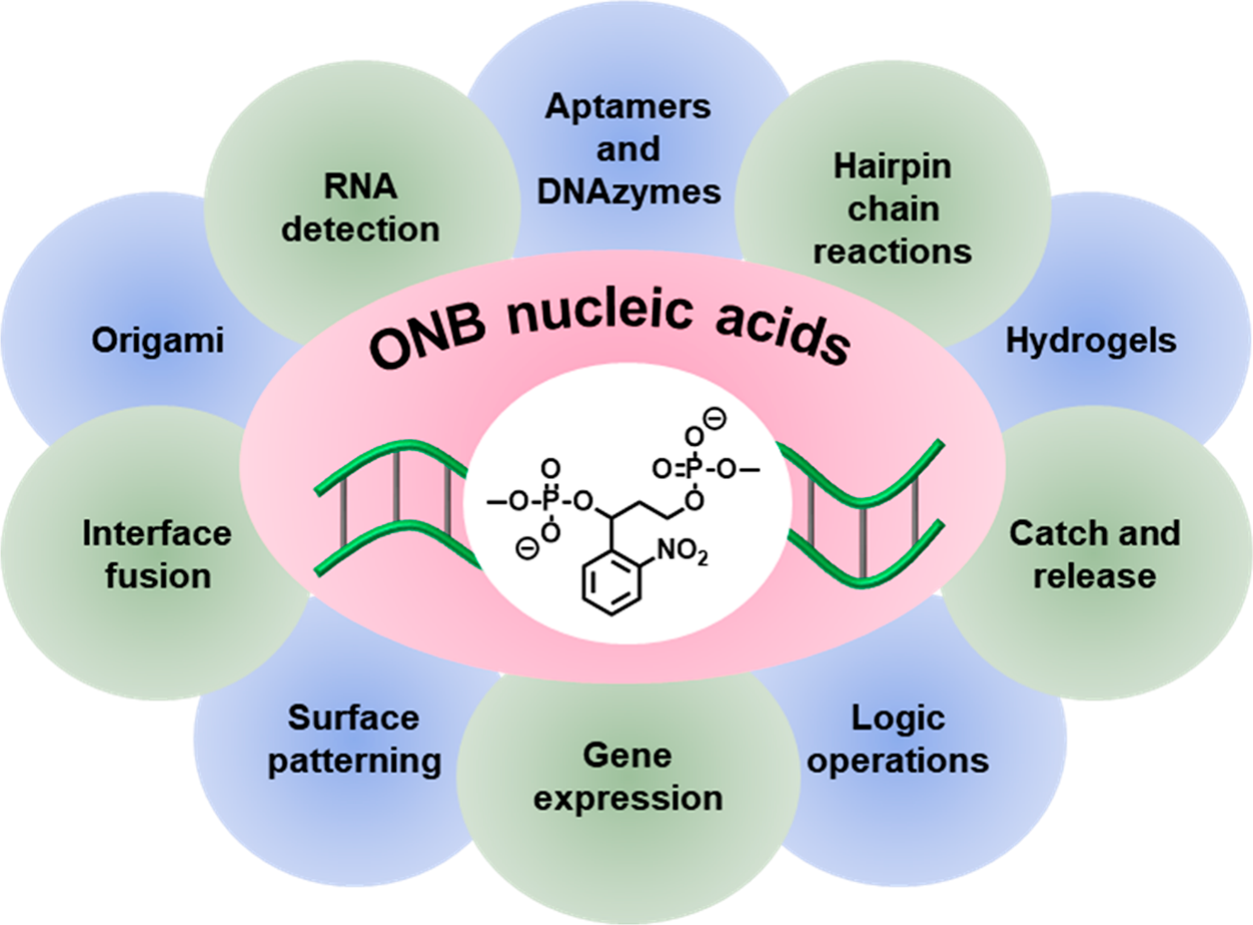
Diverse applications of *o*-nitrobenzyl (ONB)-protected nucleic acids in DNA nanotechnology and DNA-based materials.

Beyond the use of reversible photoisomerizable constituents as functional units to reconfigure nucleic acid nanostructures, the single-cycle photochemical uncaging of ONB-protected nucleic acids finds broad applications in the photoactivation of nucleic acid nanostructures (Figure 2).^161−164^ The present review article addresses the synthetic principles to tailor ONB-protected nucleic acids and the photochemical principles to uncage the protected nucleic acids. Diverse applications of photoresponsive ONB-protected nucleic acids in the developing areas of DNA nanotechnology and DNA-based materials are discussed. The photodeprotection of DNA frameworks, using ONB-protected nucleic acids, introduces new dimensions into the field of DNA nanotechnology as it enables the spatiotemporal activation of DNA structures and functions. The localized triggering of nucleic acid strands is important, for example, for activation of DNA in confined cellular environments or programmed surface domains.

While the concept of ONB-functionalized photocleavable DNA nanostructures has been addressed in several review articles on photoresponsive biomolecules,^161−167^ there is, as of yet, no review that exhaustively examines the full diversity of principles and applications of ONB-functionalized photocleavable DNA architectures across the whole breadth of biology, nanotechnology and materials science. Our aim in the present review is to provide a comprehensive overview on the use of ONB-functionalized photocleavable DNA structures for diverse applications within all these areas, especially into directions that paved recent new developments. Furthermore, we note that other photocleavable protecting groups, such as *p*-hydroxyphenacyls,^168^ thioether-enol phosphates,^169^ aryl sulfides,^170^ benzophenones,^171^ and coumarins,^172^ were reported as caging groups for oligonucleotides. Nevertheless, their application in the presence of nucleic acids is limited due to cross-reactivities with the nucleotides or phosphate frameworks. The versatile synthetic pathways to integrate the ONB units into DNA scaffolds represent major advantages for use as photoprotective DNA structures (for further discussion, see Section 3).

## 2 Principles of Engineering Photocleavable DNA Nanostructures

### 2.1 Photoactivated Toehold-Mediated Strand Displacement

**Figure 3:**
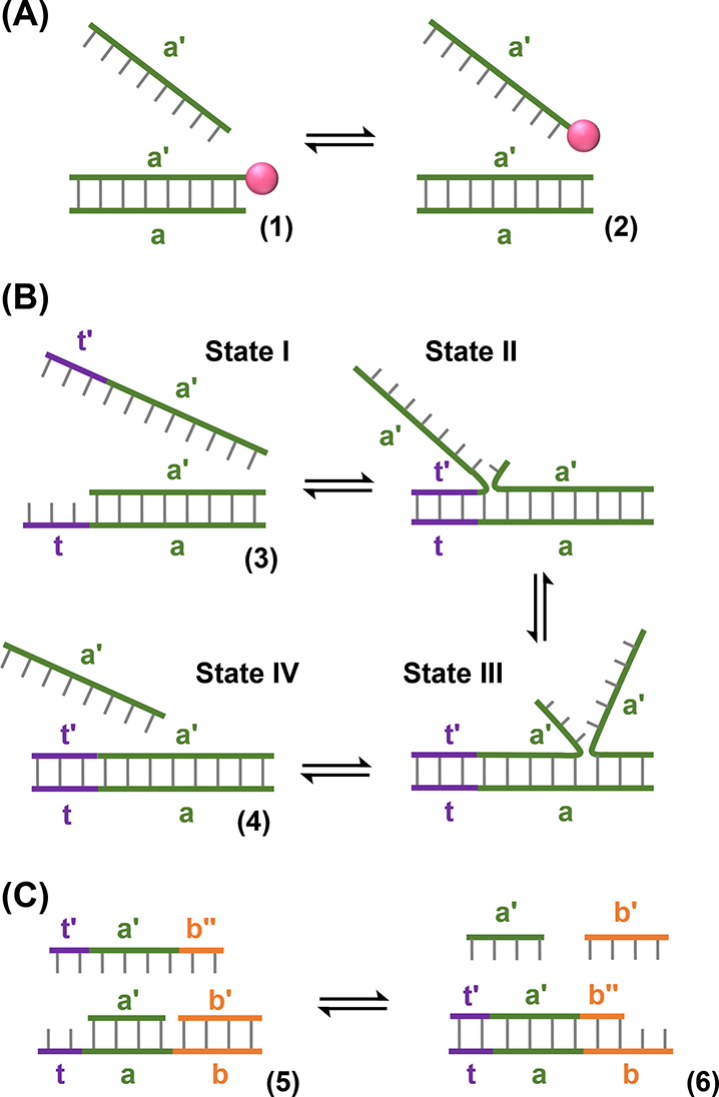
Displacement pathways of duplex nucleic acid structures. (A) Isoenergetic displacement of a duplex by a fuel strand. (B) Displacement of a toehold-functionalized duplex by a fuel strand. (C) Entropy-driven displacement of a duplex structure by a fuel strand.

The predictable and programmable nature of DNA duplex formation, based on the number and chemical nature of the bases present in the constituent nucleotides, allows for the design of intricate DNA reaction networks based on strand displacement phenomena, in which the addition of an invading oligonucleotide strand stimulates the displacement of a constituent strand of a duplex.^45,46^ In turn, the released strand may trigger further hybridization or displacement events, and the constituent strands may be functionalized, for example, with fluorophore/quencher pairs^173−175^ or enzymes,^176,177^ to generate spectroscopic or functional outputs. The factors affecting the kinetics and thermodynamics of strand displacement reactions are well studied, and a summary of key concepts was included in a recent review.^46^ The simplest type of duplex strand displacement is shown in Figure 3A. A duplex (**1**) consists of two strands, a and a′, the latter of which is tagged by a labeling moiety. Upon addition of unlabeled a′ as the invader strand, the dissociation of the labeled a′ strand from duplex (**1**) followed by hybridization of the unlabeled a′ strand results in the formation of duplex (**2**) while the labeled a′ is released as a free strand. This process is, however, kinetically inefficient due to the stability of the initial duplex (dissociation rate ∼ 1 × 10^–2^ s^–1^ for 10 base pairs).^178^ Indeed, at temperatures below the melting temperature, the strand displacement process is only initiated upon spontaneous end-fraying of the initial duplex, which provides an anchoring site for three-way branch migration to begin.^179^ Thus, for applications in DNA nanotechnology, systems of the type depicted in Figure 3A are of limited use.

The efficiency of strand displacement reactions may be substantially improved, however, by the addition of a single-stranded domain, known as a toehold, to the duplex structure and engineering the invader strand to contain the complementary sequence to this toehold.^47^Figure 3B depicts this principle. Duplex (**3**) bears a single-stranded toehold domain, t, adjacent to the hybridized sequence a. The invader strand contains the complementary sequence, t′, in addition to domain a′. Upon adding the invader strand to duplex (**3**), State I, hybridization of the free toehold regions (t/t′) anchors the invading strand to the initial duplex, State II, and therefore significantly increases the probability that branch migration will proceed, State III. Following full hybridization of the invader strand to form duplex (**4**) the shorter strand (containing domain a′ only) is released, State IV. The equilibrium is driven in the forward direction by the enhanced thermodynamic stability of the longer duplex (resulting from the contribution to the free energy of the hybridization of the t/t′ domains) and, since the product duplex (**4**) no longer contains a free toehold for binding of the displaced a′ strand, the reverse reaction is kinetically inhibited (*vide supra*). The kinetic efficiency of the forward process is governed by the length of the toehold. For toeholds of 1–5 bases in length, the effective rate of the forward strand displacement process was found to increase with increasing toehold length, while no further rate enhancement is achieved when toeholds of greater than five bases are employed.^47,180,181^ In addition to the difference in thermal stability of the duplexes, strand displacement reactions may also be driven by an increase in configurational entropy of the system, Figure 3C.^48^ In this case, while the duplex structures (**5**) and (**6**) contain the same number of base pairs, the number of free constituents increases in the forward direction of the reaction. This increase in entropy accompanying the strand displacement process is sufficient to drive the reaction in the forward direction even in the absence of additional complementary base pair stabilization.

**Figure 4:**
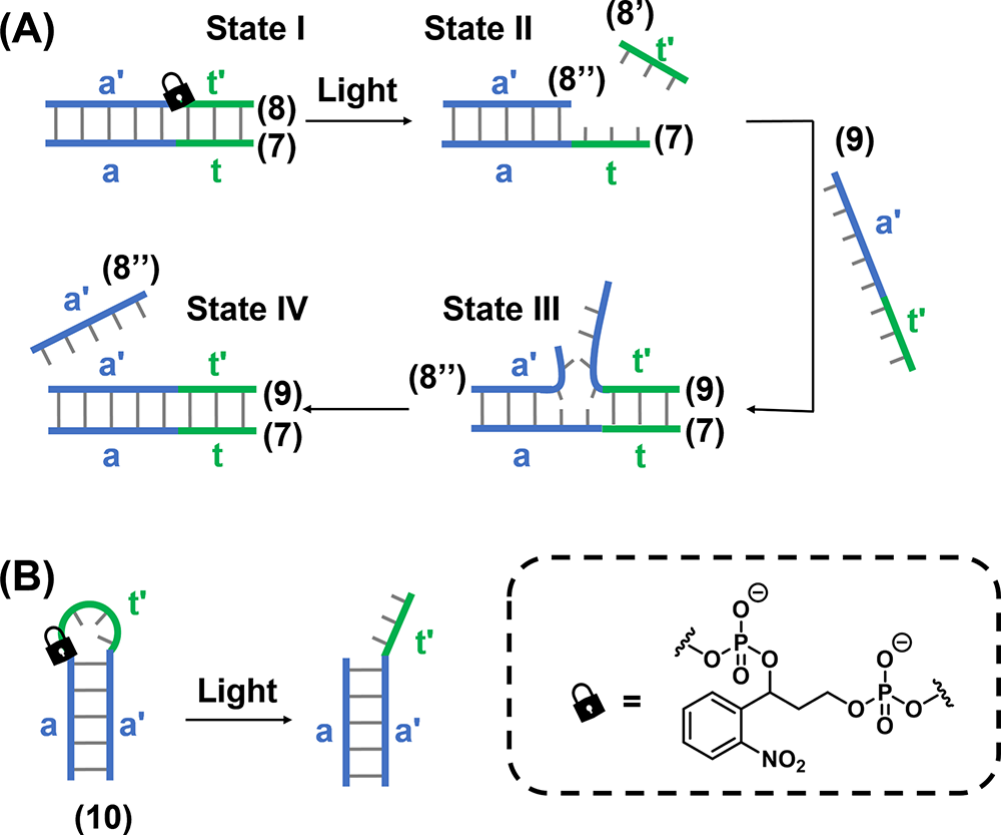
(A) Photoinduced displacement of an ONB-locked duplex DNA in the presence of a fuel strand by the photochemical deprotection of the parent duplex and the toehold-mediated displacement of the cleaved product by the fuel strand. (B) Photoinduced cleavage of an ONB-protected hairpin structure containing a masked toehold in the loop region. Upon activating of the toehold, displacement by a fuel strand can proceed in the same manner as (A).

Toehold-mediated strand displacement reactions of the types depicted in Figure 3 have been exploited in a variety of applications including the development of DNA nanotechnologies such as DNA walkers,^182−184^ the DNA-guided organization of nanoparticles,^176,185−188^ control of properties of DNA-based hydrogels,^189−191^ and the reversible opening and closing of DNA-based containments for the controlled release or display of loads.^192,193^ In addition, a range of applications in sensing technologies^194−196^ and synthetic biology^197,198^ have been demonstrated. However, a limitation of the toehold-mediated strand displacement reactions discussed above is that the single-stranded toehold regions are permanently available for hybridization with invading strands. Thus, achieving the precise spatiotemporal control over their activity is challenging and a barrier to certain applications.^166,199^ For example, in devices engineered to perturb gene expression, it is desirable to cage the reactivity of the system and then activate the sensing capability in a specific cell, or even at a particular phase of the cell cycle. The possibility to control DNA structures with light, through incorporation of photoresponsive ONB moieties, provides an elegant means to overcome these difficulties, by allowing the protection of the toeholds within duplex structures and their subsequent unmasking by photoirradiation. The principle of photoactivated toehold-mediated strand displacement based on ONB-modified DNA duplexes is depicted in Figure 4A.^200^ Self-complementary duplex (**7**)/(**8**) is engineered to contain a photocleavable ONB linker in strand (**8**), State I. Upon ONB photocleavage, strand (**8**) breaks into two shorter stands (**8′**) and (**8″**). While strand (**8″**) retains the base composition that stabilizes the hybrid (**8″**)/(**7**), the (**8′**) strand is too short to form a stable duplex with (**7**). Thus, strand (**8′**) dissociates from strand (**7**) exposing a single stranded toehold (t) in strand (**7**), State II. This region is engineered to hybridize with the complementary domain (t′) on invading strand (**9**), State III, which, in turn, triggers the strand displacement of (**8″**) from (**7**) driven by the eventual formation of the more stable (**7**)/(**9**) duplex, State IV. Thus, in the rest state, the (**7**)/(**8**) duplex is stable and inert to the presence of the invader (**9**) strand, while photoirradiation uncages the reactivity of the duplex, allowing the spatiotemporal control of the subsequent stand displacement reaction. The toehold may be, also, masked in the loop region of a self-complementary DNA hairpin, Figure 4B.^201^ In the intact state the stability of the hairpin duplex (**10**) stem prevents access to the masked toehold, while the phototriggered strand breakage event acts to open the hairpin loop and renders the toehold accessible for hybridization with a complementary target.

**Figure 5:**
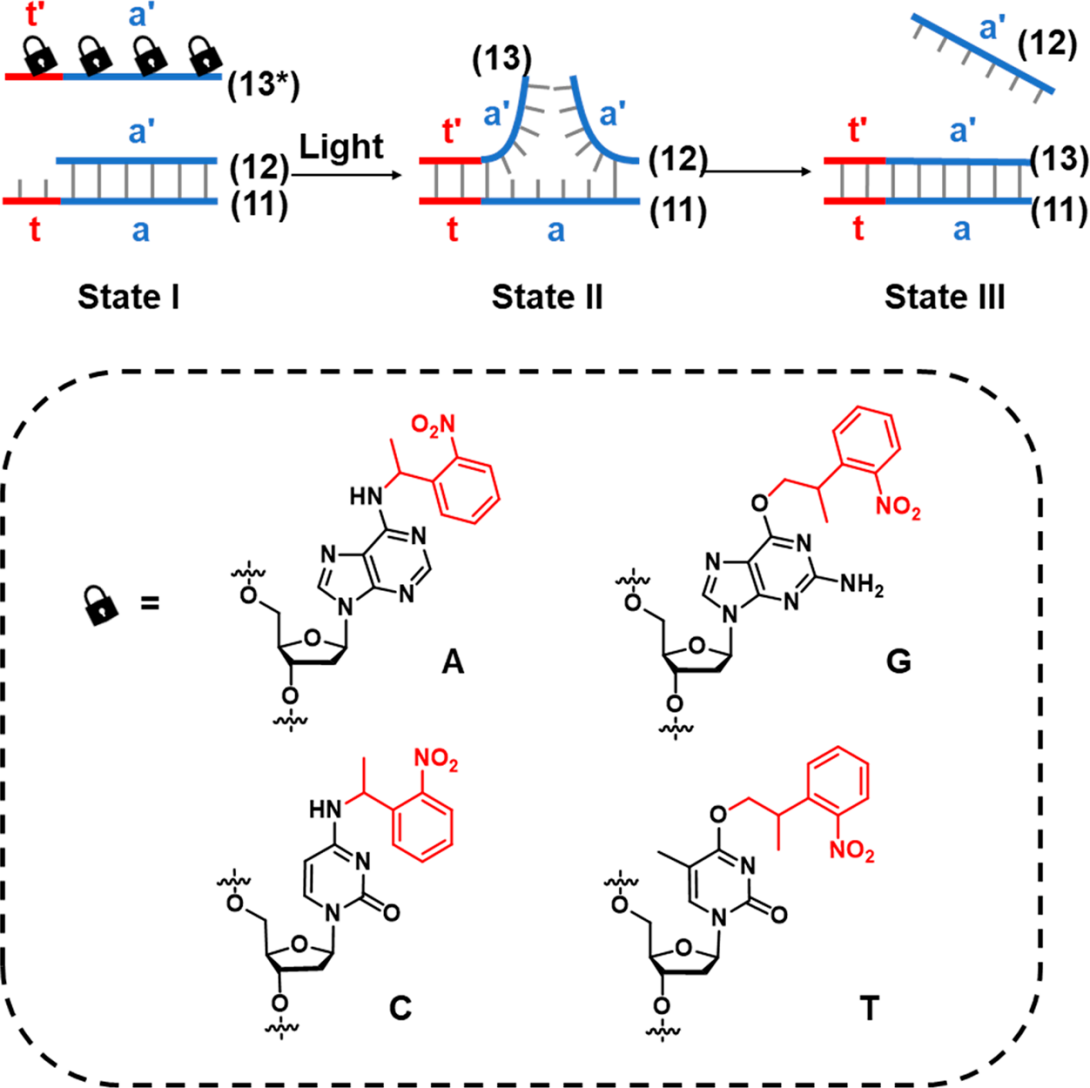
Schematic application of ONB-caged oligonucleotide bases for the light-induced toehold-mediated separation of a duplex nucleic acid. Photoprotected fuel strands are prohibited from initiating the toehold-mediated displacement process. Photodeprotection of the fuel strand releases the active fuel strand for the toehold-mediated displacement of the duplex structure.

An alternative means to photocage the reactivity of DNA strands toward toehold-mediated strand displacement is to functionalize strategic positions of the DNA nitrogenous bases with photocleavable protecting groups.^135,161^ By blocking the key hydrogen bonding sites within the nucleobase, the formation of Watson–Crick duplex hybrids with complementary bases is prevented, thus caging the reactivity of single-stranded DNA toward strand hybridization. Upon photocleavage of the protecting group from the protected bases, the hydrogen bonding functionality is unmasked, activating the strand toward duplex formation. The principle of employing these photoprotected nucleobases in the design of photoactivatable toehold-mediated strand displacement is shown in Figure 5.^202^ A self-complementary duplex (**11**)/(**12**) is engineered to contain a single-stranded toehold (t) in strand (**11**). The caged invading strand (13*) contains the complementary sequence to this toehold. However, the toehold-mediated strand displacement of (**12**) by (13*) is prevented by the installation of the photocleavable protecting groups on strategic bases in the (13*) strand, preventing the formation of the (**11**)/(13*) duplex hybrid, State I. Upon photoirradation, the protecting groups are cleaved to generate the uncaged strand (**13**), which subsequently displaces strand (**12**) following recognition of the toehold domain (State II) to form the energetically stabilized (**11**)/(**13**) duplex (State III).

### 2.2 Phototriggered Release of Caged Strands

**Figure 6:**
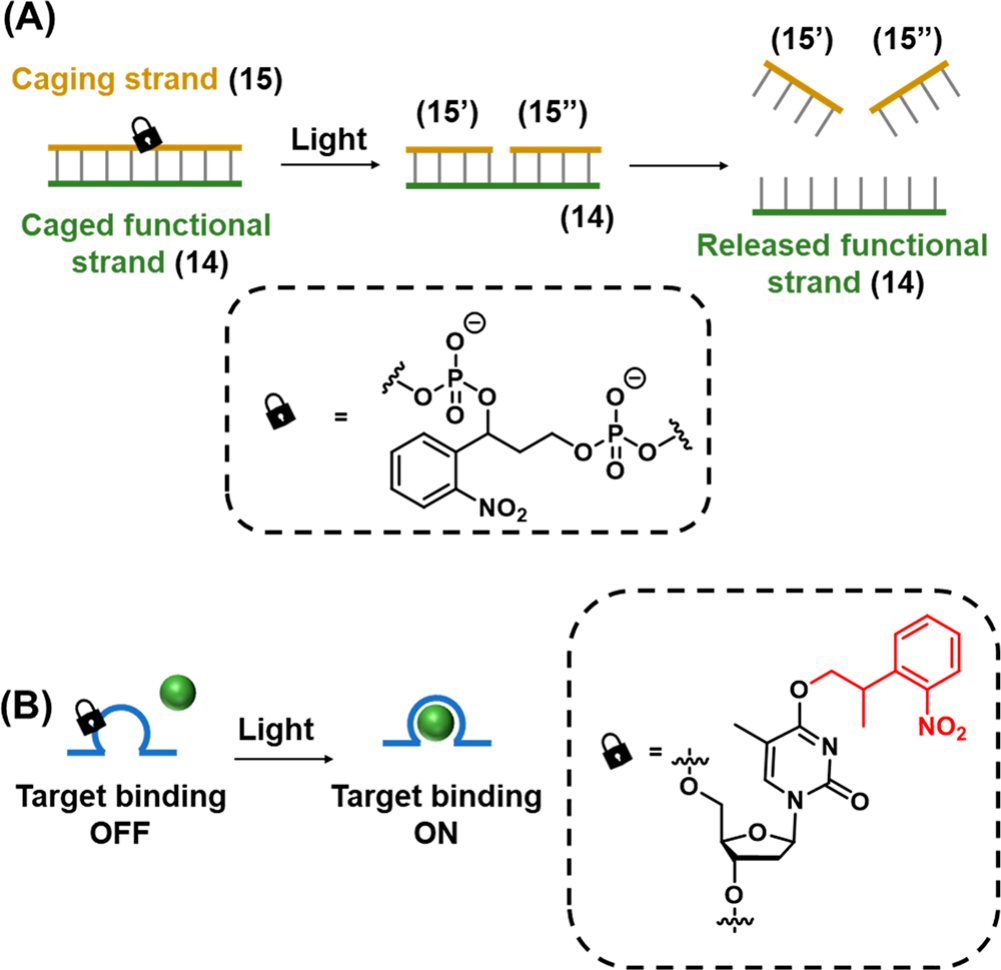
Functional photoinduced reconfiguration of nucleic acid structures caged by an ONB protective group. (A) An ONB-caged duplex structure being cleaved by light into two subunits being separated from the duplex structure due to insufficient base stabilization of the resulting fragments. (B) Light-induced uncaging of an ONB-caged sequence in which target binding affinity is perturbed by the presence of the caging moiety. Cleavage of the ONB from the caged base activates binding of the DNA strand to the target.

Beyond the photoactivation of toeholds, a complementary strategy for engineering photoresponsive DNA structures is depicted in Figure 6A.^199,203^ The functional strand (**14**) is caged by formation of a stable duplex to a complementary caging strand (**15**), engineered to include a photocleavable ONB moiety in the phosphate backbone. In the intact state, the melting temperature of the caging and functional strand is sufficiently high to retain the duplex in a stable configuration at the operating temperature of the system. Cleavage of the photolabile moiety leads to breakage of the caging strand (**15**) into two shorter fragments (**15′** and **15″**), each with significantly lower melting temperatures to the functional strand. Dissociation of these fragments releases the functional strand (**14**). Thus, the target strand is sequestered by duplex formation prior to photoirradiation and released upon triggered photoirradiation. The function of a DNA strand may also be caged by strategic incorporation of protecting groups on specific bases at key locations critical for binding of the strand to a target molecule such as a protein (in the case of aptamers)^204^ or a metal ion cofactor (in the case of DNAzymes)^205^ by blocking the binding site either sterically or by masking key hydrogen bonding functionality in the base that is critical for target recognition, Figure 6B.

## 3 *O*-Nitrobenzyl (ONB)-Modified Nucleic Acids: Synthesis and Photocleavage

### 3.1 Synthesis and Deprotection of ONB-Modified Nucleic Acids

**Scheme 1:**
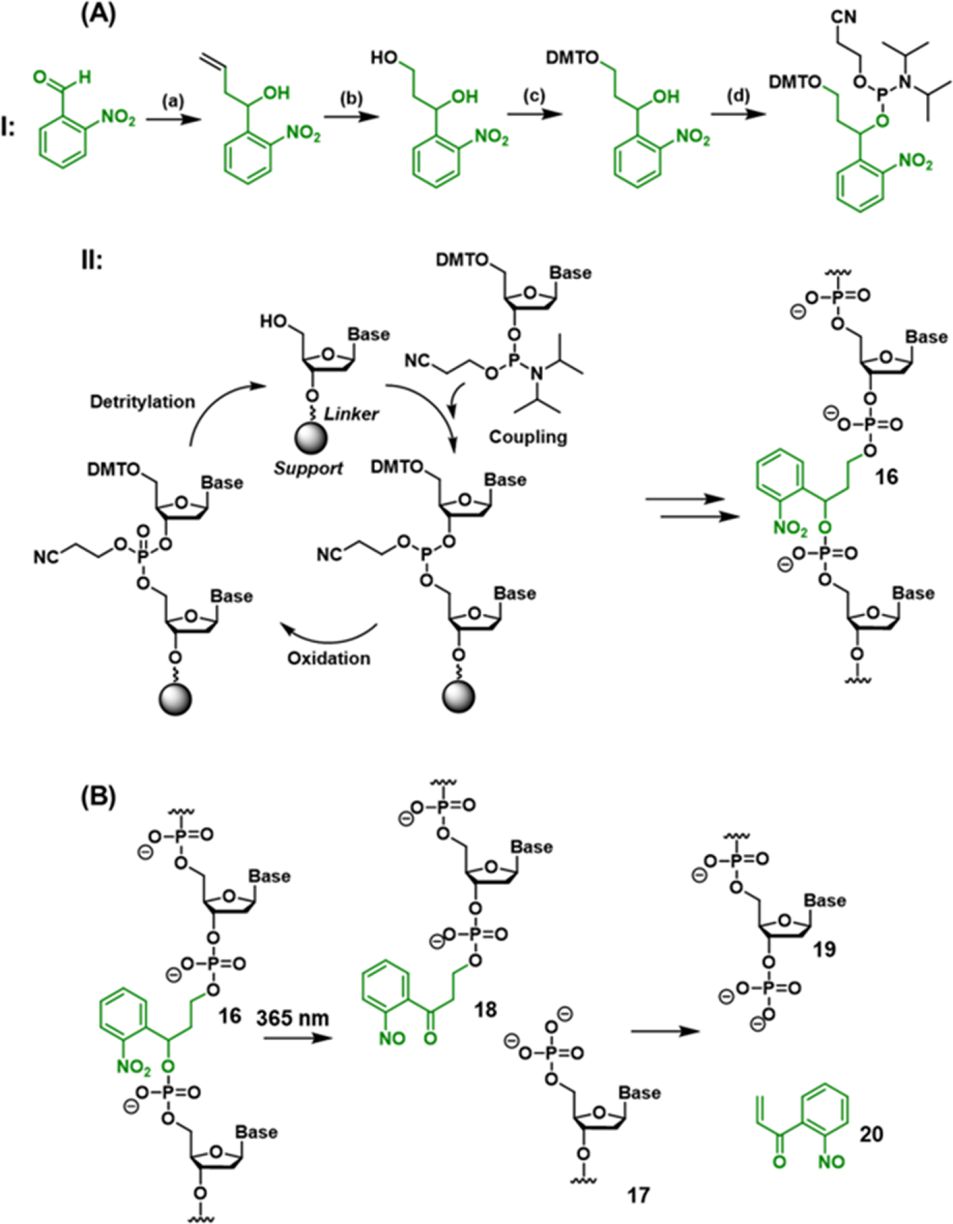
Synthesis and Photocleavage of an ONB-Protected Nucleic Acid Containing the ONB in the DNA Backbone (A) Panel I: Synthesis of the ONB-functionalized phosphoramite linker for incorporation into the oligonucleotide backbone by solid-phase synthesis. Synthetic pathway: (a) allylation, (b) ozonolysis/reduction, (c) DMT protection, (d) formation of phosphoramidite. For full synthetic details, see reference (206). Panel II: Solid-phase synthesis methodology for oligonucleotide synthesis. Each cycle incorporates an additional phosphoramidite-activated nucleotide (or linker) into the oligonucleotide chain. (B) Light-induced photocleavage of the ONB-functionalized strand.

**Scheme 2:**
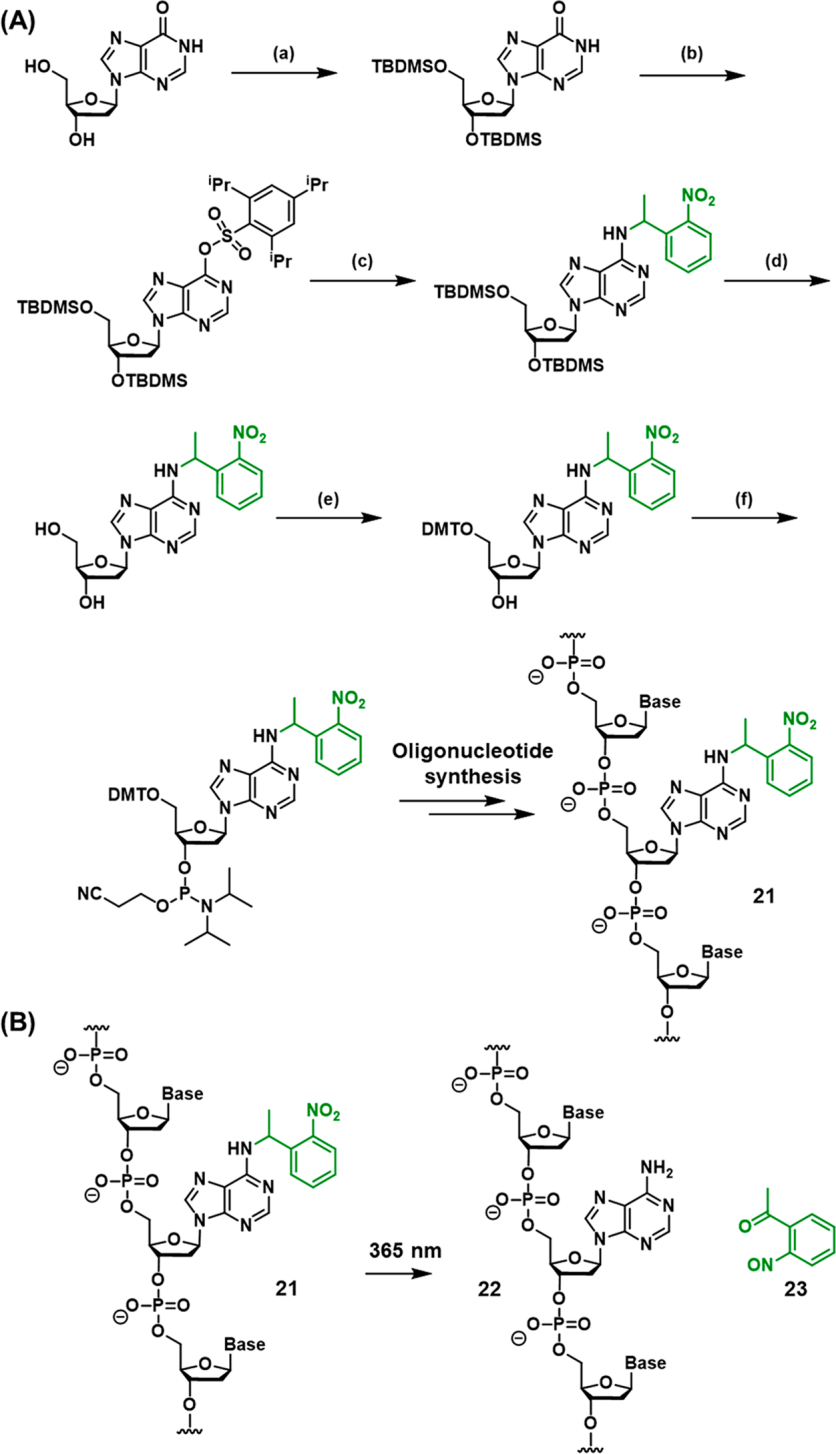
(A) Synthetic Pathway Generating an ONB-Protected Nucleic Acid Containing a Caged Adenine Base; (B) Light-Induced Deprotection of the ONB-Protected Oligonucleotide Base Synthetic pathway: (a) 3′/5′-OH TBDMS protection, (b) O6 activation, (c) C6 substitution, (d) 3′/5′ OH deprotection, (e) 5′-OH DMT protection, (f) formation of phosphoramidite. For full synthetic details, see reference (207).

The synthesis of ONB-modified DNA structures for use in DNA nanotechnology is based on the use of ONB-functionalized phosphoramidite monomer building blocks in solid-phase oligonucleotide synthesis. Incorporation of an ONB-containing linker unit into the DNA phosphate backbone allows the photogeneration of strand breaks (Scheme 1),^206^ while installation of the ONB-protecting groups onto DNA bases gates the reactivity of the DNA toward strand hybridization (Scheme 2).^207^

Scheme 1A depicts the stepwise synthesis of the photocleavable ONB-functionalized phosphate ester into the oligonucleotide chain (**16**) (the synthetic steps to synthesize the photoprotected phosphoramidite are presented in Scheme 1A, Panel I, and the subsequent solid-phase synthetic steps employed to form the oligonucleotide (**16**) are summarized in Scheme 1A, Panel II). For further details on the principles of solid-phase oligonucleotide synthesis, the reader is directed to several recent review articles on the topic.^208−211^

Upon irradiating the synthesized oligonucleotide with UV light, photocleavage of the ONB moiety proceeds, releasing strand **17** (containing free 5′ phosphate), Scheme 1B. Subsequent elimination from nitrosophenone **18** yields the 3′ phosphorylated strand **19** and unsaturated nitrosoketone **20** as the final byproduct.^206^

The incorporation of an ONB-protected nucleobase into the oligonucleotide framework is exemplified in Scheme 2A with the synthesis of an ONB-protected adenine nucleotide to form the ONB-functionalized oligonucleotide **21** (the synthetic steps involved in the synthesis of the protected nucleotide are summarized in the caption of Scheme 2). Incorporation of the protected base into the oligonucleotide by solid-phase synthesis (see Scheme 1A, Panel II) affords protected strand **21.** As before, irradiation of the strand with UV light triggers the cleavage of the protecting group to release the deprotected base (strand **22**) and the nitrosoketone (**23**) as a side product (Scheme 2B). Synthetic methodologies to prepare ONB-protected cytidine,^167^ thymidine^212^ and guanosine^213^ bases are also reported.

### 3.2 Wavelength Considerations

Of prime importance when attempting to design photoresponsive systems for functional applications is the need to ensure that the wavelength required to trigger the photochemical process is compatible with the application of interest. This is particularly critical when attempting to install photoresponsive functionality into DNA-based systems, since DNA itself absorbs UV light at wavelengths lower than 320 nm,^214^ which leads to a variety of excited state photochemistry that can either damage the carefully constructed DNA nanosystems or, in biological contexts, lead to deleterious effects and toxicity to living cells.^215^ Moreover, many enticing *in vivo* applications of DNA nanotechnology (such as biomarker detection or imaging) require specific photoactivation of the system inside biological tissue. Thus, long wavelengths of light (red and near-IR) are required for activation, since shorter blue- and UV-wavelength light is unable to penetrate effectively through biological matter.^216^ Unfortunately, the parent ONB system requires relatively short wavelength (λ = 365 nm) UV light for photodeprotection, and thus strategies to achieve longer wavelength activation are actively pursued.

**Figure 7:**
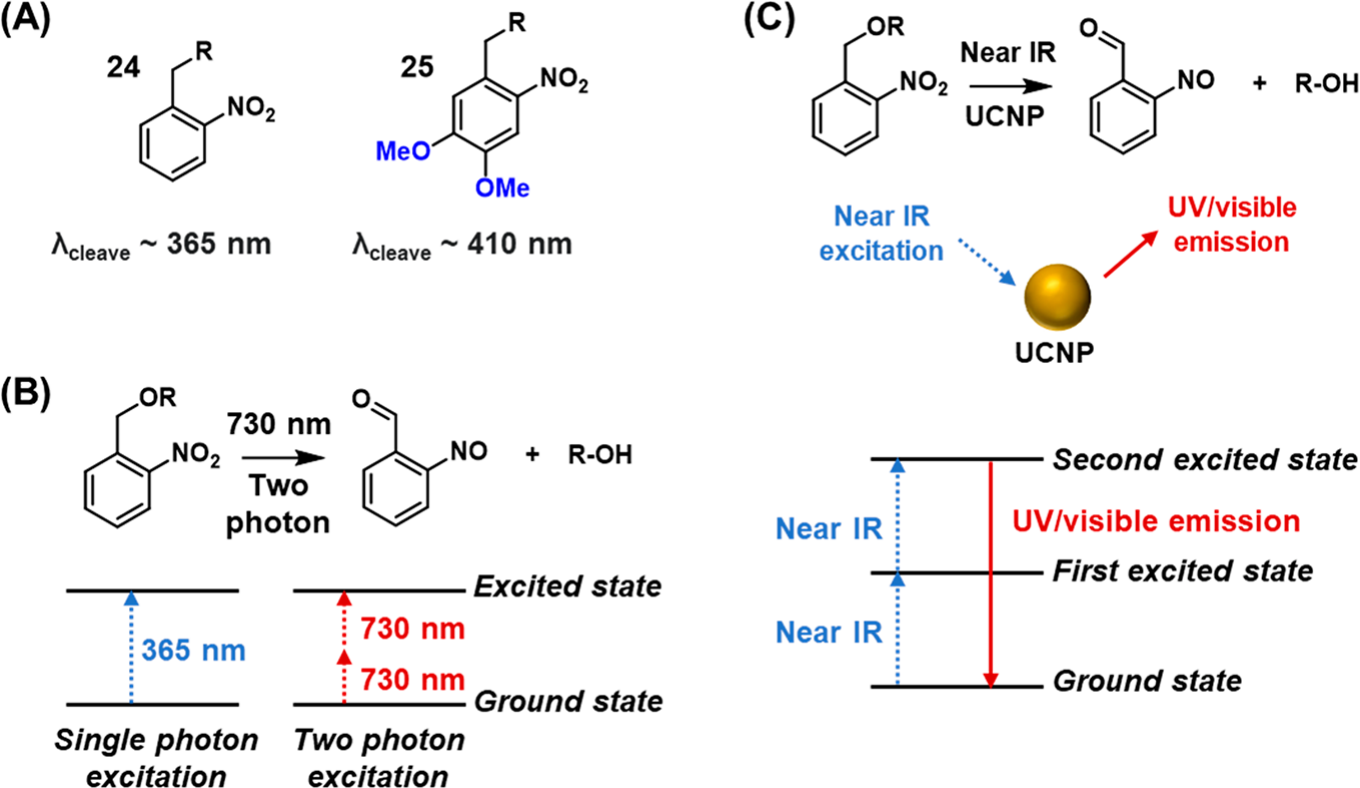
Photophysical control of the photochemical deprotection of ONB-protecting groups by (A) red-shifting the excitation wavelength using electron donating substituents associated with the benzene ring, (B) two-photon excitation of the ONB chromophore and (C) application of up-conversion nanoparticles.

One strategy for tuning the wavelength of absorption of ONB derivatives is through placement of substituents on the aromatic ring that influence the electronic structure of the chromophore, as detailed in recent reviews.^136,217^ Commonly, electron donating substituents such as methoxy groups are employed to achieve this effect, Figure 7A. For example, while unsubstituted compound **24** displays an absorbance up to ∼370 nm,^218^ the addition of two methoxy substituents (**25**) red-shifts the absorbance band, allowing photocleavage at wavelengths as long as 420 nm.^219^

An alternative strategy to allow the photocleavage of ONB protecting groups with longer wavelength light is to employ two-photon irradiation, in which the absorption of two lower-energy photons by the chromophore provides the total energy required for excitation. Thus, a caging group exhibiting one-photon absorbance at 365 nm may, thus, be excited by two photons of approximately twice the wavelength, ca. 730 nm, Figure 7B.^220−224^ Two-photon excitation is a nonlinear optical process and requires short-pulsed lasers to operate efficiently. However, compared to one-photon absorption processes, two-photon excitation confers several advantages in biological applications. First, the excitation wavelength is sufficiently long to allow deeper light penetration through biological tissue. Moreover, light scattering is reduced, facilitating increased contrast in imaging. Moreover, as the efficiency of the two-photon absorption process depends supra-linearly on the light intensity, higher spatial resolution may be obtained, as the illumination site is confined to the perifocal region of the laser beam.^225^

Another approach to trigger ONB cleavage using long-wavelength light involves the use of up-conversion nanoparticles (UCNPs), which are lanthanide-based materials that absorb two near-IR light photons and emit energy as a single UV photon.^226−228^ The upconversion process proceeds via several mechanisms such as excited state absorption, Figure 7C. Absorption of the first photon generates a metastable, long-lived first excited state. Absorption of a second photon generates a more highly excited state, resulting in the emission of a single photon at a shorter wavelength than the incident light. As the emitted light is within the absorbance of the ONB chromophore, the UV light required for photodeprotection is generated *in situ* via external long-wavelength irradiation. In the context of photoresponsive DNA nanotechnology, UCNPs confer further advantages as they may themselves be functionalized with the DNA structures of interest directly,^229^ and they possess further absorbance and fluorescence features that may be harnessed in tandem with DNA-based functionality in the development of highly sophisticated and sensitive devices. These three approaches toward triggering the photocleavage of ONB-containing nucleic acid structures are featured in the examples considered in the forthcoming chapters.

### 3.3 Photodeprotection Mechanism

**Scheme 3:**
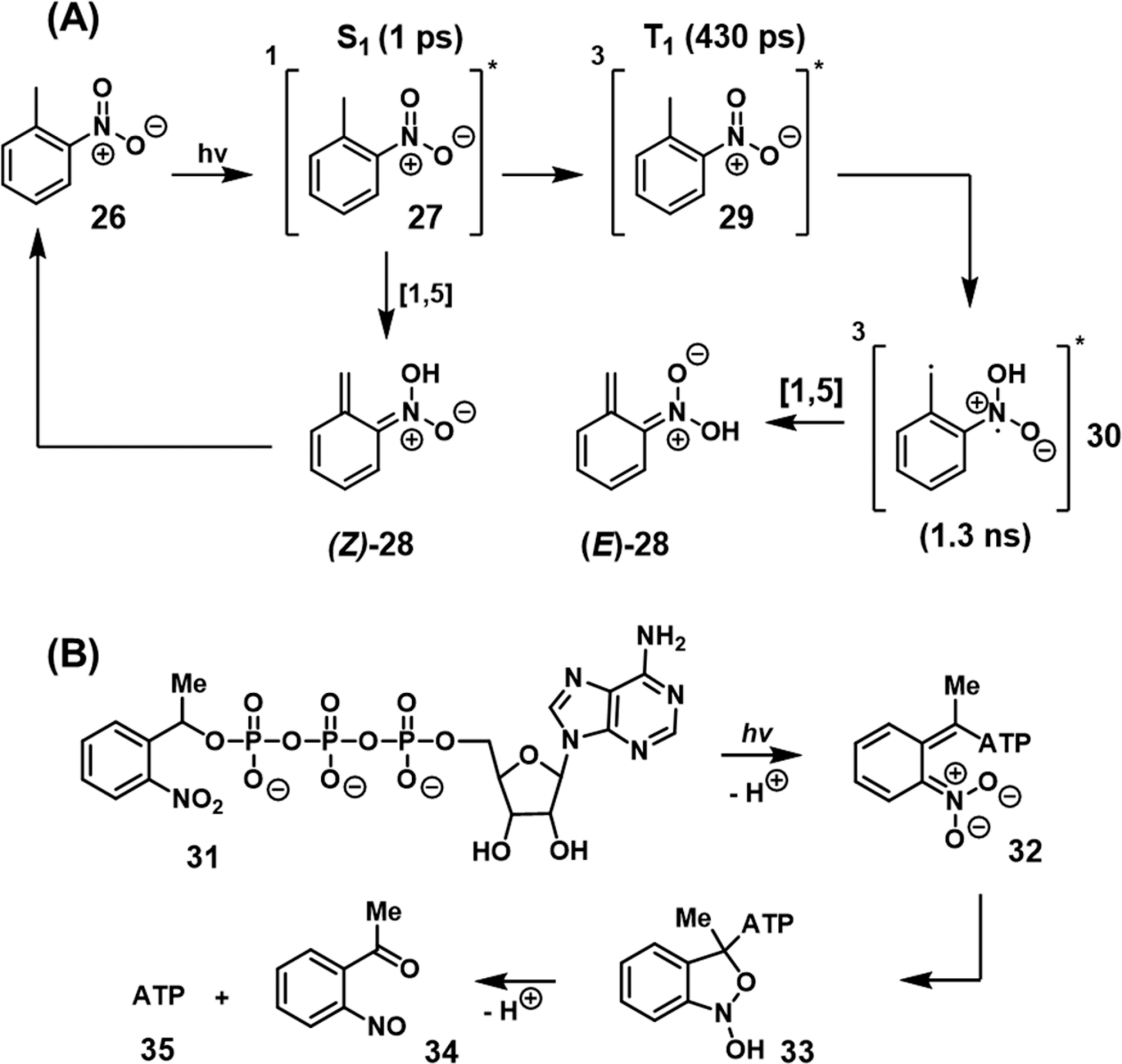
Mechanistic Issues Related to the Light-Induced Deprotection of the ONB Protecting Unit (A) Light-induced phototautomerization of the ONB into the *aci*-nitro intermediate. (B) Mechanistic steps involving the light-induced phototautomeriztion of the ONB-protected ATP to an *aci*-nitro intermediate that undergoes reconfiguration and dark degradation into the *o*-nitrosobenzophenone product, releasing free ATP.

The mechanism of photodeprotection of ONB-functionalized substrates is most often proposed to occur via phototautomerization of the nitrobenzyl group to an *aci*-nitro intermediate, which then decomposes to release the deprotected moiety.^230−233^ Femtosecond transient absorption and stimulated Raman spectroscopic studies of the parent compound, *ortho*-nitrotoluene **26**, allowed the direct probing of the excited-state intermediates leading to the *aci*-nitro species, Scheme 3A.^230^ Photoexcitation generates the short-lived (1 ps) excited singlet state **27** which decays either directly to the (*Z*)-*aci*-nitro species (*Z*)-**28** by [1,5] hydrogen transfer, or by intersystem crossing to the triplet state **29** (lifetime 430 ps) that may also undergo [1,5] hydrogen transfer to form the bis-radical species **30** which decays to the (*Z*)- or (*E*)-*aci*-nitro species. In the absence of a (protected) leaving group, the *aci*-nitro species has no decomposition pathway and instead back-tautomerizes to the starting compound. In protected compounds, subsequent dark-state reactions following phototautomerization release the deprotected product. For example, a study of the decomposition of caged ATP (**31**),^234^Scheme 3B, suggested that the decomposition of the *aci*-nitro intermediate **32** occurs via a hemiacetal intermediate **33** that decomposes to yield the deprotected ATP (**35**) and the nitrosophenone (**34)** byproduct.

## 4 Applications of Photocleavable DNA Nanostructures

### 4.1 Hairpin Chain Reactions and Fuel/Catalyst Driven Cycles

**Figure 8:**
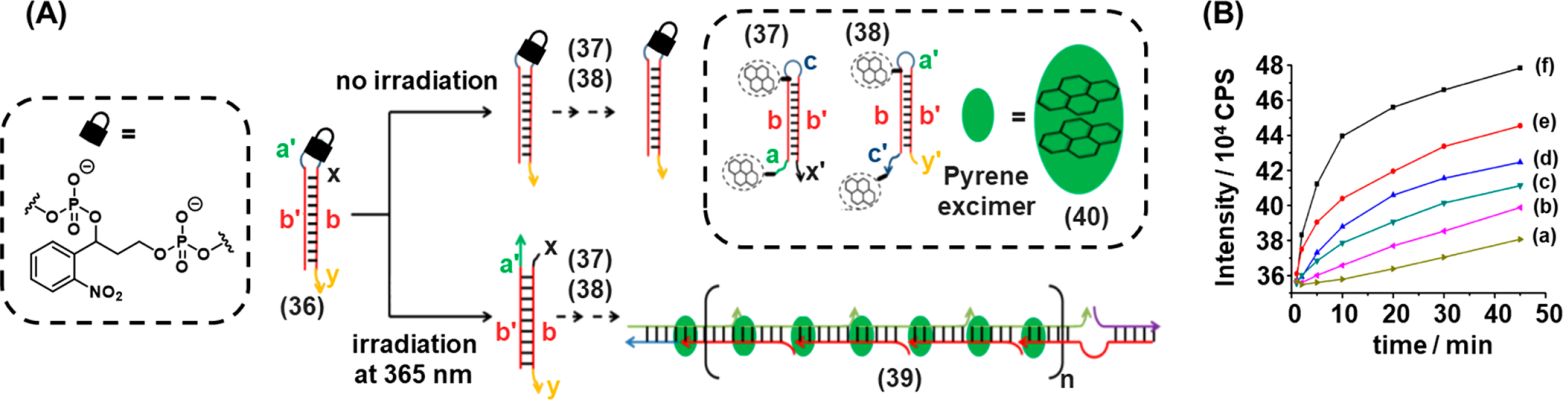
(A) Photochemical activation of an ONB-caged hairpin nucleic acid yielding a toehold-functionalized duplex to stimulate the hybridization chain reaction (HCR) between two pyrene-modified hairpins to yield oligomeric pyrene excimer structures. (B) Time-dependent exciplex emission intensities generated by the excimer oligomer formed upon the photochemical cleavage of the ONB-bridged activator for different time intervals: (a) 0 min, (b) 1 min, (c) 2 min, (d) 5 min, (e) 10 min, (f) 20 min. Figure adapted with permission from ref. (201). Copyright 2013, American Chemical Society.

The principle of phototriggered toehold-mediated strand displacement reactions introduced in Section 2.1 was applied to induce the development of a DNA hairpin chain reaction **(**Figure 8**)**_._(201) Hairpin (**36**) is engineered to contain two masked toeholds in the loop region (a′ and x), Figure 8A. These toeholds are designed to be complementary to single stranded regions of hairpin (**37**), which contains toeholds a and x′. In the initial state of the system, hairpin (**36**) is self-hybridized and inert to hairpin (**37**). Exposure of the system to UV light (λ = 365 nm) triggers the cleavage of the photolabile ONB linker in the loop region of hairpin (**36**), leading to the exposure of single-stranded toeholds a′ and x. Hybridization of toeholds a′ and x with their complementary toeholds in hairpin (**37**) triggers branch migration and the opening of hairpin (**37**). This results in the exposure of toehold (c) of hairpin (**37**), which, in turn, triggers the opening of hairpin (**38**). The subsequent exposure of toehold a′ in hairpin (**38**) triggers the opening of another hairpin (**37**) through hybridization with complementary domain a, leading to propagation of the hybridization chain reaction (HCR) and the formation of polymeric species (**39**). The strategic positioning of the pyrene moieties in hairpins (**37**) and (**38**) leads to the formation of a pyrene excimer complex (**40**) upon each propagation step, allowing the probing of the kinetics of the HCR process by following the temporal increase in excimer emission as a result of the HCR. Figure 8B depicts the time-dependent increase in emission intensity of the pyrene exciplex (λ_ex_ = 340 nm, λ_em_ = 475 nm) following the exposure of hairpins (**37**) and (**38**) to the activator strand (**36**) which was preirradiated for different illumination time intervals. The kinetics of the HCR become substantially faster as the preirradiation time of the hairpin (**36**) initiator is increased, demonstrating effective control of the activation of the system by the photocaging approach.

**Figure 9:**
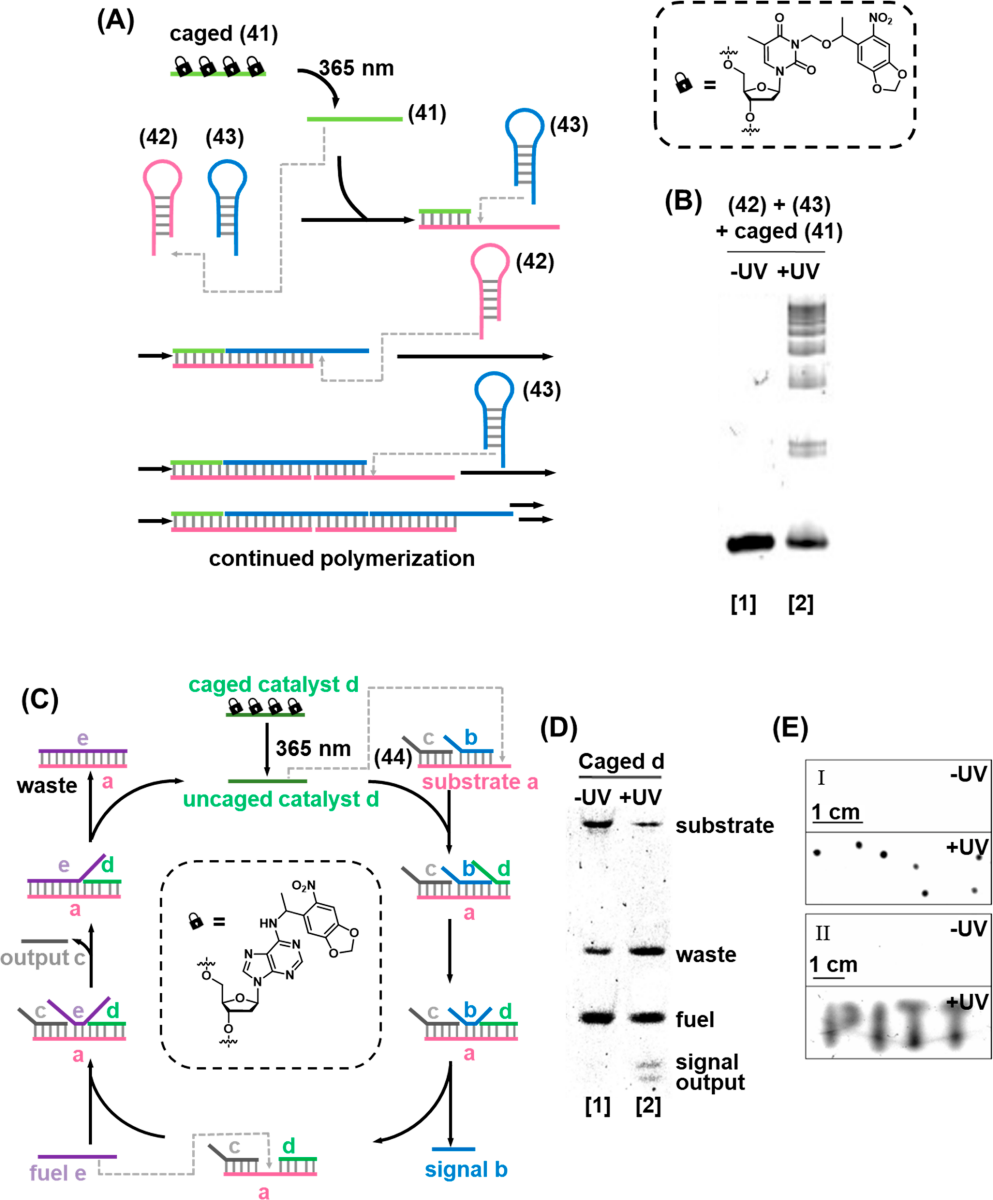
(A) Photodeprotection of an ONB-caged strand that initiates the HCR process yielding the oligomerized duplex strand HCR product. (B) Electrophoretic separation of the resulting HCR products without (Lane 1) and with (Lane 2) light-induced activation of the HCR process. (C) Photochemically triggered activation of a fuel/catalyst driven toehold-mediated strand displacement cycle using the light-induced deprotection of an ONB-functionalized strand as the activator. (D) Imaging the light-induced fuel-driven strand displacement cycle by electrophoretic separation. (E) On-gel patterning of dots (Panel I) and text (Panel II). Figure adapted with permission from ref. (202). Copyright 2015, American Chemical Society.

Photocaged single-stranded nucleotides of the type discussed in Section 2.2 have also been applied in the regulation of HCRs, Figure 9A.^202^ In the rest state, self-hybridized hairpins (**42**) and (**43**) were inert, since the trigger stand (**41**) was inactivated by photocaging of four thymine base constituents with ONB moieties, which prevented the strand from forming duplex hybrids. Irradiation of the system with UV light triggered the photocleavage of the protecting groups, activating the strand toward hybridization with the toehold of hairpin (**42**), triggering the HCR to generate polymeric duplexes, which was observed by gel electrophoretic separation from the monomeric precursors, Figure 9B. When hairpins (**37**) and (**38**) were exposed to the caged-(**41**) strand, the system remained dormant in the absence of UV light (Lane 1). Irradiation of the system with UV light triggers the uncaging of (**41**) and bands corresponding to the HCR products were observed (Lane 2).

Photocaged single-stranded DNA was also used as the activator of a fuel/catalyst cycle, Figure 9C. A duplex hybrid (**44**), consisting of a substrate strand a, signal strand b, and output strand c, was designed. The substrate strand was engineered to contain a single-stranded toehold complementary to a region of the catalyst strand d, which was gated by installation of photolabile ONB caging groups on adenine bases. Upon photocleavage of the ONB, the catalyst strand d was activated and hybridized with the available toehold of substrate strand a, triggering the reaction cycle and resulting in the buildup of signal and output strands which were observed by gel electrophoresis, Figure 9D. Addition of the caged catalyst (d) results in no evolution of output/signal strands, demonstrating the effective photocaging of the single-stranded catalyst by the nitrobenzyl moieties (Lane 1). Upon irradiation with UV light, the caging groups were removed and the active catalyst was generated to trigger the reaction cycle leading to the release of output and signal strands (Lane 2). The operation of the system in the semisolid media of a low-melt agarose gel was also demonstrated, Figure 9E, irradiating either localized regions to create dots (Panel I) or through a mask to create an image (Panel II). A photocaged inhibitor strand was also introduced that enabled the photochemical “switch off” of the catalytic cycle.

### 4.2 RNA Detection and Imaging by ONB-Functionalized Scaffolds

The selective and sensitive detection and imaging of messenger RNA (mRNA) and microRNAs (miRNAs) in living cells is a key challenge toward understanding the role of these biomarkers in regulating cellular processes related to diverse diseases^235−238^ and in the development of new diagnostic and therapeutic technologies.^239−241^ Nucleic acids are versatile motifs for the design of such sensors, owing to robust synthetic methods, predictable base pairing properties, and ease of functionalization that allows the specific targeting of the desired intracellular analytes.^211^ DNA-based replication machineries also offer several opportunities for signal amplification strategies, such as the HCR,^196,242,243^ polymerase chain reaction (PCR),^244,245^ and rolling circle amplification (RCA),^246,247^ which allow a significant increase in readout sensitivity. Moreover, nucleic acids may be readily conjugated to a variety of other chemical functionalities such as molecular fluorophores^174,248,249^ and fluorescent nanoparticles^250,251^ which provide a means to generate different optical signal readouts. A wide variety of DNA-based sensing technologies for intracellular analytes have been realized using these approaches.^252−254^ A key limitation of many devices, however, is that the sensing module normally exists in an “always on” state, which is a severe limitation to their utility in cellular models and *in vivo* as microRNA expression is a dynamic, transient process regulated in specific cells. Thus, to obtain a complete understanding of the role of different miRNAs in the regulation of biological events, spatiotemporal resolution in detection is required. It is therefore desirable for the device to be administered in a temporarily masked state until activation is required in the target region. Light is an ideal stimulus for the triggered activation of such devices, and, in recent years, significant progress has been made toward engineering photocaged DNA-based sensors for miRNA sensing and imaging.^200,255−257^ The examples discussed below highlight recent advances toward this goal.

**Figure 10:**
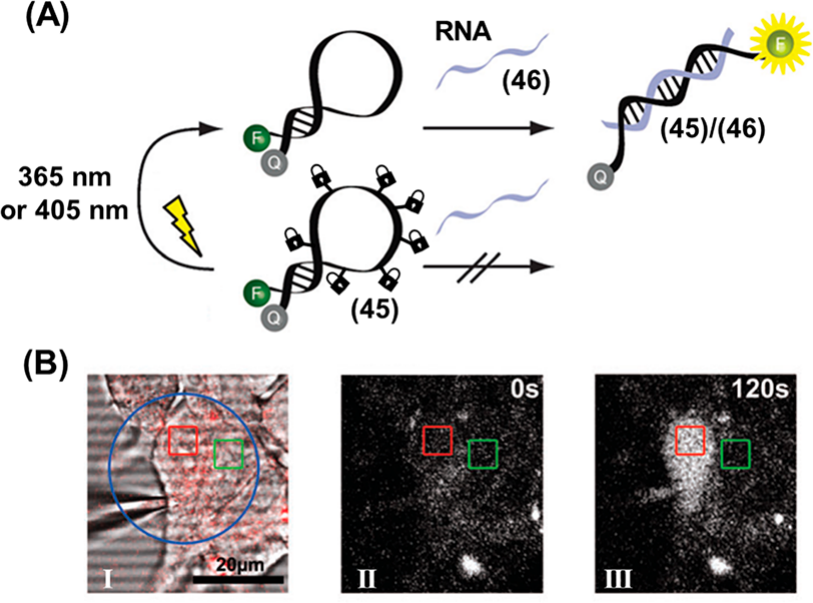
(A) Fluorescence imaging of an mRNA analyte by the light-induced deprotection of an ONB-protected fluorophore/quencher labeled hairpin sensing probe. (B) Intracellular detection of the mRNA by the light-induced activation of the ONB-protected hairpins incorporated in the cell environment. Panel I: map of irradiation site (blue circle), probe-treated cell (red square) and untreated cell (green square). Panel II: intracellular beacon fluorescence prior to photoirradiation. Panel III: intracellular beacon fluorescence after photoirradiation. Figure adapted with permission from ref. (258). Copyright 2012, Royal Society of Chemistry.

Figure 10 demonstrates the use of photocaged nucleobases to control a hairpin-based RNA sensing device.^258^ A molecular beacon (**45**) for glyceraldehyde-3-phosphate dehydrogenase (GAPDH) mRNA consisting of a self-complementary fluorophore-quencher labeled molecular beacon was engineered to contain photocaged nucleobases in the single-stranded loop that prevent target hybridization, Figure 10A. Photoirradiation triggers the cleavage of the caged moieties, leading to the active loop hybridizing the target RNA (**46**), triggering the opening of the hairpin and leading to the activation of fluorescence of the beacon. The beacon was used to detect the target analyte at the single-cell level, Figure 10B. A single human embryonic kidney (HEK 293) cell was loaded with caged beacon **45** (Panel I, red square) while a neighboring control cell (Panel I, green square) was left untreated. Prior to photoirradiation, no beacon fluorescence was observed in either cell (Panel II). After photoirradiation of a region containing both cells (Panel I, blue circle), the beacon fluorescence was rapidly activated in the target cell, while the neighboring cell (containing no beacon) remained dark (Panel III).

**Figure 11:**
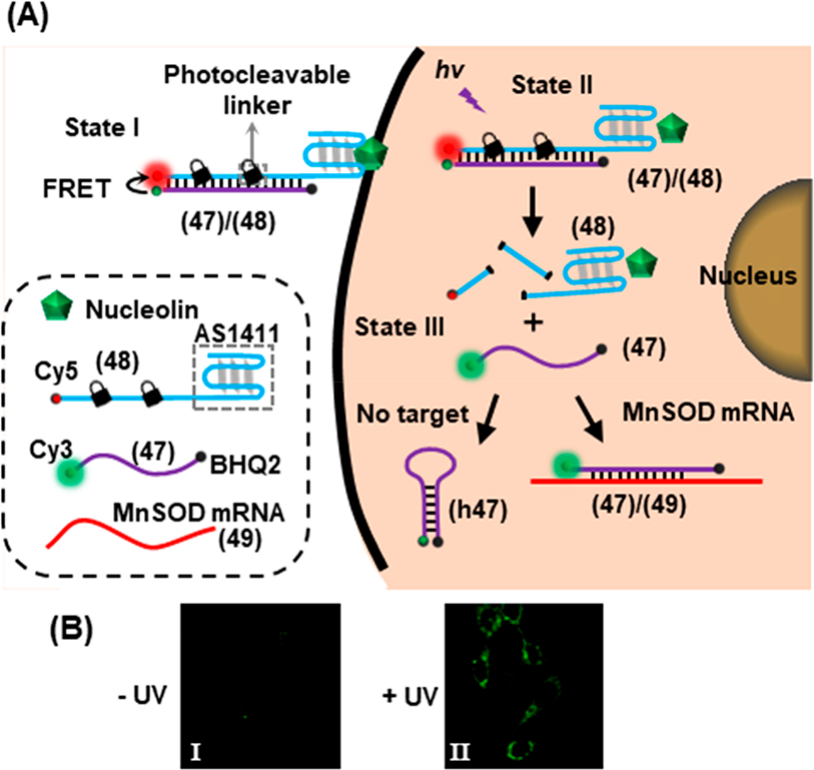
(A) Schematic integration of an ONB-photoprotected fluorescent probe into cancer cells for the temporal activated sensing of the MnSOD mRNA. The light-induced cleavage of the probe yields a fluorescent quenched hairpin product in the absence of target. In the presence of the mRNA target the fluorescence of the remains switched on. (B) Intracellular activation of fluorescence imaging of intracellular mRNA upon light-induced activation of the probe. Figure adapted with permission from ref. (259). Copyright 2013, American Chemical Society.

Photocleavable DNA duplexes have also been applied in the control of fluorescent molecular beacons for mRNA detection in living cells.^259^Figure 11A depicts a duplex probe (**47**)/(**48**) designed to incorporate a fluorescent donor (Cy3) at one end and a quenching moiety (BHQ2) at the other end, and its sequence chosen to be complementary to MnSOD RNA as analyte (**49**). Strand (**48**) contained the quenching (Cy5) fluorophore and the AS1411 nucleolin binding G-quadruplex aptamer. The aptamer served to guide aid the internalization of the probe into the tumor cells that overexpress the nucleolin receptor on the cell surface. Strand (**48**) also contained ONB-based photocleavable linkers that enabled the spatiotemporal activation of the probe in the cells of interest. Upon photoirradiation with UV light (State II), the ONB moieties of strand (**48**) are cleaved and the resulting short strands dissociate from strand (**47**), triggering the release of free strand (**47**) (State III). Sequestration of the released strand (**47**) by the target RNA (**49**) to form duplex (**47**)**/**(**49**) prevents the formation of self-complementary hairpin (**h47**) resulting in the activation of Cy3 fluorescence. The performance of the photoresponsive probe was evaluated in live cells, Figure 11B. The Cy3 fluorescence remained quenched by the intact probe in the absence of photoirradiation (Panel I). Upon irradiation with UV light (302 nm, 1.06 W, 10 min), the Cy3 fluorescence was activated in response to the RNA target (Panel II).

**Figure 12:**
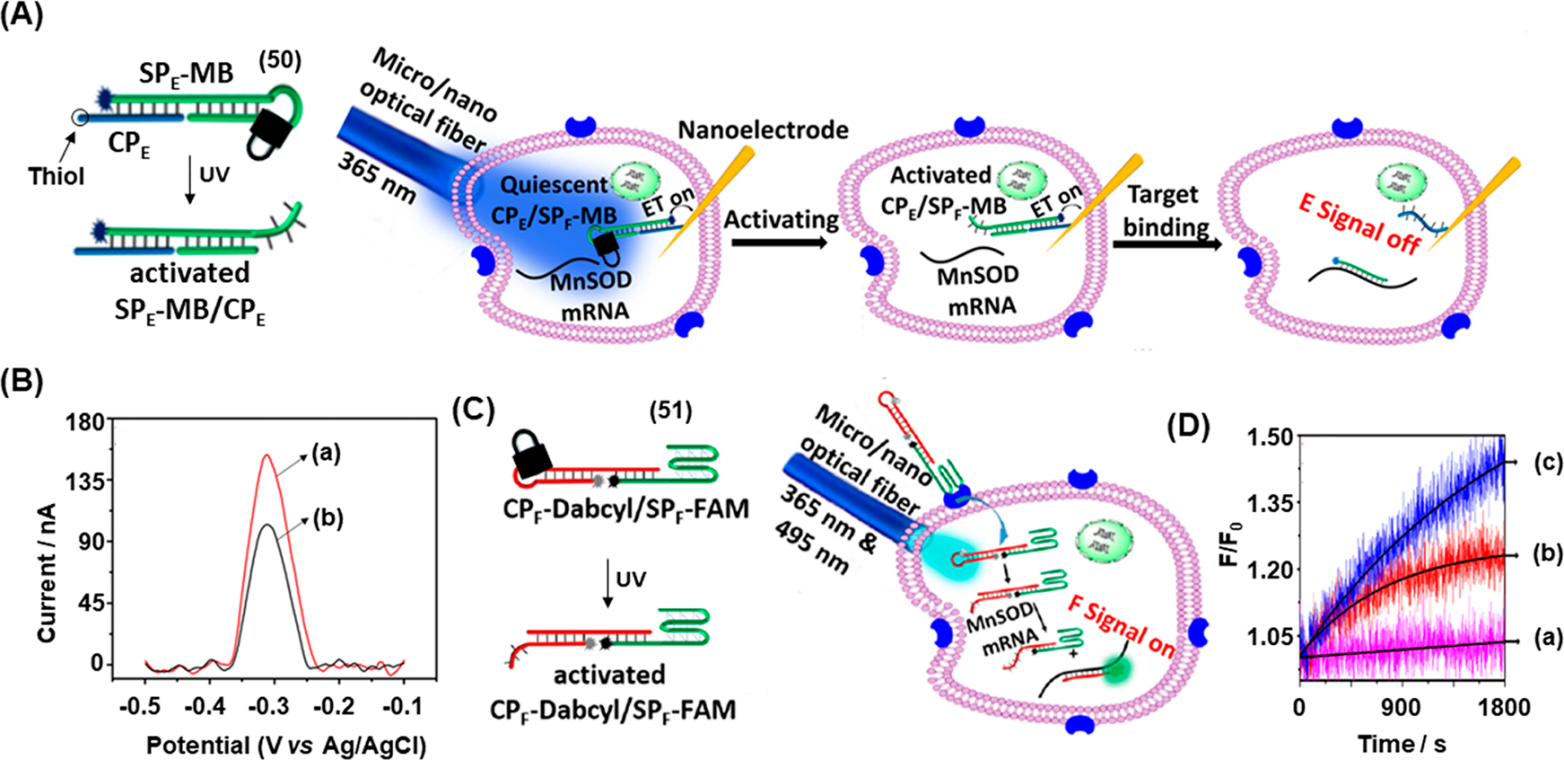
(A) Light-triggered (365 nm) electrochemical sensing of MnSOD mRNA at the single-cell level using a methylene blue (MB) functionalized ONB-caged hairpin-modified nanoelectrode. (B) Intracellular voltammetric responses of the MB-functionalized ONB-caged hairpin-modified electrode: (a) before irradiation, (b) after irradiation and mRNA-induced displacement of the fragmented hairpin. (C) Intracellular detection of MnSOD mRNA using an ONB-caged FAM-modified hairpin hybridized to a dabcyl-quencher-modified strand. (D) Time-dependent fluorescence intensities upon sensing intracellular MnSOD mRNA by the optical probe: (a) without light-triggered cleavage of the hairpin, (b) after light-induced cleavage of the hairpin, (c) after upregulation of the MnSOD mRNA using LPS and light-induced cleavage of the probe. Figure adapted with permission from ref. (255). Copyright 2018, American Chemical Society.

Figure 12 depicts a further live-cell mRNA detection strategy based on the photoactivated toehold-mediated strand displacement reaction.^255^ This system employed two types of photocleavable DNA modules in order to provide a dual readout signal for the analyte. One type of module, SP_E_-MB/CP_E_ (**50**), was engineered to provide an electrochemical signal and another, CP_F_-Dabcyl/SP_F-_FAM (**51**), was designed to generate a fluorescence signal, with both readout modules activated by photocleavage of the constituent ONB-containing hairpin loop. Each module contained a capture probe (CP) hybridized to signal probe (SP). The electrochemical system is depicted in Figure 12A. The capture probe (CP_E_) of unit (**50**) was functionalized with a thiol moiety to allow immobilization on a gold-coated nanoelectrode. CP_E_ is hybridized to the signal probe strand (SP_E_) that was engineered to contain a photocleavable hairpin loop (containing a masked toehold complementary to the mRNA analyte, MnSOD) and functionalized with a methylene blue (MB) reporter. The nanoelectrode tip was sufficiently small (78 nm in diameter) to allow insertion into a single cell with precision. Prior to photoirradiation, the MB-functionalized electrode generated a voltametric response, Figure 12B, curve (a). Irradiation of the cell with UV light using a 5 μm optical fiber triggered the cleavage of the ONB moiety, leading to the opening of the hairpin loop and the unmasking of the toehold. In this state, the mRNA analyte effected the displacement of the redox-active MB-functionalized signal strand, SP_E_-MB, from the electrode and the observed electrochemical signal decreased by approximately 33%, curve (b). The fluorescence readout system is shown in Figure 12C. In this case, the signal probe (SP_F_-FAM, **51**) was engineered to contain the G-quadruplex-forming AS1411 nucleolin aptamer, allowing efficient uptake by cancer cells. The strand was functionalized at the 5′ end with a fluorophore (FAM) to act as the readout signal. The fluorescence was inactivated by hybridization of the strand to the capture probe strand (CP_F_-Dabcyl) engineered to contain a fluorescence quencher. This strand also contained the photocleavable ONB moiety in its hairpin loop. Thus, photocleavage of the loop inside the cell activates the quencher strand toward toehold mediated strand displacement by the MnSOD analyte, separating the fluorophore/quencher pair and resulting in fluorescence signal turn-on. Figure 12D shows the time-dependent change in fluorescence intensity under different conditions. In the absence of UV light, no increase in probe fluorescence was observed, curve (a), as the hairpin loop remains intact and the strand was inert to the presence of the MnSOD mRNA. Following UV irradiation of a single cell using an optical fiber, however, a significant increase in fluorescent signal was observed over 30 min as the analyte displaces the quenching strand from the activated probe, curve (b). Moreover, upon treating the cells with lipopolysaccharide (LPS) to upregulate the level of MnSOD RNA, an enhanced fluorescence signal was observed, curve (c), demonstrating the sensitivity of the system to the expression level of the MnSOD RNA target.

**Figure 13:**
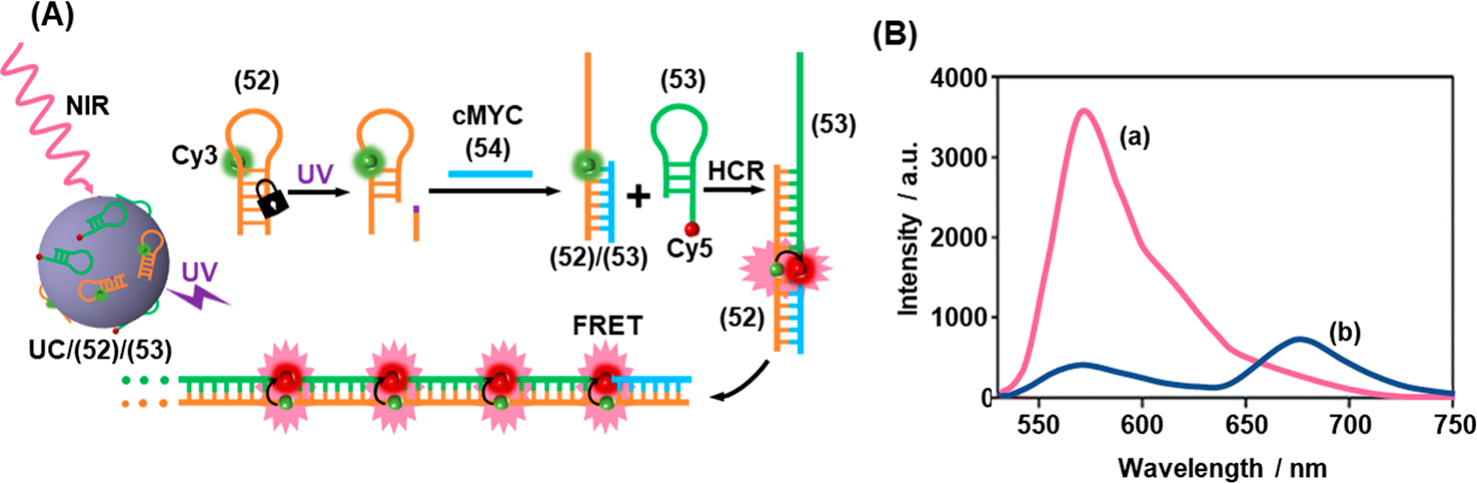
(A) Intracellular amplified sensing of cMYC mRNA by UCNPs functionalized with an ONB-caged Cy3-modified hairpin. In the presence of an auxiliary Cy5-functionalized hairpin, the NIR-triggered UC-stimulated cleavage of the ONB-caged hairpin leads to a fragmented hairpin being opened by the c-MYC mRNA, a process initiating the HCR with the auxiliary hairpin, resulting in the Cy3/Cy5 FRET process providing the sensing readout signal. (B) Fluorescence output of the sensing module: (a) before irradiation and (b) after irradiation, resulting in the Cy5 FRET signal. Figure adapted with permission from ref. (260). Copyright 2019, John Wiley and Sons.

Photogated HCRs have also been deployed to enhance readout sensitivity of mRNA probes, Figure 13.^260^ In this case, the photocleavable moiety was incorporated into the stem region of the hairpin (**52**), Figure 13A. Cleavage of this moiety by UV light either directly, or by the UV luminescence of a UCNP irradiated using near-IR light, generates a six-base toehold at the 5′ end of the hairpin (**52**), which is programmed to recognize c-MYC mRNA (**54**). Thus, the analyte triggers the opening of the hairpin by toehold-mediated strand displacement, resulting in the activation of the (**52**)/(**53**) HCR to generate a polymeric duplex that forms spatially proximate Cy3/Cy5 FRET pairs upon each cycle of the HCR. Figure 13B depicts the fluorescence spectra of the system under different conditions upon exciting the donor (Cy3) fluorophore. As the hairpin (**52**) is activated only under photocleavage, the FRET signal (Cy5 fluorescence at 675 nm) emerges only when the system is subjected to both photoactivation (UV) and the presence of the target c-MYC analyte, curve (b). In the absence of photoirradiation, only Cy3 fluorescence is observed, curve (a). The high signal amplification afforded by the photoactivated chain reaction allowed a sensitive detection limit of 0.6 pM to be achieved. The system proved capable of phototriggered sensing of cMYC expression levels in MCF7 cells.

**Figure 14:**
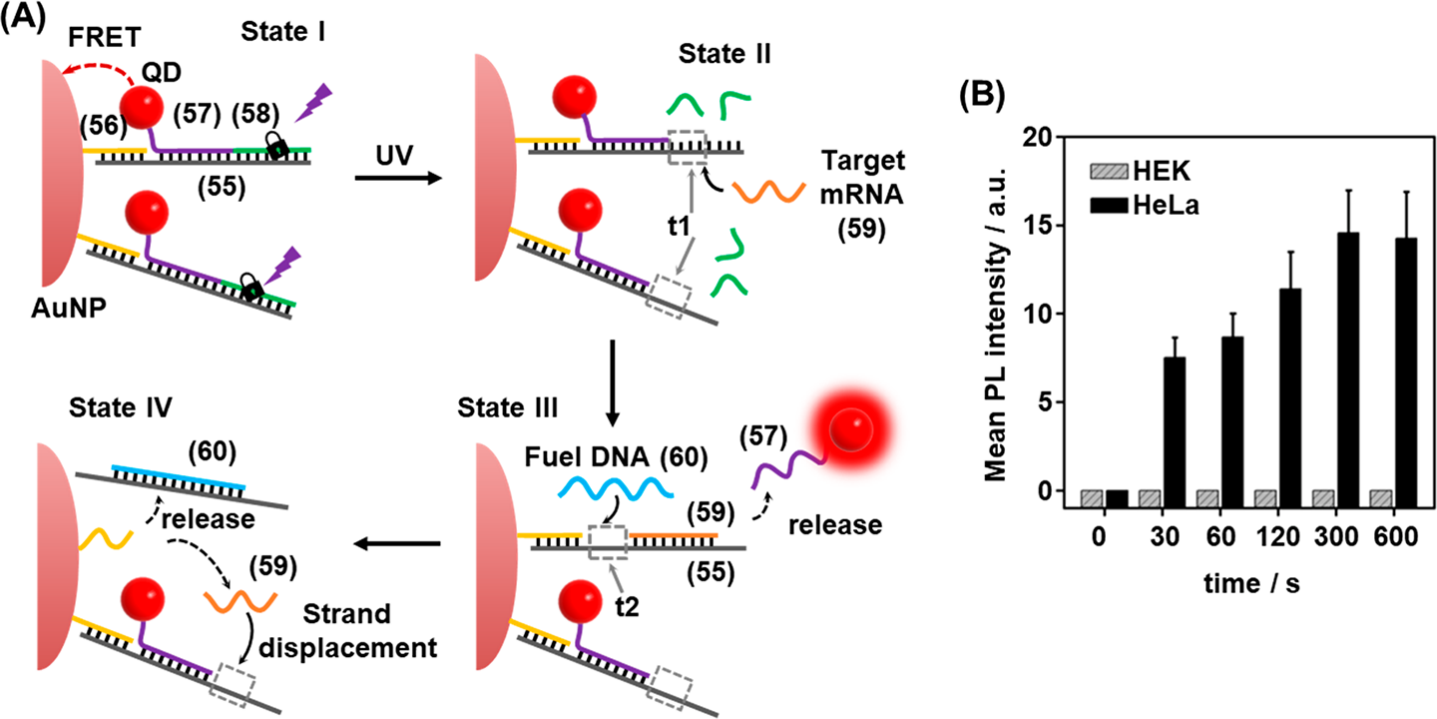
(A) Schematic amplified detection of miRNA in HeLa cells using an ONB-gated CdTe QD/AuNP conjugate. (B) Amplified luminescence signals observed upon sensing miRNA-21 with the Au nanoparticle/CdTe quantum dots in miRNA-containing HeLa cells and miRNA-negative HEK cells upon light-induced uncaging of the sensing probe for different time intervals. Figure adapted with permission from ref. (200). Copyright 2018, American Chemical Society.

Photocleavable DNA duplexes were, also, integrated with gold nanoparticles (AuNPs) for the imaging of miRNA in malignant cells (Figure 14).^200^ The sensor consisted of a AuNP functionalized with quantum dots (QDs) within an ONB photocleavable nucleic acid construct, Figure 14A. A nucleic acid scaffold (**55**) was hybridized with nucleic acid (**56**)-functionalized AuNPs. The scaffold was further functionalized through hybridization with nucleic acid (**57**)-modified QDs and the ONB-protected strand (**58**), State I. The spatial proximity between the AuNP and the QD components led to effective quenching of the luminescence of the QDs in the sensing construct. Photochemical cleavage of the constituent (**58**) led to the separation of the fragmented products of insufficient duplex stability, leading to the toehold (**t1**) functionalized scaffold in State II. The toehold domain was, however, pre-engineered to include the sequence that is partially complementary to the target miRNA-21 (**59**). In the presence of the target miRNA, the toehold-mediated displacement of the (**57**)-modified QDs proceeded, where the target miRNA was hybridized with the (**55**) scaffold, State III. Hybridization of the miRNA with the scaffold yielded, however, a pre-engineered single stranded domain (**t2**) in State III. In the presence of an auxiliary fuel strand (**60**), the domain **t2** provided active sites for the toehold displacement of the target miRNA and the generation of State IV, where miRNA was released. The released miRNA was then utilized to displace further QDs associated with the AuNPs. The release of the QDs from the AuNPs support switches ON the luminescence of the QDs that provides a readout signal for sensing the miRNA. As each of the AuNPs was functionalized with multiple QDs, amplified detection of the miRNA, in the presence of the auxiliary fuel strand (**60**), was accomplished. Indeed, the target miRNA was analyzed with a detection limit corresponding to 10.4 pM. The photoactivated probes proved capable of sensing miRNA-21 in live cells. Figure 14B shows the effect of irradiation time on the observed signal intensity. Here, 300 s of irradiation was sufficient to generate the maximal signal output in the case of HeLa cells, while for miRNA-21-negative HEK cells, no signal was observed even after 600 s of photoirradiation.

**Figure 15:**
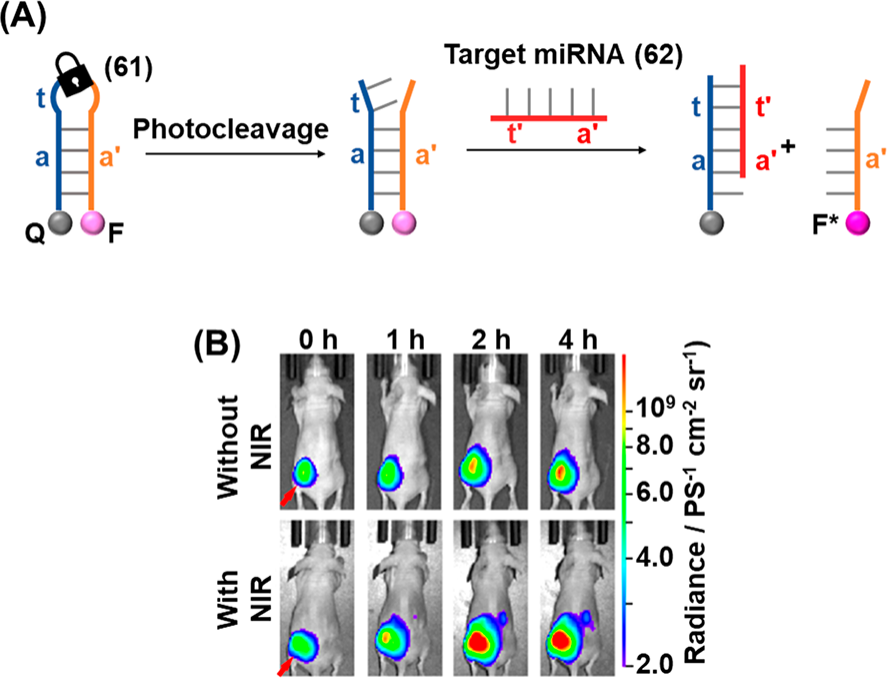
(A) Photochemically triggered activation of an ONB-protected fluorophore/quencher caged hairpin miRNA sensing probe. (B) Application of the probe shown in (A) for the *in vivo* detection of miRNA-21 present in HeLa tumors in mice. Coupling of the probe UCNPs allowed NIR-triggered activation of the detection module. Images of probe fluorescence correspond to different time intervals in the absence of probe activation (upper) or following probe activation with NIR light (lower). Figure adapted with permission from ref. (257). Copyright 2019, American Chemical Society.

A near-IR light-controlled DNA nanodevice for miRNA detection and imaging in live cells based on UCNPs was developed that allowed *in vivo* phototriggered imaging, Figure 15.^257^ A DNA hairpin (**61**) was engineered to include a domain (a) partially complementary to the sequence of the desired miRNA analyte (**62**). The hairpin featured a photolabile ONB moiety in the loop region that also contained a masked toehold (t) sequence revealing partial complementarity to the miRNA analyte, Figure 15A. The hairpin was functionalized at its ends with a Cy5 fluorophore (F) and the black-hole quencher (Q), leading to quenched Cy5 fluorescence of the hairpin sensing probe. Irradiating the system with UV light (λ = 365 nm) results in cleavage of the hairpin loop, which presents a single-stranded toehold region (t) engineered to hybridize with the target miRNA, leading to displacement of the fluorescent strand and switch-on of Cy5 fluorescence (F*). To allow the detection of the target miRNA in biological samples, the sensing platform was activated by Gd/Yb/Tm UCNPs. Upon excitation with NIR light (λ = 980 nm), two-photon absorption led to localized nanoparticle luminescence at shorter wavelengths (λ = 346, 363, 453, 478 nm), thus circumventing the requirement for UV irradiation to activate photocleavage. The UCNPs were functionalized with photoactivatable detection module (**61**) by passivation of the particles with cationic polylysine layer, allowing attachment of the negatively charged DNA strand (**61**) through electrostatic interactions, affording a loading level of 30 probes per nanoparticle. The probe-functionalized UCNPs were injected into murine HeLa tumors and the mouse imaged by whole-body fluorescence measurements, Figure 15B. In the absence of near-IR light, only a minimal increase in probe fluorescence at the tumor site was observed after 4 h, demonstrating the system remains dormant until photoactivation. However, following activation of the internalized probe with near-IR light, a significant increase in the intensity of the probe fluorescence at the tumor site was observed. After 2 h, the observed probe fluorescence was approximately 2-fold higher in the tumor region following near-IR irradiation, as compared to the nonirradiated control. These results demonstrate that near-IR light can be used for the effective activation of the probes *in vivo,* owing to its effective penetration through biological tissue. The UCNP hairpin conjugates were also able to detect the tumoral miRNA when administered intravenously, thus showing potential practical diagnostic applications.

**Figure 16:**
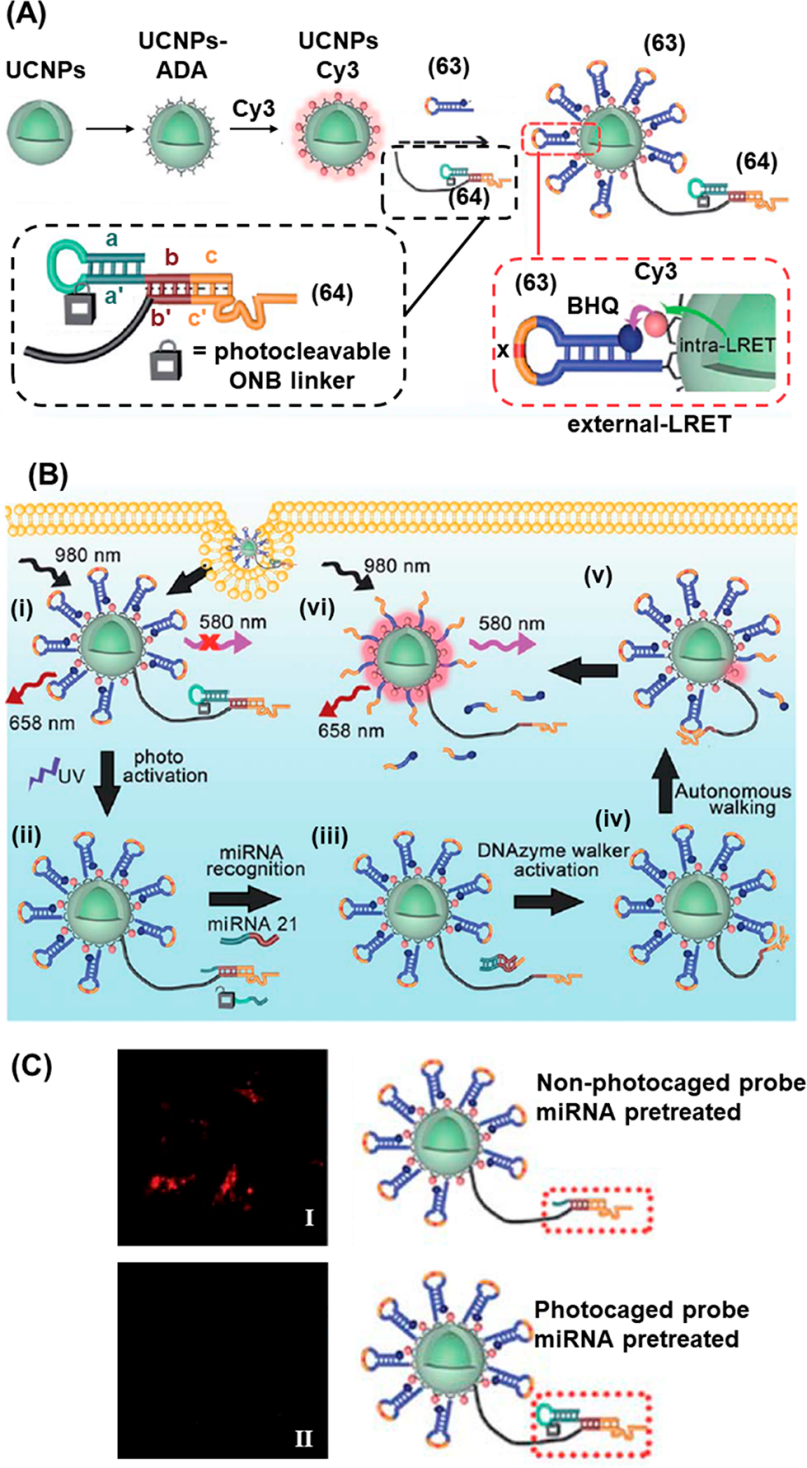
(A) Schematic synthesis of Cy3-modified UCNPs cofunctionalized with quencher-modified hairpin strands and ONB-caged DNAzyme units for the light induced deprotection of the DNAzyme structure that triggers the cleavage of the quencher-modified hairpin in the presence of the miRNA target, activating Cy3 fluorescence. (B) Intracellular application of the functionalized UCNPs for detection of miRNA-21 via the photochemical uncaging of the DNAzyme and miRNA-guided activation of the DNAzyme. (C) Confocal fluorescence imaging of miRNA-21 negative cells treated with (Panel I) uncaged UCNP probes pre-exposed to miRNA-21 to simulate extra-cellular activation, demonstrating false-positive intracellular fluorescence readout, resulting from extracellular miRNA-21 activation and (Panel II) photocaged UCNP probes pretreated with miRNA-21, demonstrating effective caging of the probes and elimination of false-positive intracellular fluorescence. Figure adapted with permission from ref. (229). Copyright 2020, Royal Society of Chemistry.

An miRNA sensing device of enhanced complexity combining the photoactivated toehold-mediated strand displacement method with DNAzymes and UCNPs is depicted in Figure 16.^229^ This system makes use of the luminescence properties of the UCNPs, that upon near-IR light excitation at 980 nm yield two distinct emission bands at 658 and 540 nm, where the latter fluorescence band may activate fluorescence of a proximal Cy3 fluorophore by luminescence resonance energy transfer (LRET). Following the preparation of NaYF_4_:Yb,Er,Gd@NaYF_4_ nanoparticles, they were functionalized with amine groups by ligand exchange with alendronic acid (ADA) on the nanoparticle surface, Figure 16A. The amine groups were used as reactive handles to conjugate the UCNPs to the Cy3 dye and the PEG-maleimide coupling handle, that enabled the conjugation of thiol-functionalized DNA strands (**63**) and (**64**) at a 10:1 ratio. Strand (**63**) contained a hairpin unit (x) engineered to be cleaved by the Mn^2+^ dependent DNAzyme (*vide infra*) and was end-functionalized with a black-hole quencher (BHQ) in order to inactivate the luminescence of the Cy3 dye on the surface of the UCNP (itself activated from LRET from the 580 nm emission band of the UCNP). Meanwhile, strand (**64**) contained the corresponding Mn^2+^ dependent DNAzyme (c′) engineered to cleave the target strand, separated from the UCNP by a 40 nt poly-T spacer in order to allow spatial motility. Critically, the activity of the DNAzyme was blocked by hybridization to a photozipper unit comprising the self-complementary (aa′) hairpin engineered to contain the photoresponsive ONB fragment in the loop region and hybridized with the DNAzyme/spacer strand through the bc/b′c′ duplex, preventing the activity of the DNAzyme. Prior to photoirradiation, the photozipper assembly was stable in the presence of the miRNA target sequence, Figure 16B, State i. Irradiation of the dormant inactive system with 980 nm light led to UCNP luminescence at 658 nm. Meanwhile, the 540 nm UCNP emission band excited the Cy3 fluorophore on the surface, yet its emission was quenched by the proximal BHQ, resulting in the blockage of the Cy3 fluorescence (at 580 nm). Upon illumination with UV light, however, the ONB moiety in the strand (**64**) hairpin loop was cleaved, leading to dissociation of the a/a′ duplex (State ii). The target miRNA hybridized with the single stranded a′ region (acting as a toehold), triggering the strand displacement of the Mn^2+^-dependent DNAzyme walker strand, resulting in catalytic activity (State iii). Subsequent DNAzyme-mediated cleavage of the strand (**63**) hairpin (State iv) destabilized the duplex structure, causing dissociation of the constituent strands and the separation of the BHQ quencher units from the UCNP surface. Thereby, the fluorescence capacity of Cy3 was restored (State v). Excitation of the UCNP by 980 nm light therefore led to the observance of the 580 nm LRET band of Cy3 in addition to the 658 nm band observed prior to irradiation (State vi). Because of the large number of Cy3 fluorophores on the surface of the UCNP, and the flexibility afforded by the poly-T spacer unit, a single miRNA recognition event triggers a large number of cleavage reactions, affording a high level of signal amplification. Using this strategy, a detection limit of 3.71 pM target was achieved, where the sensitivity of the system resulted from the high level of signal amplification afforded by the DNA walker strategy.

The utility of the LRET-guided activated 540 nm fluorescence of the Cy3 fluorophore by the (**63**)/(**64**) functionalized nanoparticles for imaging intracellular miRNA-21 in cells was demonstrated. The critical role of the photoresponsive unit is to prevent the system being triggered by extracellular miRNA prior to internalization in the target cells, eliminating a loss of resolution resulting from background activation, Figure 16C. When nanoparticles were pretreated with solutions of miRNA-21, in order to simulate extracellular probe activation, false-positive fluorescence was observed in miRNA-21 negative cells exposed to nanoparticles lacking the photoresponsive protecting loop (Panel I). In the case of the photocaged nanoparticles, the presence of the photocleavable hairpin moiety guards the nanoparticles against unwanted activation by extracellular target and the false-positive result was not obtained (Panel II).

### 4.3 Protein Synthesis and Gene Expression Guided by Photodeprotection of ONB-Functionalized Nanostructures

Beyond the sensing technologies introduced in the previous section, another application of the miRNA recognition capabilities of photoresponsive DNA structures is the regulation of gene expression by mRNA silencing, which has potential to control organism development and regulate health and disease pathways.^261−263^ Different methods have been introduced to regulate gene expression in cellular models and even in whole organisms. These include nucleic acid strands engineered to bind and inhibit the translation of mRNAs, including siRNA^264,265^ and morpholino compounds,^266^ that demonstrated effective gene knock-down performance. However, in the complex milieu of the cell, the expression of genes often takes place in a transient time-dependent manner, and means to control the spatiotemporal activation of gene-expression are highly desirable, for example to activate gene knockdown during a particular phase of the cell cycle.^267^ To address this limitation, the possibility to engineer photocleavable nucleic acid strands containing ONB moieties has received substantial research attention as a means to control the activity of gene knockdown agents *in vitro* and *in vivo*.

**Figure 17:**
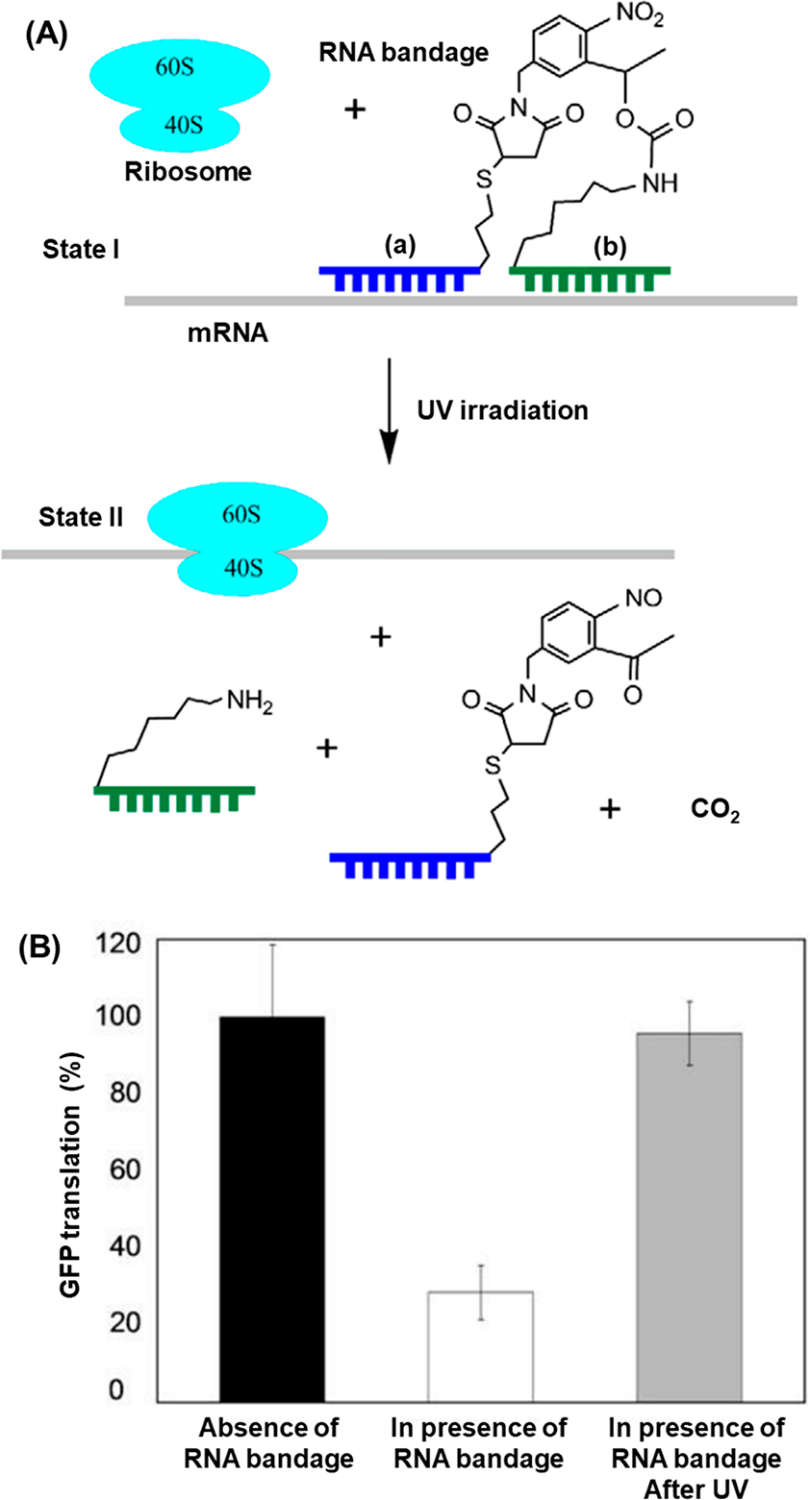
(A) Phototriggered unlocking of an ONB-protected mRNA template toward the ribosome translation of the protein on the deprotected template. (B) GFP yields in the presence of (I) the naked mRNA template, (II) the ONB-protected template in the absence of auxiliary light-induced deprotection, (III) the light-induced deprotected template. Figure adapted with permission from ref. (203). Copyright 2008, Elsevier.

Figure 17 demonstrates the photoregulation of *in vitro* protein synthesis by the design of “RNA bandages” composed of two short antisense domains linked by a photoresponsive ONB moiety.^203^ The bandages were engineered to hybridize with the 5′-untranslated region of the target mRNA. In the intact state, domains (a) and (b) form a stable duplex with the target mRNA sequence and block translation by the ribosome, Figure 17A, State I. Photoirradiation to cleave the linking ONB moiety generates two shorter fragments that separate from the mRNA target, resulting in the ribosome-induced translation (State II). Indeed, optimized lengths of bandage units reduced translation of a green fluorescent protein (GFP) miRNA by 70% in rabbit reticulocyte lysate when administered in a 10-fold excess to the target miRNA sequence, Figure 17B. After UV irradiation that cleaved the photoresponsive linker, transcription of the gene was reactivated to 95% of the level observed in the absence of the RNA blockage units.

**Figure 18:**
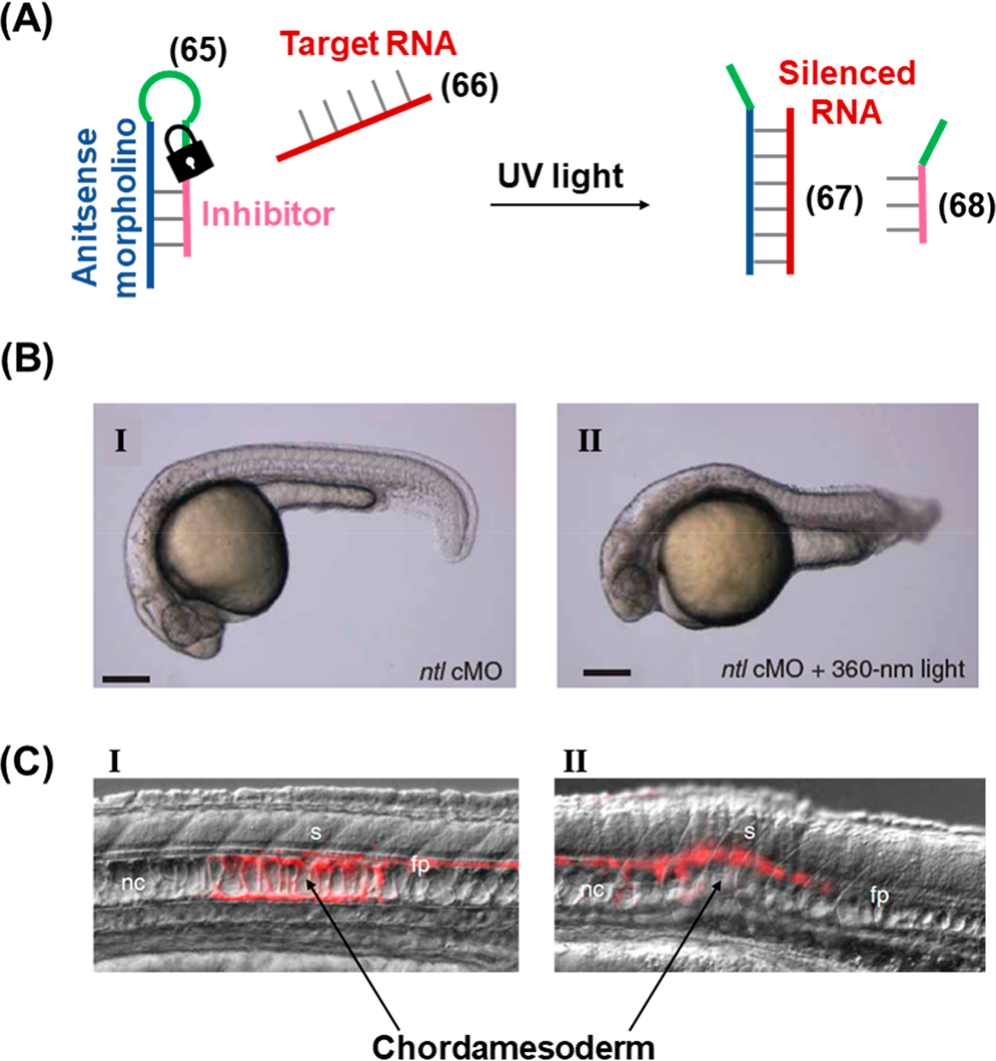
(A) Schematic application of an ONB-caged antisense morpholino hairpin for the light-induced silencing of a target RNA. (B) *In vivo* application of the ONB-protected hairpin for the phototriggered silencing of *ntl* RNA in zebrafish. Panel I: zebrafish treated with *ntl*-targeting caged morpholino (*ntl* cMO) hairpin (**65**) in the absence of light (tail developed normally), Panel II: zebrafish treated with *ntl* cMO hairpin (**65**) and subjected to photoirradiation (tail development inhibited). (C) Spatially localized activation of the RNA-silencing hairpin in zebrafish chordamesoderm domains. Panel (I): in the absence of hairpin (**65**) normal development is observed in the photoirradiated (red) region. Panel (II): in the presence of hairpin (**65**) cell patterning defects are observed specifically in the photoirradiated (red) region. Figure adapted with permission from ref. (268). Copyright 2007, Springer Nature.

Morpholino oligonucleotides find numerous applications as gene knockdown agents.^266^ A strategy for the photoactivation of morpholino oligonucleotides is depicted in Figure 18 in which the gene silencing capability of the morpholino strand was inhibited through the connection of the sequence to an inhibitor through a photocleavable loop region (**65**), Figure 18A.^268^ Hybridization of the inhibitor to the antisense morpholino strand prevented the capture of the mRNA target (**66**). Thus, translational activation proceeded and resulted in downstream gene expression. Upon UV illumination, the ONB linker in the hairpin loop was cleaved allowing displacement of the inhibitor (**68**) strand by the target mRNA to form a stable duplex (**67**) with the antisense morpholino, leading to the downstream knock-down of the corresponding gene. This strategy demonstrated the ability to control organism development *in vivo* in a zebrafish model, Figure 18B. A photocaged antisense morpholino was engineered to be complementary to *ntl* mRNA (coding for a transcription factor that regulates the formation of the zebrafish tail) and injected into embryos at the one-cell stage. In the absence of photoirradiation, the antisense morpholino remained caged by the inhibitor and the organism tail developed normally (Panel I). However, embryos irradiated with 360 nm light at the sphere stage showed abnormal tail development (Panel II) corresponding to photoactivated silencing of *ntl* expression. The spatial localization of gene silencing afforded by this technique was demonstrated by irradiating a 100-μm-diameter region of the zebrafish chordamesorderm in embryos either treated or untreated with caged morpholino (**65**), Figure 18C. To demonstrate the precise site of photoirradiation in the organism, the zebrafish were engineered to express the Kaede protein which switches from green- to red-fluorescent upon exposure to UV light. In embryos that were not treated with (**65**), the precise region of photoirradiation was observable by red Kaede fluorescence and the chordamesoderm appeared to develop normally in this region (Panel I). Meanwhile, embryos treated with photoirradiation following injection of caged morpholino (**65**) demonstrated significant cell-patterning defects in the irradiated (red) region, while cells outside of this region developed normally. A further study demonstrated the power of the technique to interrogate the precise roles of the *ntl* gene in organism development with high spatiotemporal resolution.^269^

**Figure 19:**
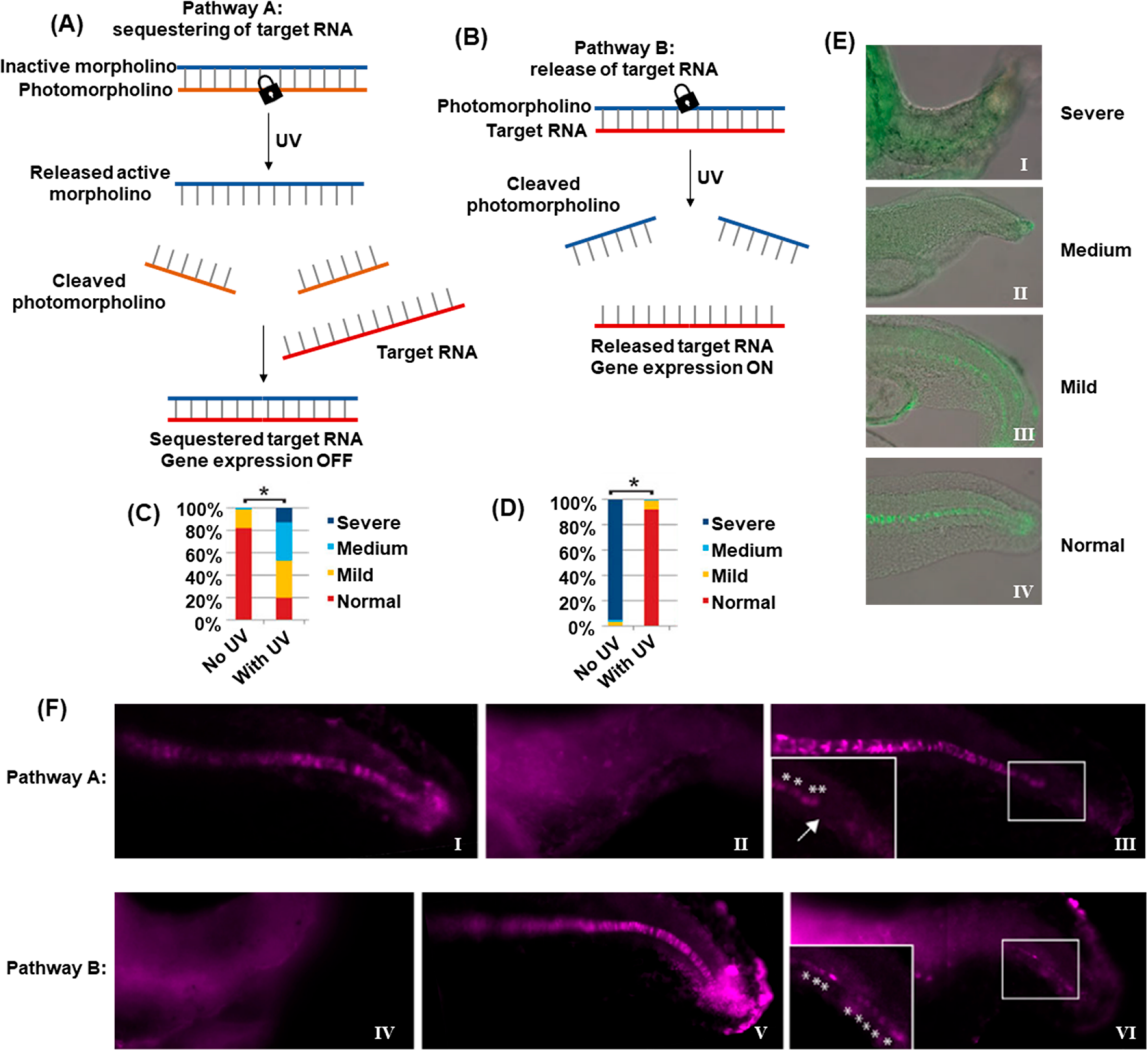
Light-induced switch-on/switch-off of gene expression using photocaged morpholino oligonucleotides. (A) Photodeprotection of the morpholino oligonucleotide that silences the target RNA and switches off gene expression. (B) Photodeprotection of sequestered target RNA activating gene expression. (C,D) Distribution of tail-development phenotypes of zebrafish embryos treated with (C) the photocaged inactive morpholino shown in (A) without exposure to light (80% normal tail development) and subjected to UV light (80% tail development perturbed), and (D) photocaged target RNA shown in (B) without exposure to light (∼100% abnormal tail development) and subjected to UV light (∼90% normal tail development). (E) Representative microscopy images of the different phenotypes in (C) and (D). (F) Spatially localized regulation of *ntla* gene expression in zebrafish tails. Panel I–III: treatment with the phototriggered switch-off construct in (A) results in normal tail development and observation of *ntla* antibody fluorescence throughout the tail in the dark (Panel I), global illumination disrupts tail development and *ntla* antibody fluorescence is not observed (Panel II) while irradiation at a specific site using a laser silences *ntla* expression in the illuminated cells (arrow shows absence of *ntla* antibody fluorescence in illuminated region). Panel IV–VI: treatment with the phototriggered switch-on module in (B) results in disrupted tail development and absence of *ntla* antibody fluorescence throughout the tail in the dark (Panel IV), global illumination restores tail development and *ntla* antibody fluorescence observed throughout the tail (Panel V) while irradiation at a specific site using a laser restores *ntla* expression specifically in the illuminated cells (asterisks show *ntla* antibody fluorescence in illuminated region). Figure adapted with permission from ref. (270). Copyright 2012, The Company of Biologists Ltd.

In addition, the full spatiotemporal switch-on and switch-off of gene expression was achieved by using two types of photocaged duplex morpholino oligonucleotides, Figure 19.^270^ The “switch off” gene expression mechanism was operated by previously described principles, in which the gene silencing capability of the antisense morpholino was inhibited by hybridization to a complementary photocleavable sense strand, Figure 19A, Pathway A. Upon photoirradiation, the antisense morpholino strand was released, sequestering the target mRNA and leading to downstream gene knockdown. For the spatiotemporal “switch on” control of gene expression, a photocleavable spacer unit was incorporated into the morpholino antisense strand, Figure 19B, Pathway B. In the initial state, the intact morpholino strand sequestered the target mRNA, switching off gene expression. Photoirradiation cleaved the antisense morpholino, leading to separation of the two resulting shorter strands and to the release of the mRNA and the reactivation of gene expression. The complementary photochemically triggered “switch on” or “switch off” mechanisms were demonstrated using a morpholino oligonucleotide engineered to silence the expression of the *ntla* gene, responsible for the regulation of development of the notochord and posterior somite tail development in zebrafish. When embryos were injected with the “switch off” photocaged morpholino, 80% developed with a normal phenotype, Figure 19C and Figure 19E (Panel IV), in the absence of photoirradiation. The remainder only suffered mild disruption in notochord development. Photocleavage of the blocking strand by irradiation of the embryos at 5 h postfertilization released the morpholino oligonucleotide and activated its gene silencing capability, evidenced by <20% embryos developing with a normal tail phenotype while the remained developed abnormal tails, approximately 50% having medium or severe disruption. In contrast, the “switch on” pathway (Pathway B) led to complete disruption of tail development in all embryos in the absence of photoirradiation, Figure 19D and Figure 19E (Panel I), while photoirradiation cleaves the silencing morpholino unit, allowing the release of the mRNA and reactivation of *ntla* gene expression, resulting in approximately 90% of the embryos developing with normal phenotype. The power of the photocleavable duplex structures to regulate gene expression with spatiotemporal precision was further demonstrated by employing a UV laser to irradiate specific cells at 11 h postfertilization, Figure 19F. Spatial resolution of *ntla* gene expression was visualized by fluorescently labeled anti-*ntla* antibodies. In the “switch off” mechanism (Pathway A), embryos developed normally in the absence of photoirradiation (Panel I). Whist broad illumination at 5 h postfertilization resulted in severe misdevelopment of the full tail (Panel II), laser irradiation of specific cells at 11 h postfertilization silenced *ntla* expression specifically in these cells, evidenced by the absence of *ntla* antibody fluorescence in the irradiated cells (Panel III, arrow indicates dark irradiated *ntla*-negative cells, asterisks highlight nonirradiated cells where *ntla* expression proceeds normally). In contrast, when treated with the “switch on” morpholino oligonucleotides (Pathway B), embryos developed with severe tail phenotypes (Panel IV) unless irradiated with UV light (Panel V). In this case, laser irradiation of specific cells with UV light specifically restored *ntla* expression in these cells (Panel VI, asterisks).

**Figure 20:**
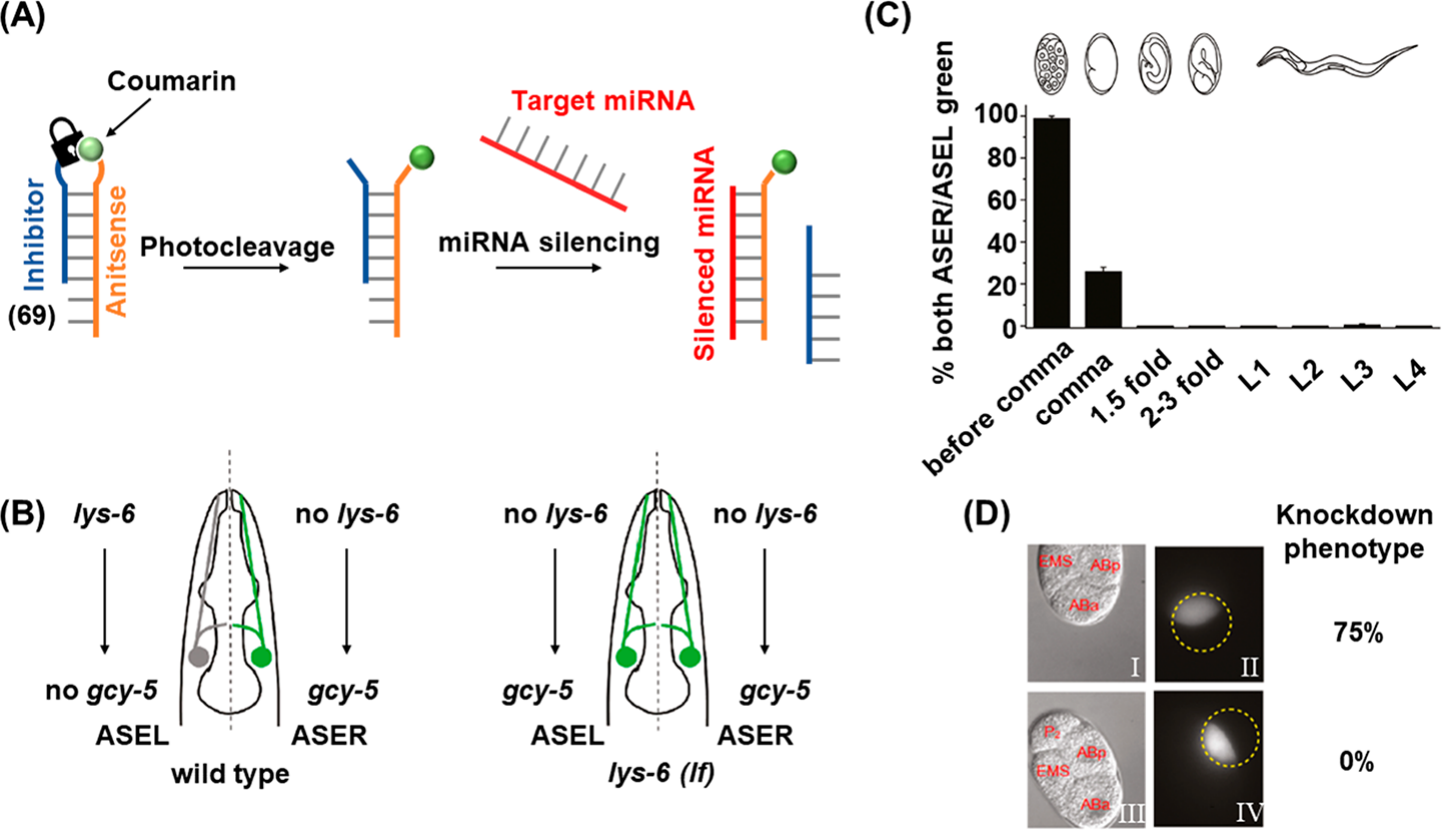
Phototriggered silencing of a target RNA by the photocleavage of a coumarin-labeled ONB-caged hairpin and displacement of the coumarin-labeled antisense strand to yield a silenced miRNA construct. (B) Schematic role of *lys-6* in controlling the expression of GFP in the ASE neurons of *C. elegans*. (C) Temporal control of *lys*-6 expression in *C. elegans* treated with the caged hairpin (**69**) and illuminated at different stages of organism development. Phototriggered knockdown of *lys-6* before the comma stage results in expression of GFP in both ASEL and ASER. Knockdown after the comma stage results in the wild-type phenotype. (D) Spatially localized activation of hairpin (**69**) in specific cells of four-cell *C. elegans* embryos. Panel I–II: specific irradiation of the ABa cell (ASEL precursor) leads to the knockdown phenotype. Panel III–IV: specific irradiation of the ABp cell (ASER precursor) results in no disruption of phenotype. The irradiation site is shown by the dotted circles and localized activation of coumarin fluorescence (corresponding to hairpin photocleavage) in the specific cells is demonstrated. Figure adapted with permission from ref. (267). Copyright 2011, American Chemical Society.

A related strategy employing photoresponsive antisense oligonucleotides to regulate miRNA activity is depicted in Figure 20.^267^ The caged antisense oligomer “cantimer” (**69**) is composed of 2′-*O*-methyl substituted oligoribonucleotides conjugated to an inhibitor within a bifunctionalized hairpin configuration engineered to contain the photocleavable ONB moiety adjacent to a coumarin fluorophore where the fluorescence of coumarin is quenched by the neighboring ONB in the intact hairpin state, Figure 20A. The fluorophore provides two functions. First, it aids hairpin photolysis by transferring energy to the nearby photolabile ONB group by FRET. Second, it allows to follow the degree of uncaging of the cantimer by monitoring restoration of fluorescence following photocleavage. The antisense component of the cantimer was engineered to block the expression of the *lys-6* protein, which was expressed in the neurons of *C. elegans* worms and is a determinant of neuronal fate specification. Figure 20B shows a simplified representation of the role of *lys-6* in controlling the expression of GFP in the ASE neurons of *C. elegans*. In the wild type, *lys-6* is only expressed in the left ASE (ASEL), which results in the downregulation of GFP expression. In the ASER, the absence of *lys-6* enables GFP expression to occur. Thus, only the ASER appears green fluorescent in normal worms. In worms containing a mutated *lys-6* gene, neither ASEL or ASER expresses the *lys-6* protein, and both domains appear green fluorescent. The utility of the photocaged probes in interrogating the spatiotemporal activity of the *lys6* miRNA during organism development was demonstrated by monitoring this phenotypic difference. Worms treated with the caged cantimers were irradiated with UV light at different developmental stages, Figure 20C. It was observed that uncaging of the cantimer (resulting in *lys-6* knockdown) before the comma stage resulted in almost all worms developing abnormally, while irradiating after the comma stage led to all organisms developing with a normal phenotype, thus demonstrating the critical role of *lys-6* in regulating organism development at this stage of the cell cycle. The spatial resolution afforded by the photoactivated probes was then shown by irradiating single cells in 4-cell *C. elegans* embryos, Figure 20D. Irradiation of the ABa cell (Panel I and II), the precursor of ASEL, resulted in 75% phenotypic disruption of the mature organism, as these cells are sensitive to the knockdown of *lys-6* (*vide supra*). Meanwhile, when an ABp cell (the precursor of ASER) was irradiated, no resulting effect on phenotype was observed, since these cells do no express *lys-6* in normal development and are therefore insensitive to the *lys-6* silencing cantimer. A related approach reported on the application of a coumarin-functionalized RNA as a photoactive agent for light-induced uncaging and gene silencing in zebrafish.^271^

### 4.4 ONB-Photoprotected Aptamers

Aptamers are single-stranded DNA or RNA oligonucleotide biopolymers revealing base-dictated three-dimensional binding interactions toward low-molecular weight substrates, biomacromolecules, and cells.^272,273^ The *in vitro* eliciting of aptamers is based on the selection and amplification of the sequence-specific binding strand from a diversified library of nucleic acids using a systematic evolution by exponential ligand enrichment (SELEX) protocol.^73,274,275^ Several strategies to exert control over the binding affinities of aptamers were demonstrated, such as the tethering of stimuli responsive chemical functionalities to the aptamer backbones. For example, tethering of methylene blue to the ATP aptamer yielded a redox-switchable aptamer revealing ON/OFF binding affinities in the presence of reducing or oxidizing agents or under electrochemical control.^276^ Not surprisingly, efforts have also been directed toward the photoregulation of aptamer activity through the use of ONB-modified nucleotides.

**Figure 21:**
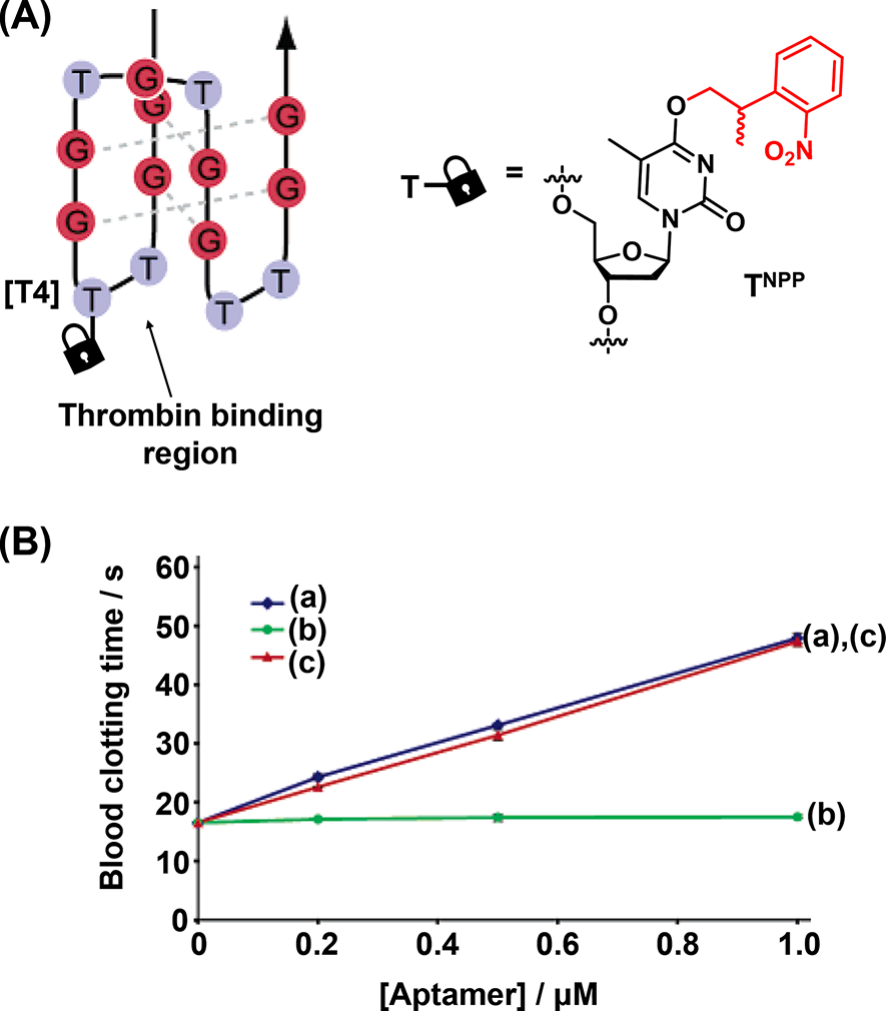
(A) An ONB-protected antithrombin aptamer generated by the photocaging of a thymine base in the thrombin binding site. (B) Concentration dependence of blood clotting times for (a) the native thrombin aptamer, (b) the inhibited ONB-caged thrombin aptamer, (c) the photodeprotected ONB-caged thrombin aptamer. Figure adapted with permission from ref. (204). Copyright 2005, American Chemical Society.

Figure 21 depicts a strategy for the photocaging the activity of the thrombin binding aptamer.^204^ The 15-base aptamer was modified to include a thymidine base caged with the 2-(*ortho*-nitrophenyl)-propyl (NPP) moiety at a strategic position, for example at T4, which is proximal to the thrombin binding region, Figure 21A. As expected, it was found that caging the thymidine residues at the binding site was most effective at inhibiting binding to the target (*K*_D_ not detectable for T4-caged aptamer), while caging a base remote from the binding site was not detrimental to the binding. Irradiation with UV light cleaved the photocaging group and restored the target binding (*K*_D_ = 139 μM) to a comparable affinity to that of the native uncaged aptamer (*K*_D_ = 99 μM). The photocaged aptamer strategy proved to be effective in regulating the activity of thrombin in the blood clotting process, Figure 21B. Addition of increasing concentrations of the uncaged aptamer slowed the blood clotting process by sequestration of the active thrombin by the aptamer, curve (a). Addition of the caged aptamer, lacking the binding affinity toward the thrombin ligand, resulted in no effect on blood clotting, curve (b), while irradiation with UV light (366 nm) restored aptamer binding activity, and the resulting sequestration of the thrombin clotting factor caused blood clotting times comparable to that in the presence of the native unprotected aptamer, curve (c).

**Figure 22:**
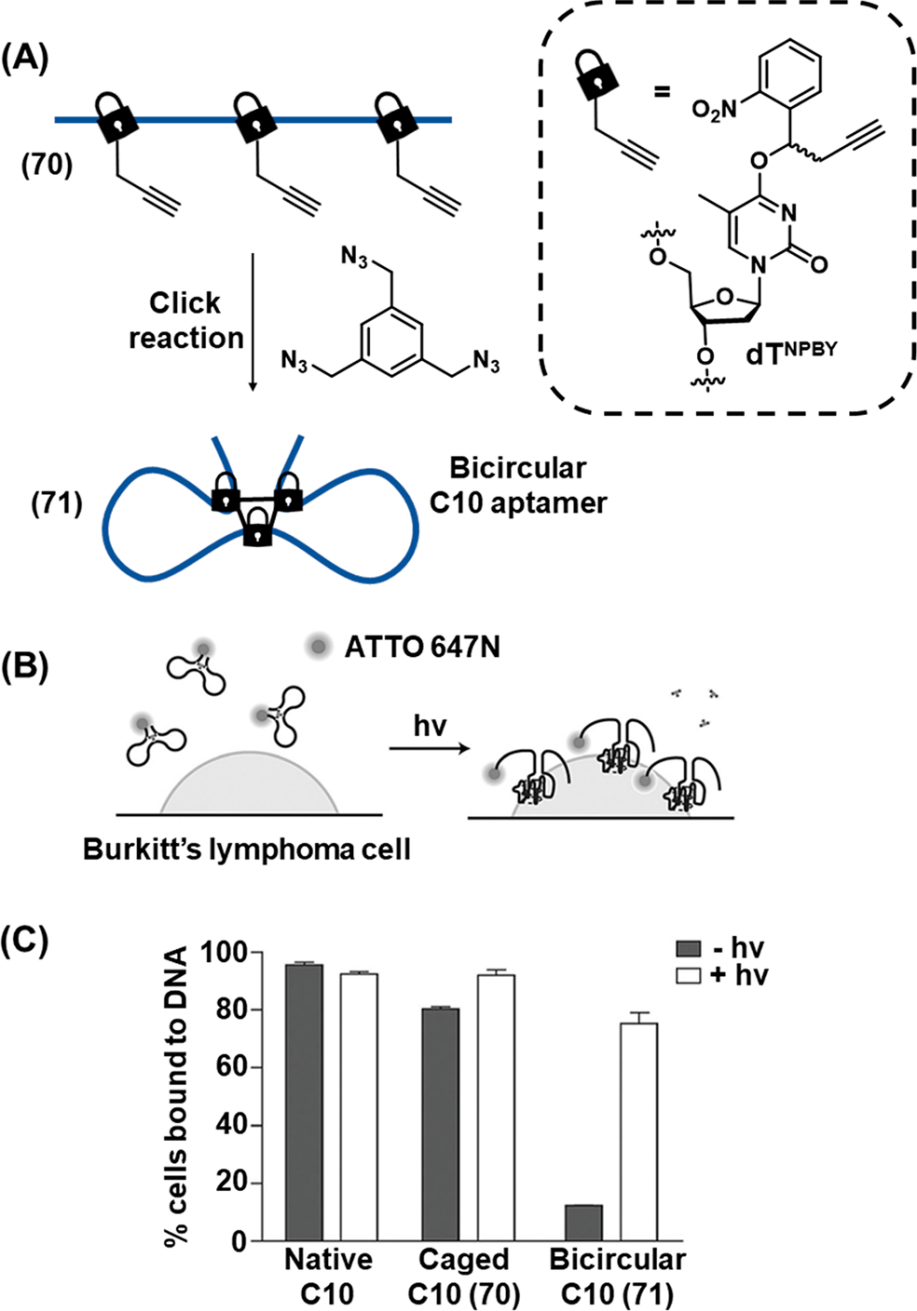
(A) Schematic bicircular caging of the ONB-acetylene functionalized C10 Burkitt’s lymphoma cell aptamer by the trimethyl azidophenyl bridging unit, applying click chemistry principles. (B) Photochemical uncaging of the ATTO 647M-labeled bicircular caged aptamer and binding of the uncaged aptamer to the lymphoma cells. (C) Binding of the native aptamer, the noncircularized ONB-modified aptamer and the bicircularized aptamer to the lymphoma cells before irradiation (black) and after irradiation (white). Figure adapted with permission from ref. (277). Copyright 2016, John Wiley and Sons.

A further method for the photocaging of aptamers applied a circularization strategy (Figure 22).^277^ A modified version of the C10 aptamer targeting Burkitt’s lymphoma cells (**70**) that included three thymine bases modified with photolabile 1-(2-nitrophenyl)but-3-yn (NPBY) moieties was prepared. The terminal alkyne functionality allowed the tethering of the photoprotected groups by a tridentate azido linker through the copper-promoted azide–alkyne click reaction to form bicyclic species (**71**), Figure 22A. This generated a bicircularized C10 aptamer in which state the binding to the target cell was inhibited. Photochemical uncaging using 365 nm UV light resulted in the cleavage of the photolabile moieties untethered the strand from its inactive bicircular structure allowing its refolding into the active secondary structure capable of recognizing the target cell, Figure 22B. By labeling the aptamer strand with a fluorophore (ATTO 647N), the binding of the aptamer to the target cells was observed by flow cytometry, Figure 22C. Notably, simple photocaging of the thymine bases without subsequent circularization by the click reaction generated an aptamer (**70**) that was active both before and after photoirradiation with comparable activity to the native C10 aptamer. Upon using the click chemistry approach to generate the bicircularized strand (**71**), aptamer binding to the target cells was dramatically reduced. Upon photoirradiation to generate the deprotected strand, binding activity was restored to approximately 80% of the level observed for the native uncaged C10 aptamer.

**Figure 23:**
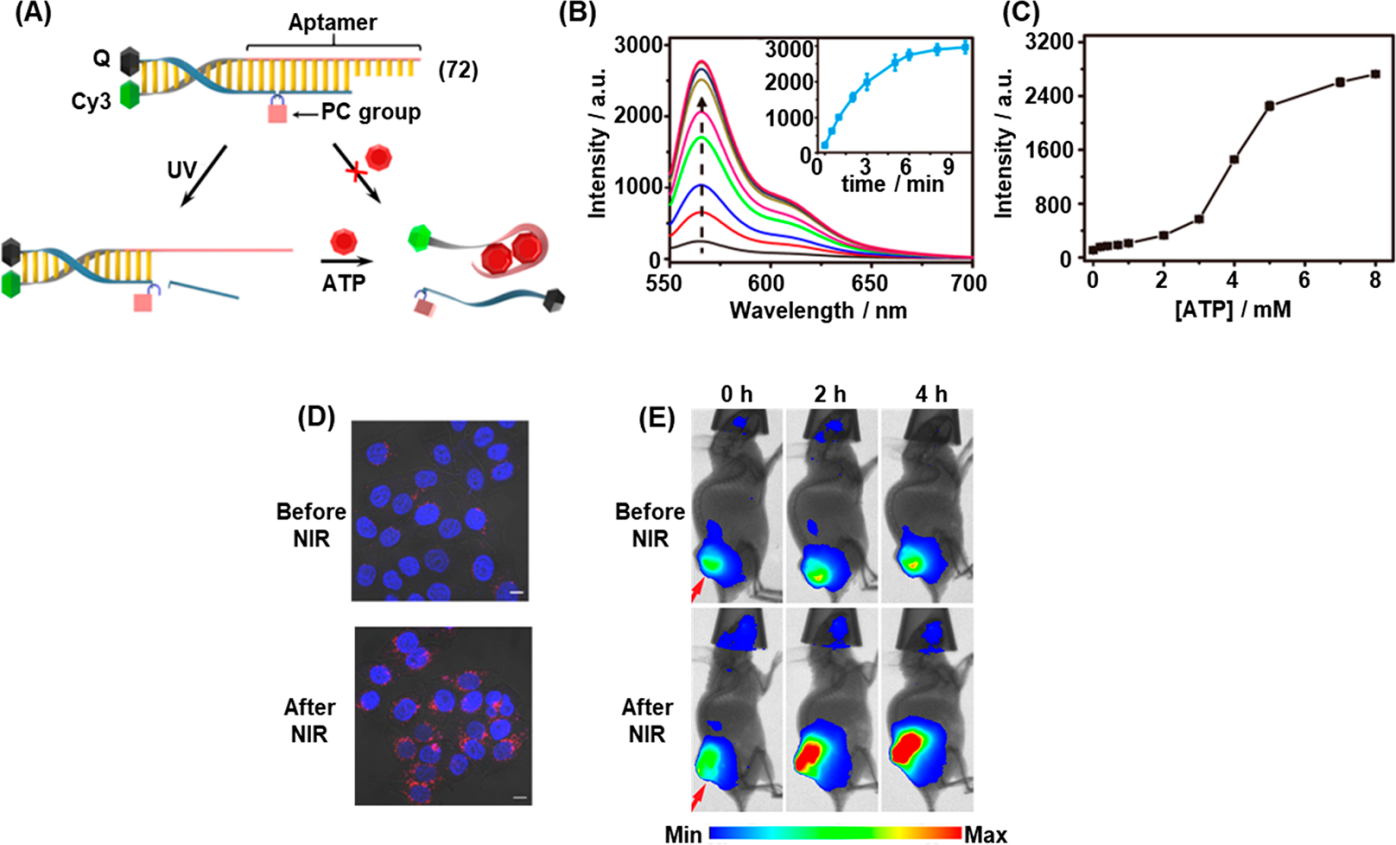
(A) Schematic application of a fluorophore/quencher ONB-protected duplex DNA that includes a caged ATP-aptamer sequence as a functional module for optical detection of ATP. The photocleavage of the ONB units lead to an unstable duplex structure being a displaced by ATP to yield the fluorescent ATP-aptamer complex as optical readout. (B) Fluorescence intensities generated by the detection module shown in (A) upon sensing ATP, 5 mM, and photochemical uncaging the photoprotective unit by irradiation for different time intervals (from 0–9 min). Inset: derived intensity vs irradiation dose curve. (C) Fluorescence intensities generated by the module shown in (A) upon analyzing different concentrations of ATP after photodeprotection of the sensing module for a fixed time interval. (D) Intracellular imaging of ATP in cells by the introduction of the sensing module and UCNPs into the cells and near-IR (NIR) activation of the module. (E) *In vivo* imaging of ATP in HeLa tumors in mice using UCNPs and the sensing module shown upon NIR activation of the detection platform. Figure adapted with permission from ref. (278). Copyright 2017, American Chemical Society.

Photocleavable DNA duplexes have also been deployed to regulate aptamer activity for intracellular and *in vivo* sensing of bioanalytes such as ATP.^278^ A DNA duplex (**72**) was engineered to include the ATP aptamer sequence in one strand, Figure 23A. However, the binding activity of the aptamer was caged through hybridization with a second strand that was engineered to contain a photocleavable linkers spaced 10 bases apart. Irradiation with UV light triggered the cleavage of the photocleavable groups and dissociation of the resulting short 10-base duplex fragments resulted in the unmasking of the aptamer binding activity. The restoration of the free aptamer was probed by installing fluorescence donor (Cy3) and quencher moieties on the aptamer and blocking strands, respectively. In the hybridized state, close proximity between the dyes led to quenching of Cy3 emission while successful binding to ATP leads to dissociation of the blocking strand and restoration of Cy3 fluorescence. Upon irradiating the system with UV light, cleavage of the blocking strand proceeded and restored ATP-binding activity, resulting in turn-on of fluorescence, Figure 23B, with maximum intensity achieved after 9 min of irradiation. Figure 23C demonstrates the fluorescence response of the system was proportional to ATP concentration. The utility of the system was further developed by loading the photoresponsive ATP probe onto NaGdF_4_:70%Yb,1%Tm@NaGdF_4_ UCNPs to allow effective photoactivation of the system using near-IR irradiation. Functionalization of the UCNPs with positively charged poly(d-lysine) allowed the loading of the negatively charged DNA sensing module through electrostatic interactions. Internalization of the photocaged UCNP/probe conjugates into HeLa cells reveled no fluorescent signal in the absence of near IR irradiation, while irradiation with near-IR light uncaged the activity of the aptamer and the intracellular Cy3 fluorescence was observed Figure 23D. Due to the effective tissue penetration of near-IR light, the system allowed the detection of ATP in an *in vivo* mouse model, Figure 23E. A similar principle was employed to detect ATP in the mitochondria of living cells with high spatiotemporal resolution,^279^ by encapsulating the photoresponsive DNA probe module into liposome-like dequalinium chloride vesicles which allow effective delivery of the probe to this organelle by virtue of their high positive charge.

### 4.5 Photodeprotection of ONB-Modified DNAzymes

DNAzymes are catalytically active oligonucleotides.^79,80^ Among the DNAzymes, sequence-specific strands promote the site-specific cleavage of RNA or DNA substrates, often in the presence of metal ions^280,281^ or amino acids (e.g., histidine)^282^ as cofactors. DNAzymes have demonstrated a number of applications in sensing technology,^283−285^ as signal transducers in DNA-based networks,^286,287^ the design of nucleic acid-based machineries,^288−290^ and as tools to regulate biochemical processes.^291,292^ The spatiotemporal control of DNAzyme activity is an attractive goal and, not surprisingly, strategies for the photocaging of DNAzyme activities have been actively pursued.

**Figure 24:**
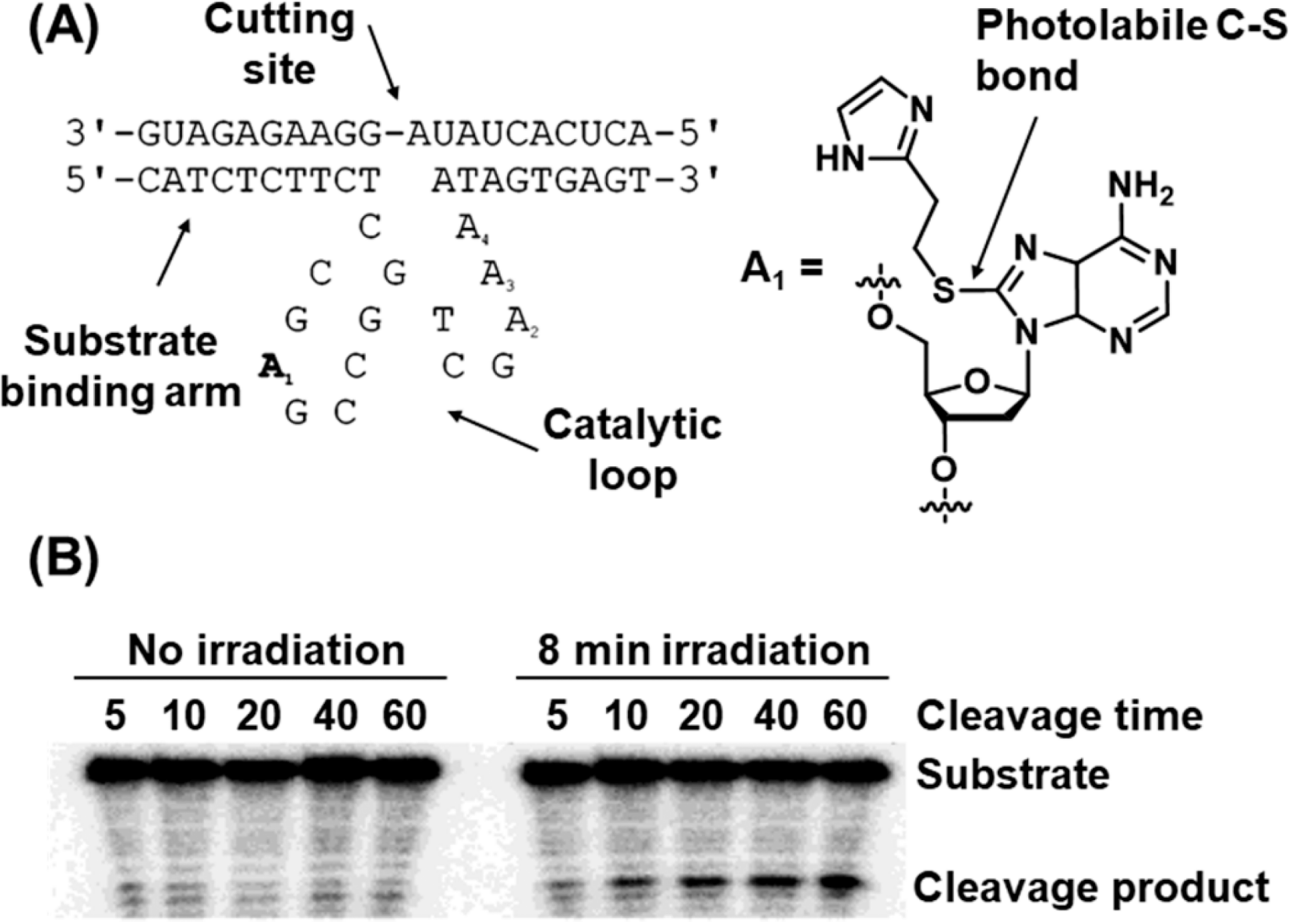
Schematic photocleavage of a thiolated adenonsine-protected Zn^2+^-ion-dependent DNAzyme/substrate complex leading to the activation of the DNAzyme activity. (B) Electrophoretic imaging of the DNAzyme activity before and after photoirradiation. Figure adapted with permission from ref. (293). Copyright 2004, American Chemical Society.

Figure 24 depicts the light-induced activation of a DNAzyme.^293^ This example is noteworthy as it relies on a thioether protecting group strategy rather than employing the ONB moiety more often employed in later studies. The activity of the DNAzyme was caged through incorporation of the photolabile protecting group on an adenosine base in the 8-17E Zn^2+^ dependent DNAzyme stem loop region, Figure 24A. Of the four possible adenosine bases for modification, only A1 (bold) resulted in complete inactivity of the DNAzyme for cleaving its RNA substrate. Gel electrophoretic experiments demonstrated that prior to photoirradiation, negligible RNA cleavage was observed even after 60 min of exposure to the inactivated DNAzyme, Figure 24B. Upon photolysis of the carbon–sulfur bond (254 or 283 nm for 8 min), the DNAzyme activity was restored leading to effective RNA cleavage. The limitation of the system is, however, the short wavelength required for photolysis, and later efforts developing photocontrolable DNAzymes made use of the ONB group, as it allows photoactivation at longer wavelengths that are more compatible with DNA-based applications.

**Figure 25:**
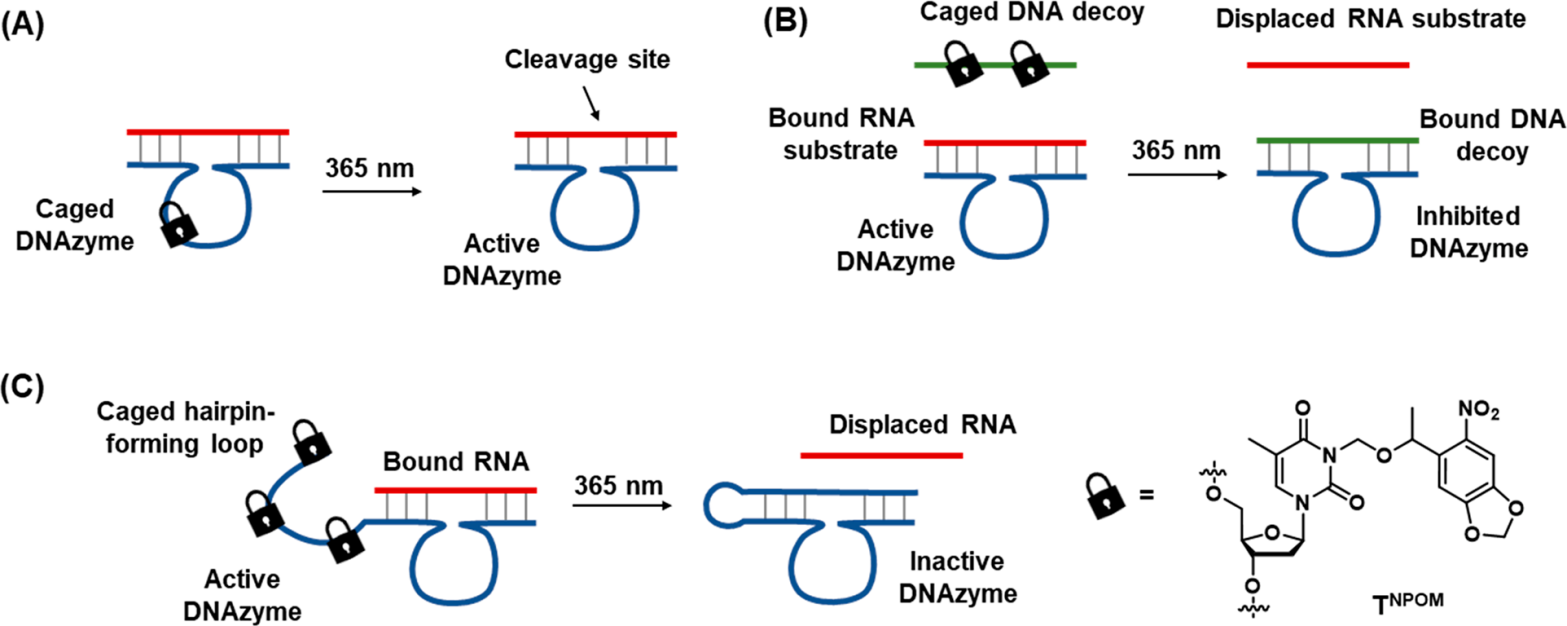
Schematic strategies to photochemically control DNAzyme activities of ONB-protected nucleic acid constructs. (A) Photodeprotection of an ONB-functionalized loop domain of a Mg^2+^-dependent DNAzyme leading to activation of catalytic activity. (B) Photodeprotection of an ONB-modified strand inducing the release of an inhibitor strand displacing the substrate strand associated with an active Mg^2+^-ion dependent DNAzyme/substrate complex. (C) An ONB-functionalized tether conjugated to an active Mg^2+^-ion dependent DNAzyme/substrate construct being deactivated by photodeprotection of the ONB-modified tether and the displacement of the substrate constituent by hairpin formation.

The ONB-derived 6-nitropiperonyloxymethyl (NPOM) group proved effective as a photocaging group for thymidine, allowing the development of several different strategies for the photoregulation of DNAzyme activities (Figure 25).^205^ Incorporation of the NPOM protecting group at the inside the catalytic loop of the 10–23 Mg^2+^-dependent DNAzyme deactivated the catalytic function, Figure 25A. Activity was restored upon 365 nm induced cleavage of the protecting group, followed by a secondary annealing process required to eliminate structural perturbation of the strand induced by the caging moiety. Photocaged DNA decoys were also used to regulate the activity of the DNAzyme, Figure 25B. In the caged state, the decoy was unable to hybridize with the substrate binding arms of the DNAzyme and RNA cleavage proceeded normally. By decaging the NPOM-protected thymine moieties with 365 nm light, the DNA decoy outcompeted the RNA substrate for binding to the DNAzyme and DNAzyme activity was switched off. Finally, elongation of the substrate binding arms with a photocaged strand engineered to be complementary to part of the DNAzyme sequence led to an active DNAzyme in the rest state as hairpin formation was inbibited by the caging moieties, Figure 25C. Removal of the NPOM groups with UV light triggered the formation of the hairpin structure which prevented the binding of the RNA substrate and switched off DNAzyme activity.

**Figure 26:**
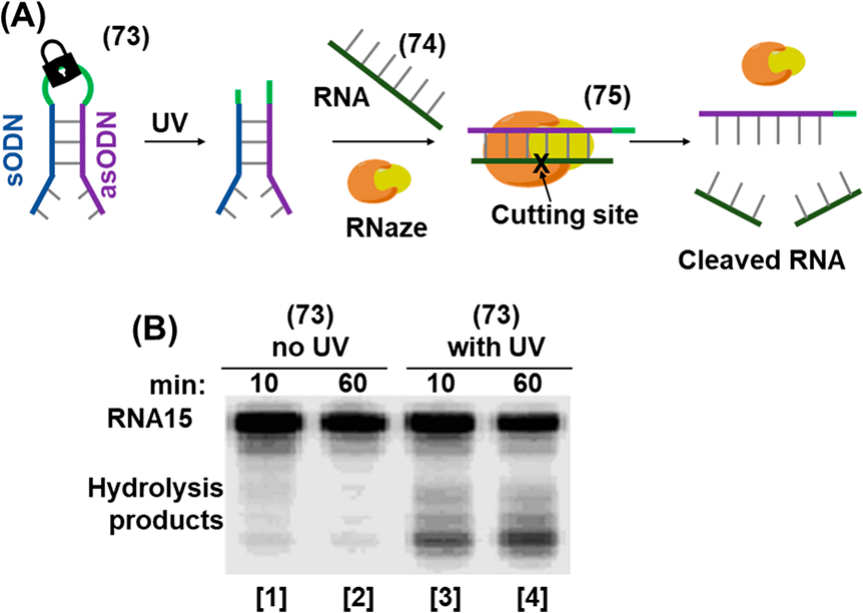
(A) Schematic application of an ONB-protected hairpin structure being photodeprotected to yield a duplex unit that is displaced by an RNA strand to yield a functional duplex for the guided cleavage of RNA by RNase. (B) Electrophoretic imaging of the cleavage of the target RNA by the photodeprotection of hairpin (**73**). Figure adapted with permission from ref. (294). Copyright 2006, John Wiley and Sons.

Photoregulation of the activity of RNase, an RNA digestion catalyst, was achieved by the engineering of a photoresponsive DNA hairpin (Figure 26).^294^ Hairpin (**73**) was designed to contain an antisense oligodeoxynucleotide (asODN) sequence linked to the complementary sense oligodeoxynucleotide (sODN) by a photocleavable ONB-containing loop, Figure 26A. The intact form of the hairpin duplex demonstrated a high melting temperature (*T*_m_ = 80 °C) and was inert to hybridization with the target RNA strand (**74**). Upon UV light irradiation, cleavage of the photoresponsive linker significantly reduced the duplex stability (*T*_m_ = 51 °C), allowing the toehold-mediated displacement of sODN by RNA (**74**) to form the asODN/RNA duplex (**75**). In this duplex state, the RNA was reactive toward RNase and was cleaved by the enzyme into two short fragments. The effective control of the enzymatic reaction by photoirradiation was demonstrated by gel electrophoresis, Figure 26B. When the asODN was blocked by linking the complementary sODN nucleotide through the photocleavable hairpin loop, only trace quantities of cleavage products (4.6%) were detected after 60 min in the absence of photoirradiation (Lane 2), demonstrating the inhibition of RNA/asODN duplex formation by the tethered sODN strand. However, activating the asON/sON hybrid toward strand displacement by cleavage of the hairpin loop with UV irradiation, the cutting activity was restored and 45% cleavage of the target RNA was observed after 1 h (Lane 4).

### 4.6 ONB-Functionalized Nucleic Acids for Logic Gate Operations and DNA Computing

The precise and programmable nature of nucleic acids has enabled these biopolymers to function as the basis of molecular logic gates, in which preprogrammed combinations of multiple inputs leads to transduction events based on strand hybridization in order to generate an output event, such as a fluorescent signal or the release of a load from an encapsulated structure.^295,296^ This has enabled the design of synthetic DNA-based computational circuitries that may be interfaced with biological machinery toward a variety of applications including the targeted delivery of payloads to cells,^297^ pH sensing^298^ and miRNA detection.^299^ The versatility of the DNA nanotechnology toolbox has enabled DNA logic gate operations using a variety of inputs including nucleic acid strands,^300^ or the binding of substrates to aptamer sequences.^301^ Not surprisingly, the engineering of photoresponsive ONB-containing DNA strands has also been exploited in the design of such systems, where light either uncages the reactivity of the strands to stimulate the logic gate operation, allowing spatiotemporal activation of the DNA computational event, or acts itself as one of the input-stimuli of the DNA-based logic gate module.

**Figure 27:**
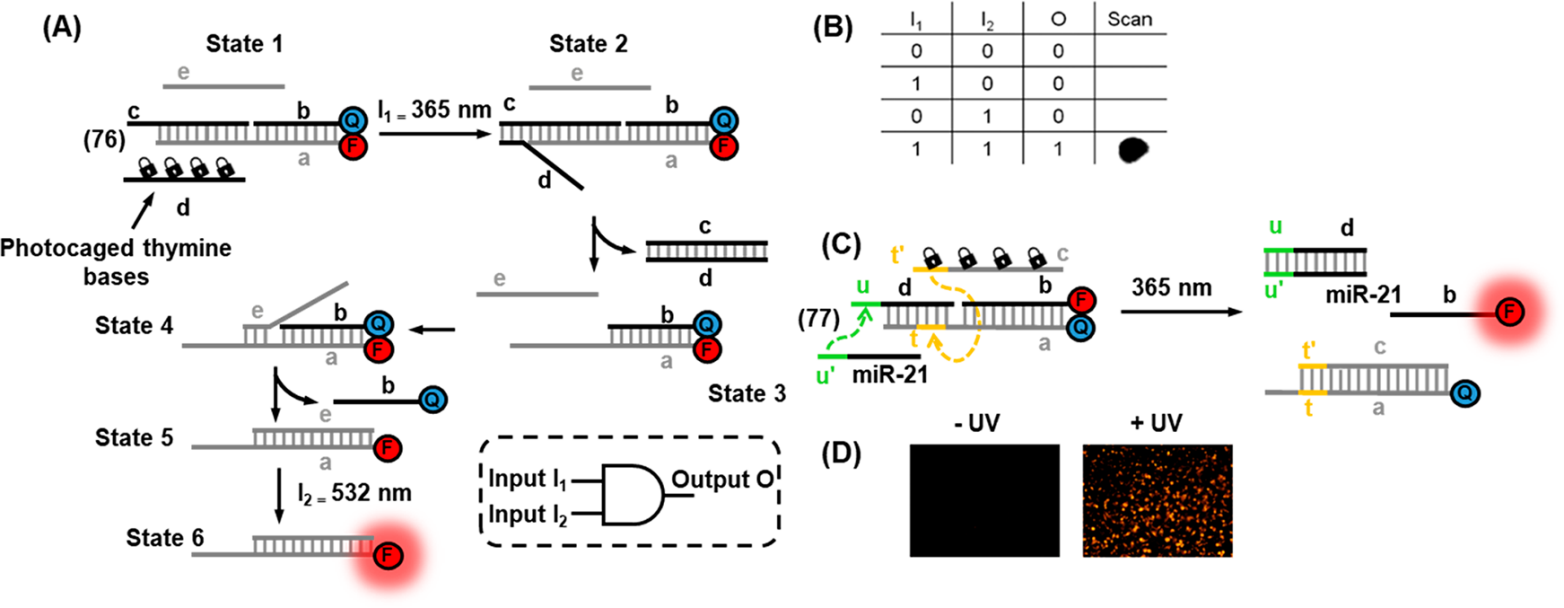
(A) Schematic reaction module executing an AND logic gate operation using two different wavelengths of light as inputs. (B) Truth table corresponding to the AND logic gate present in (A) immobilized in an agarose gel, with different regions illuminated with different combinations of I_1_ and I_2_. (C) Schematic reaction module using light and miRNA-21 as inputs for the intracellular sensing of miRNA-21 guided by the AND gate operation. (D) Application of the reaction module shown in (C) to trigger the logic gate optical transduction corresponding to the sensing of miRNA-21 in HeLa cells. Panel (A), (B) adapted with permission from ref. (302). Copyright 2012, American Chemical Society. Panel (C), (D) adapted with permission from ref. (303). Copyright 2013, American Chemical Society.

Photocaged single-stranded DNA was directed toward the construction of DNA logic gates (Figure 27).^302^ The principle of the system is demonstrated in Figure 27A. The computing module consisted of a duplex (**76**) that consisted of a fluorophore-modified strand (a) hybridized to both a quencher-functionalized strand (b) and second blocking strand (c). The proximity of the dyes led to fluorescence quenching in the initial state (State 1). Strand (d) and (e) were included in the computing module as auxiliary triggers. Strand (d) was engineered to displace blocking strand (c) through toehold-mediated strand displacement. However, this process was inhibited by the modification of thymine bases in (d) with photolabile ONB caging groups, preventing strand hybridization. Upon photocleavage of the caging moieties with UV light acting as one input (*I*_1_, λ = 365 nm), displacement of blocking strand (c) by the uncaged (d) strand proceeded through toehold mediated strand displacement (State 2), to form the (c)/(d) duplex (State 3). The displacement of strand (c) unmasked a toehold region on strand (a), allowing toehold-mediated strand displacement of the quencher strand by the second uncaged (e) strand (State 4), to form the (a)/(e) duplex (State 5). Since this duplex no longer contained the quenching moiety, the fluorescence was activated and acted as the output signal in response to the two inputs (*I*_1_, λ = 365 nm and *I*_2_, λ = 532 nm, State 6). It should be noted that prior to the displacement of (c) by the first input strand, triggered by photoirradiation, the initial duplex (**76**) was unreactive to (e) as the relevant toehold was masked by the formation of the (a)/(c) duplex. As the output signal is only observed following exposure to both I_1_ and I_2_ inputs, the system can be considered as an AND logic gate, Figure 27A, inset. The spatial resolution afforded by the photocaging strategy was also shown by immobilization of the logic gate module in a low-melt agarose gel. Figure 27B shows the truth table constructed from a set of experiments in which specific regions of the gel were subjected to different combinations of the two inputs. In the absence of either input, or in the presence of only one input, no fluorescence was observed. Subjecting the loaded gel to both inputs sequentially led to observed fluorescence. The phototriggered logic gate operation was further applied to detect cellular miRNA, demonstrating the possibility to interface synthetic photoresponsive nucleic acid logic circuitries with cellular environments, Figure 27C.^303^ Since the fluorescence signal was only generated in the presence of both photoirradiation and the miRNA inputs, duplex (**77**) acted as an AND gate module, requiring the simultaneous presence of two inputs in order to generate a positive readout. Figure 27D shows that upon transfecting the HeLa cells, where miRNA-21 was overexpressed, with the AND gate (**77**), the TAMRA fluorescence was activated only upon following photoirradiation as second input.

**Figure 28:**
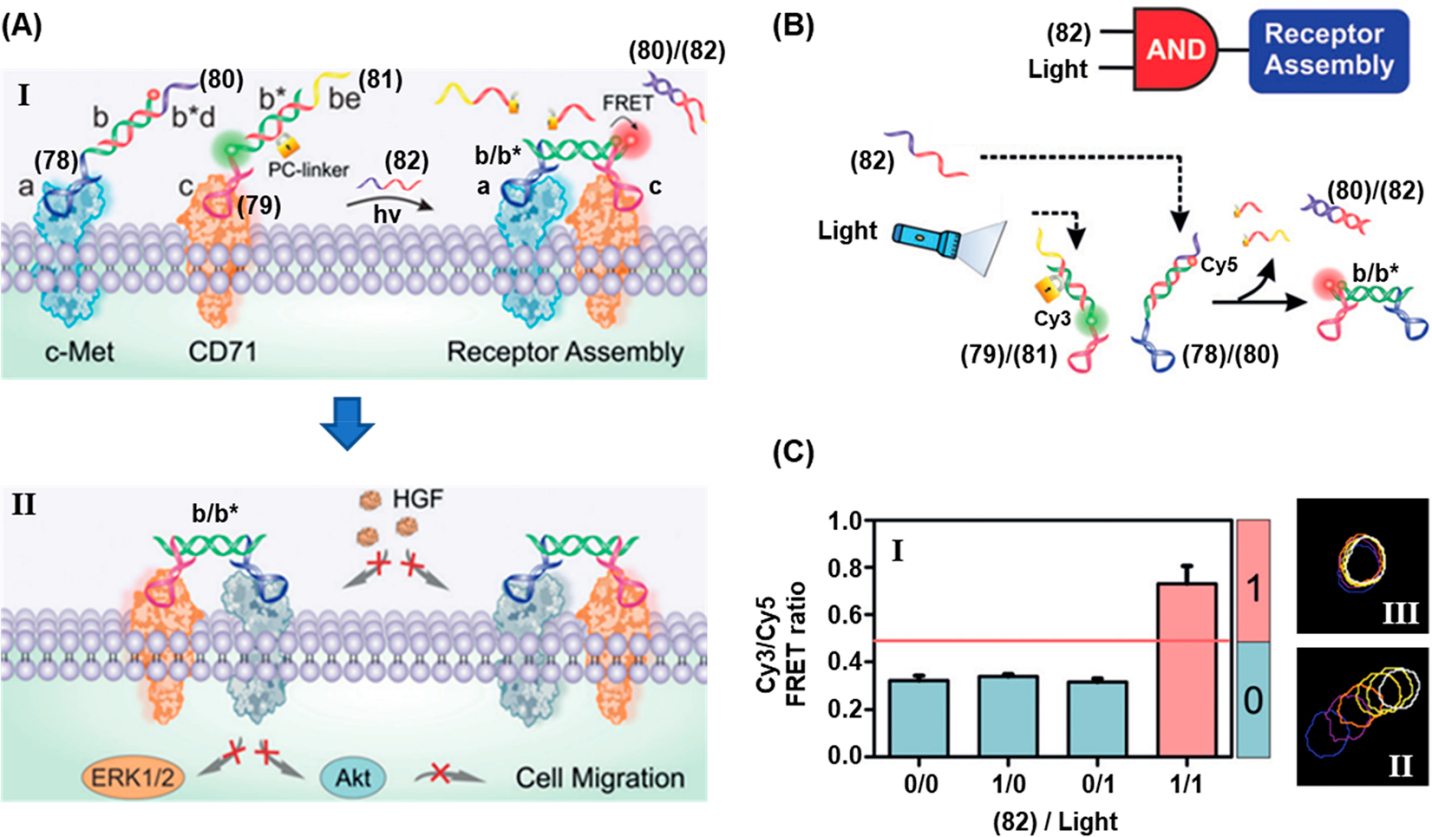
(A) Schematic probing of the c-Met and CD7 cell membrane receptor stimulated migration of cells using aptamer constructs controlling the receptor-induced migration functions by an AND gate logic operation. While the individual aptamer constructs do not affect the cell migration functionalities, the assembly of the receptors stimulated by uncaging the aptamer bridging units with simultaneous application of photochemical ONB-strand cleavage and auxiliary DNA strand inputs (Panel I) inhibits cell migration by preventing binding of HGF (Panel II). (B) Schematic activation of the aptamer assembly by the AND gate operation. By appropriate Cy3/Cy5 labeling of the aptamer constructs the dynamic bridging of the aptamers and inhibition of cell migration are probed by the resulting FRET signal output. (C) Fluorescence output signals demonstrating the AND gate induced bridging of the receptors (Panel I) and confocal microscopy imaging of cell migration demonstrating motility in the absence of both AND gate inputs (Panel II) and inhibited motility in the presence of both inputs (Panel III). Figure adapted with permission from ref. (304). Copyright 2019, John Wiley and Sons.

Photocleavable DNA duplexes were employed in the realization of DNA-based logical gates that control cell signaling transduction (Figure 28).^304^ The c-Met and CD71 cell membrane receptors control the HGF-stimulated formation of ERK 1/2 and AKT thereby regulating cell migration. Binding of HGF to c-Met stimulates the signal transduction pathway guiding cell motility, while association of c-Met and CD71 inhibits HGF binding and silences this pathway. Accordingly, two strands (**78**) and (**79**) containing aptamers recognizing the c-Met (a) and CD71 (c) receptors, were extended by complementary tethers b and b*, respectively. The tethers were caged with strand (**80**) and ONB-photoprotected strand (**81**), respectively. The caging prevented the association of the b/b* domains of (**78**) and (**79**), inhibiting receptor assembly and allowing the HGF-stimulated activation of the downstream cellular process (Panel I). Subjecting the cells to fuel strand (**82**) acting as one input displaces the toehold-bearing strand (**80**) from (**78**) through the formation of duplex (**80**)/(**82**). The photocleavage of strand (**81**) into fragments, as the second input, releases free strand (**79**). The release of free strands (**78**) and (**79**) permits the hybridization of the b/b* domains, leading to receptor assembly on the cell surface. This interaction prevents the binding of HGF to cMet and silences the downstream cellular migration pathway (Panel II). Thus, the AND-gate input combination comprising the fuel strand (**82**) and the light signal activates receptor assembly as output, Figure 28B. The input-driven gating of the neighboring receptor site was supported by labeling the strand (**78**) with the Cy5 and the strand (**79**) with the Cy3 fluorophores, respectively. While in the caged configuration the spatial separation of the fluorophores prohibited the FRET signal between the fluorophores, the AND-gate input-guided separation of the caging strands by the fuel strand (**82**) and light brought the fluorophores into spatial proximity through formation of the b/b* duplex that allowed an effective FRET signal that acts as a reporter of the assembled receptor configuration output, Figure 28C.

### 4.7 Photostimulated Fusion of ONB-Modified Interfaces

**Figure 29:**
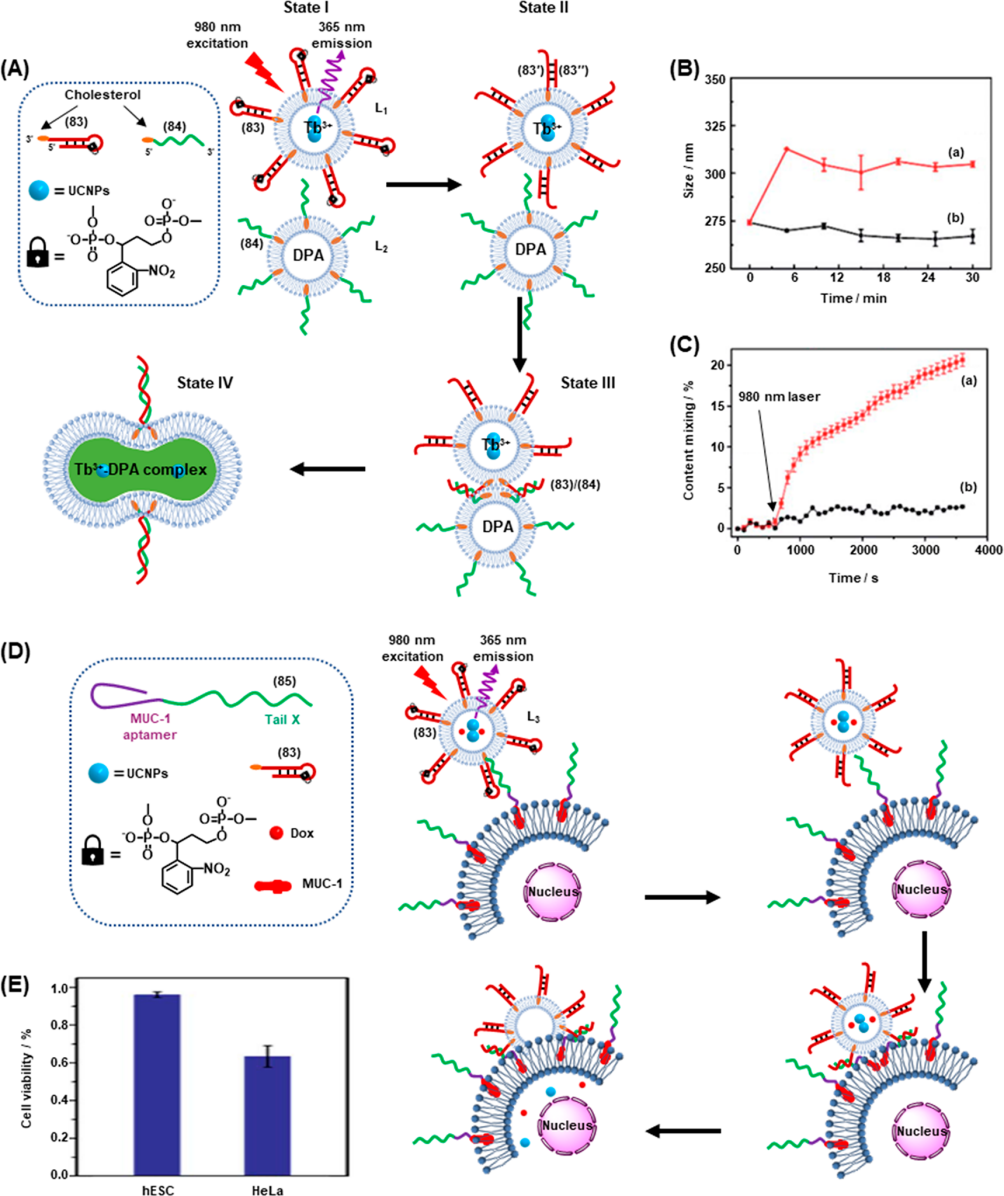
(A) Photochemical fusion of nucleic acid functionalized liposomes via the application of an ONB-hairpin-modified liposome loaded with UCNPsfor the NIR cleavage of the ONB-protected hairpins. The fusion leads to mixture of the loads in the liposomes. (B) Size increase of the fused liposomes induced by irradiation with light (curve a); no size changes of the liposomes occurs in the absence of light (curve b). (C) Time-dependent fluorescence changes corresponding to formation of the Tb^3+^/DPA complex generation upon the light-triggered fusion and the mixing of Tb^3+^ and DPA in the fused containment (curve a) triggered by NIR irradiation. No fluorescence changes are observed in the absence of light-induced fusion (curve b). (D) NIR-induced fusion of ONB-hairpin-functionalized, doxorubicin-loaded liposomes with MUC-1 aptamer functionalized HeLa cells. (E) Selective cytotoxicity toward HeLa and hESC cells demonstrated upon treatment of the cells with the UCNP/Dox-loaded ONB-protected liposomes and subsequent NIR fusion triggered by unblocking of the hairpins. Successful fusion proceeds only with the MUC1-aptamer-modified HeLa cells. Figure adapted with permission from ref. (308). Copyright 2019, Creative Commons CC-BY 3.0.

The development of artificial cell-mimicking compartments has attracted substantial research effort as a means toward the development of protocells in the rapidly developing field of “Systems Chemistry”.^305−307^ Photocleavable DNA hairpins were utilized to realize the near-IR triggered spatiotemporal activation of fusion of liposomes or the fusion of liposomes with cancer cells for the targeted release of drug loads (Figure 29).^308^ The principle of the system is shown in Figure 29A. Functionalization of photoresponsive DNA hairpin (**83**) with cholesterol at the 3′ terminus allowed the strand to be incorporated in the phospholipid membrane of liposome L_1_. L_1_ was engineered to include UCNPs, which possess an absorbance band at 980 nm and emit at shorter wavelength, 365 nm, to trigger the photocleavage of the loop region of hairpin (**83**) and was also loaded with Tb^3+^ ions. A second liposome, L_2_, was designed to contain 2,6-dipyridinecarboxylic acid (DPA) and its boundary was functionalized with the cholesterol-modified nucleic acid strand (**84**). This sequence was designed to be complementary to that of the stem of (**83**), yet hairpin (**83**) was inert to strand (**84**) due to the presence of the ONB-modified loop (State I). Upon irradiating the system with 980 nm light, UCNP-mediated photocleavage of the nitrobenzyl moieties of the (**83**) loops proceeded, exposing a single-stranded toehold region in the cleaved (**83**) strand (State II). Under these conditions, (**84**) readily displaced the (**83**) duplex through toehold-mediated strand displacement to form the more stable (**83**)/(**84**) duplex (State III). This hybridization process brought the phospholipid membranes of liposomes L_1_ and L_2_ into contact and triggered liposomal fusion (State IV), resulting in the mixing of the contents of L_1_ and L_2_. The fusion process was evidenced by the increase in the size of the resulting liposomes generated upon the fusion process, Figure 29B. While light-scattering experiments demonstrated that the NIR-irradiated liposomes reveal an increase in diameter from 260 to 310 nm, curve (a), the nonirradiated liposomes did not show any size changes upon mixing of L_1_ and L_2_. In addition, the NIR irradiation (980 nm) of the mixture of liposomes L_1_ and L_2_ led to a time-dependent fluorescence change upon fusion of the liposomes, Figure 29C. The time-dependent fluorescence increase, curve (a), originated from the fusion of the liposomes, exchange of loads, and the formation of the fluorescent Tb^3+^-DPA complex in the fused containment. As before, the mixture of nonirradiated liposomes did not lead to any temporal fluorescence changes, Figure 29C, curve (b), since the fusion process was inhibited and the exchange of loads was prohibited.

The phototriggered cleavage event involving ONB nucleic acid functionalized liposomes was further utilized to deliver a drug payload to cancer cells, Figure 29D. A nucleic acid strand (**85**) consisting of an aptamer sequence against the mucin 1 protein (MUC-1) at one end, and a tail sequence (X) complementary to the stem region of the caged hairpin (**83**) functionalized liposome L_3_ was engineered. The liposomes were loaded with the anticancer drug doxorubicin (Dox) and with the UNCPs required to mediate the near-IR activated photocleavage process. As MUC-1 is overexpressed on the surface of the HeLa cells, strand (**85**) was specifically captured by these cells by formation of the MUC-1 complex with its aptamer, while cells that do not express MUC-1 (in this case normal hESC cells) were left unfunctionalized with strand (**85**). Thus, upon photocleavage of the hairpin (**83**) units on liposome L_3_ with NIR light, liposome L_3_ specifically associated through hybridization with strands (**85**) associated with the HeLa cells by formation of the bridging duplex. Fusion of the liposome with the target cell released Dox into the cell, leading to cytotoxicity. Cell viability experiments confirmed the specific targeting of HeLa cells with the drug payload, Figure 29E. After 2 days, the nontargeted hESC cells remained fully viable, while HeLa cells demonstrated a viability of 65%. Control experiments demonstrated that no toxicity to either cell line was observed in the absence of NIR-light activation, indicating the critical function of the photoresponsive element allowing the drug delivery through the spatiotemporal control over the liposome fusion with the cancer cells.

### 4.8 Photopatterning of ONB-Modified Nucleic-Acid Functionalized Surfaces

The development of strategies for the organization of nanoscale components and biomolecules onto surfaces attracts significant research attention.^309−313^ The spatially dictated functionalization of surfaces has demonstrated broad applicability in a range of applications including in the development of electronic devices^314−316^ and sensors,^317,318^ and the control of biological processes such as cell adhesion and migration.^319−321^ In particular, the patterning of surfaces with DNA offers a range of opportunities based on the versatile structural and functional properties of nucleic acids, such as the ability to design molecular or cellular recognition features into DNA strands (aptamers), or engineering catalytic functions, self-assembly properties, or gene expression machineries into the DNA structures. Moreover, the conjugation of DNA strands to auxiliary functional or reporter units such as enzymes or fluorescent nanoparticles allows the DNA-guided positioning of these elements on the functionalized patterned surface. A variety of methods for patterning of surfaces with DNA have been deployed, including soft lithographic techniques,^322,323^ dip-pen nanolithography^324,325^ and inkjet printing.^326,327^ Photolithography^328^ and electron beam lithography^329^ have also allowed the patterning of surfaces with DNA structures. By applying these methods, surfaces are coated with an appropriate photoresist which may be patterned with light or an electron beam to generate spatially dictated reactive regions on the surfaces that are subsequently functionalized with the desired DNA structures. The possibility to assemble photocleavable nucleic acid strands on surfaces allows a complementary light-stimulated approach for surface patterning of DNA. By this approach, the initial homogeneous surface deposition of the photocleavable DNA strands allows the subsequent spatially dictated formation of reactive surface regions by the site-specific photocleavage of the strands (through a mask or with a scanning laser) to generate reactive toehold regions that may be used to initiate site-specific functionalization of the surface through further DNA hybridization reactions. This approach has been successfully deployed for multicolor photopatterning of surfaces with fluorophores,^330−332^ the spatially guided immobilization of gene expression machineries on surfaces,^331^ the site-specific capture of target molecules and nanoparticles,^333^ and also for the lithographic patterning of 3D microparticle surfaces.^334^ The following sections introduce the application of photocleavable DNA strands toward these applications.

**Figure 30:**
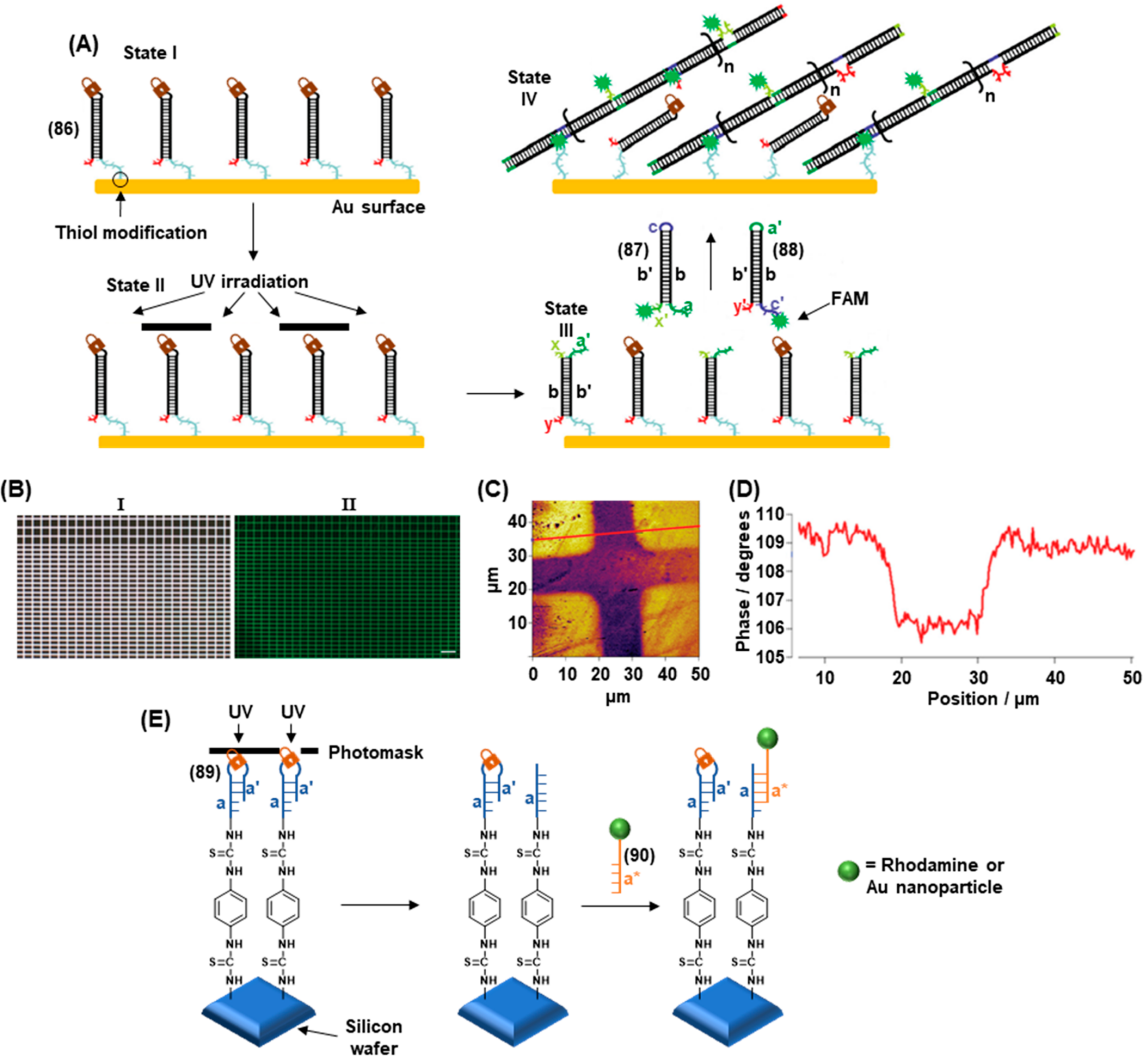
(A) Schematic photolithographic patterning of an ONB-protected hairpin monolayer-modified surface by selective UV light induced deprotection of the monolayer through a mask and the subsequent activation of the HCR in the presence of fluorescein-modified hairpins (**87**)/(**88**). The selective formation of fluorophore patterned domains of oligomeric DNA structures is imaged by fluorescence confocal microscopy and AFM. (B) Fluorescent image of the DNA-patterned surface generated upon photolithographic patterning of the monolayer using a grid-like mask. (C) AFM image of the square corner edges of the patterned DNA interface. (D) Cross-sectional profile of the patterned surface. (E) Schematic photopatterning of an ONB-protected monolayer-modified surface through a mask and selective deposition of nucleic acid-modified fluorophores or nanoparticles to the deprotected domains through complementary base-pair hybridization. Panel (A)–(D) adapted with permission from ref. (332). Copyright 2015, John Wiley and Sons.

The photoregulated HCR was exploited to allow the patterning of DNA polymer brushes onto a gold-coated glass surface (Figure 30).^332^ The initiator thiolated hairpins (**86**) that include in their loop domain an ONB photoprotective unit were assembled as a monolayer on a gold surface (State I). The DNA-functionalized surface was irradiated with UV light through a photomask designed to display a particular pattern (State II). Only unmasked regions of the surface were exposed to the UV light, leading to spatially dictated cleavage of the photolabile hairpin (**86**) loops that generated the sticky ends (x and a′) that initiate the subsequent HCR (State III). Hairpins positioned in masked regions of the surface were shielded from UV irradiation and remain intact and inert to the HCR process. Subjecting the photopatterned surface to the two hairpins (**87**) and (**88**) initiated at the activated sites the HCR process, resulting in the formation of DNA polymer brushes at dictated regions of the gold surface (State IV). By labeling hairpins (**87**) and (**88**) with the FAM fluorophore, the surface patterning was observed by fluorescence microscopy following the photocontrolled HCR. The precision of the technique was demonstrated by irradiation of the DNA-functionalized surface through a photomask containing a grid of 10 μm lines, Figure 30B, Panel I. Panel II shows the fluorescence image of the surface following photoirradiation and HCR, demonstrating that the line widths of the polymer brushes generated after photopatterning are comparable in size to the dimensions of the photomask. The presence of the polymer brushes on the surface was directly visualized by atomic force microscopy. Figure 30C shows the AFM phase image of the desired cross-shaped pattern. The well-resolved the cross-sectional profile of the surface is depicted in Figure 30D. The same principles were applied to generate multicolored arrays, through incorporation of different dyes on hairpins (**87**) and (**88**). Surface patterning of polymer brushes with molecular recognition capabilities was also achieved by labeling the respective hairpins with biotin for specific binding of streptavidin-conjugated substrates.

An additional ONB-modified hairpin-mediated photolithographic system is depicted in Figure 30E.^333^ An amine-terminated silicon wafer was functionalized with self-complementary DNA hairpins (**89**) using a bisthiourea linker. The hairpins were covalently attached to the surface through their 3′ ends and comprised of a self-complementary duplex region (a/a′) interconnected by a loop engineered to contain a photolabile ONB linker. Photochemical cleavage of the protective units through a mask (λ = 340 nm) separated the hairpin loops and after washing of the a′ strand the remaining single-stranded sequence (a) remained covalently attached to the silicon surface. The pattern of the single stranded nucleic acid strand generated by the photocleavage of the interface through the mask was, then, imaged by hybridization of a fluorophore (rhodamine)-modified strand (**90**) or an Au nanoparticle-modified nucleic acid strand that was complementary to the single stranded patterned tethers (a), associated with the surface.

**Figure 31:**
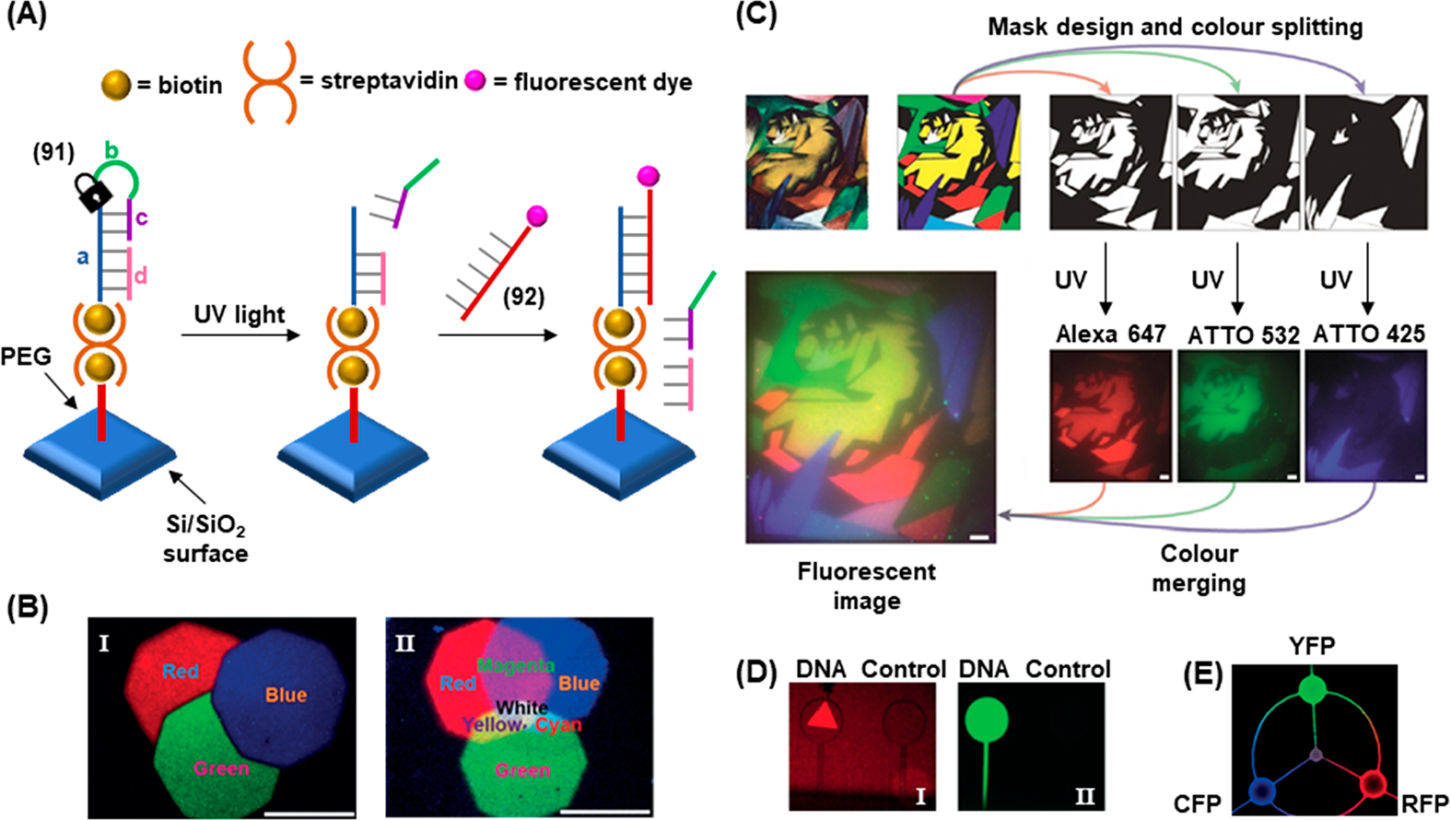
(A) Photolithographic patterning of an ONB-functionalized DNA and the subsequent association of fluorophore-modified strands to the deprotected monolayer domains. (B) Three-color fluorescence pattern generated upon stepwise full-dose light deprotection of the monolayer through a hexagonal mask, Panel I, and partial dose light-induced deprotection of the monolayer, leading to mixed fluorescent colors in the photopatterned domains, Panel II. (C) Construction of a multicolor image by the sequential patterning of extracted red/green/blue colors leading to the reproduced multicolor image upon merging. (D) Photolithographic gene patterning on a target surface for guided gene expression. The yellow fluorescent protein (YFP) gene is photopatterned and imaged by a fluorescent label, Panel I. Subsequently the patterned gene is used to express YFP, Panel II. (E) Patterning of three different genes corresponding to yellow (YFP), red (RFP), and cyan (CFP) fluorescent proteins in different surface containments followed by the guided expression of the respective proteins in the patterned domains. Panel (B)–(D) adapted with permission from ref. (331). Copyright 2018, John Wiley and Sons.

A further example applied photocleavable DNA hairpins for the precise photolithographic patterning of genes on a surface and the creation of complex multicolored images (Figure 31).^331^ In this system, a silicon/silicon oxide chip surface was passivated with biotinylated polyethylene glycol, which allowed the subsequent attachment of a biotinylated photocleavable DNA hairpin (**91**) to the surface using a bridging streptavidin linker, Figure 31A. The photocleavable DNA strand (**91**) consisted of a 40 nt stem region (a), a 5 nt single-stranded loop (b) that included the photocleavable ONB moiety, and a short 8 nt sequence complementary to the stem (c). An additional 30 nt blocking strand (d) was further hybridized with the stem domain. Without photoirradiation, the system was inert to the invading fluorophore-labeled strand (**92**) owing to the hybridization of the stem of (a) with the blocking domains (c) and (d). However, upon photoirradiation, cleavage of the hairpin loop caused dissociation of the (a)/(c) duplex, exposing an 8 nt toehold and allowing displacement of the remaining blocking strand (d) through toehold-mediated strand displacement by strand (**92**), leading to deposition of the fluorescent dye on the surface. The use of a photomask allowed spatial resolution of the surface patterning to be achieved. Fluorophores of different colors were deposited using consecutive photocleavage/hybridization cycles. Figure 31B depicts the result of the sequential immobilization of red (AlexaFluor 647), green (ATTO 532), and blue (ATTO 425) fluorophores at specific regions of the surface through an octagonal mask. During each step, the mask was moved to illuminate a region of the surface that partially overlapped with the region illuminated in the previous step, allowing the effect of the irradiation dose to be investigated. Long exposure times led to almost complete photocleavage of exposed regions during each step, resulting in single-color patterning of each region (Panel I). However, at shorter irradiation times, only partial photocleavage was achieved in each step and thus overlapping regions contained both fluorophores, allowing the generation of mixed colors (Panel II). The power of the technique for highly controlled patterning was demonstrated by the reconstruction of a complex multicolor structural image on the surface, e.g., a tiger pattern, Figure 31C. From a simplified version of the original artwork, three masks corresponding to red, green and blue coloration were derived and the three colors successively pattered on the surface using the multistep photopatterning procedure. By merging the fluorescence images generated by the individual colors, the complex image was reconstituted with high fidelity to the original. Finally, the lithographic technique was used for the precise positioning of gene expression machinery on a chip Figure 31D. A gene coding for a fluorescent protein was conjugated to invader strand (**92**) and deposited at a spatially precise region of the chip surface at a density of 100 units μm^–2^. The region containing the gene was then enclosed in a compartment which contained a narrow channel to a reservoir supplying gene expression machinery. A fluorescence reporter was also included on the invader strand to verify the successful spatial localization of the gene on the surface. Figure 31D, Panel I shows the successful deposition of the gene in a triangular shape, while an analogous control compartment masked from photoirradiation remained dark, confirming the absence of the gene in this region. After exposure of the system to gene expression machinery, the formation of the fluorescent protein product was clearly observed in the well containing the gene, while the control well, containing no photopatterned gene, remained dark (Panel II). Finally, three genes coding for different colors (yellow YFP, cyan CFP and red RFP) of fluorescent protein were simultaneously immobilized in different regions of a single chip. Exposure to the gene expression machinery led to the localized expression of the corresponding proteins, Figure 31E.

**Figure 32:**
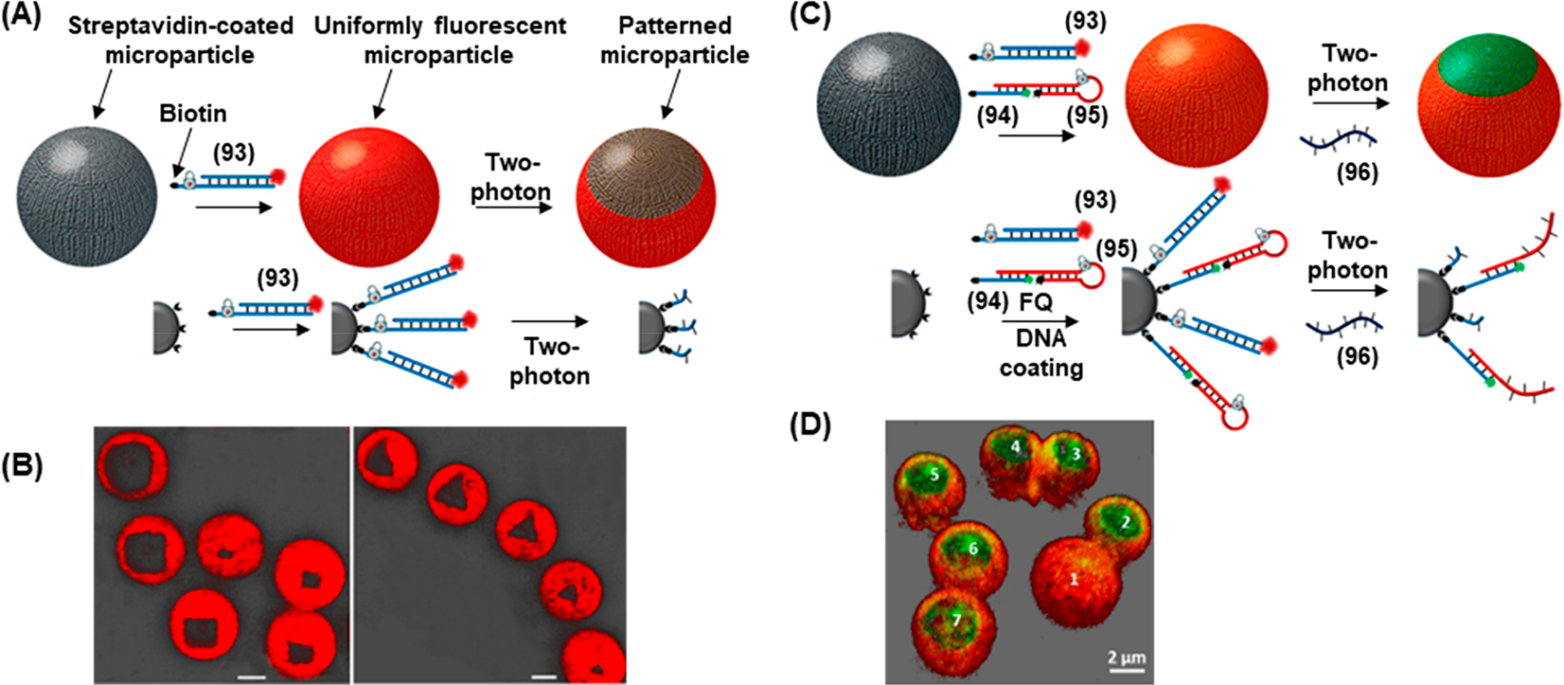
(A) Photolithographic patterning of ONB-functionalized red-fluorophore-modified duplex monolayer coated microparticles using a scanning confocal microscope equipped with a laser source. The two-photon laser stimulated deprotection of the ONB groups leads to patterned nonfluorescent domains on the particles. (B) Square or triangle shaped patterns generated on the microparticles. (C) Schematic two-color patterning of microparticles; microparticles are coated with a monolayer mixture of ONB-functionalized red fluorophore-modified duplex and a ONB-functionalized hairpin-modified quencher hybridized with a green fluorescence strand, yielding a caged structure exhibiting quenched green fluorescence. The composite monolayer-modified microparticles yield continuous red fluorescence. Scanning confocal microscopy aided two-photon laser photopatterning of the microparticles leads to photodeprotection of the ONB units associated with the two fluorescent constituents associated with the patterned domain. This leads to the release of the red fluorescenct constituent from the coating and to the ungaging of the green fluorescent constituent yielding a green pattern on the red fluorescent background coating. (D) Circular green fluorescent doains patterned on the red fluorescent coated microparticles. Figure adapted with permission from ref. (334). Copyright 2019, American Chemical Society.

Beyond the patterning of flat surfaces, photocleavable DNA also allowed the resolved lithographic patterning of spherical microparticle surfaces (Figure 32).^334^ In one system, Figure 32A, streptavidin-coated microparticles were coated with a biotinylated photocleavable DNA strand (**93**) hybridized with a complementary sequence tagged with the TAMRA fluorophore to generate uniformly fluorescent microparticles. Irradiation of specific regions of the microparticle surface resulted in spatially defined cleavage of the TAMRA-labeled duplex from the microparticle, generating dark regions of the surface. The photocleavage reaction could be achieved with one-photon (λ = 365 nm) or two-photon (λ = 740 nm) activation. Spatial resolution in the *x*, *y* and *z* planes was achieved with confocal microscopy and a femtosecond laser beam focused by a 63× oil objective to achieve a spot diameter of 70 nm. Thus, a variety of well-resolved shapes in different sizes could be created on the surface of the microparticles without the requirement for a photomask, Figure 32B. In a more complex patterning system, Figure 32C, the microparticles were functionalized with two photocleavable DNA strands to allow dual-patterning in two colors. The first strand (**93**) was functionalized with the TAMRA fluorophore. The second strand (**94**) functionalized with a different fluorophore (AlexaFluor488) was attached directly to the microparticle, and the fluorescence of this fluorophore was inactivated by hybridization with a hairpin (**95**) containing a fluorescence quencher (BHQ-1) and a photocleavable loop region. Photocleavage of the loop in this case leads to the exposure of a toehold on the quencher-bearing strand, allowing displacement of the quencher from the microparticle surface by invader strand (**96**) through toehold-mediated strand displacement, activating the Alexa Fluor 488 fluorescence. Figure 32D shows the three-dimensional reconstruction of the fluorescence image of seven microparticles either nonirradiated (Particle 1) or irradiated in different regions (Particles 2–7). In nonirradiated regions, the TAMRA fluorophore remained attached and the AlexaFluor488 fluorophore remained quenched, causing these regions to appear red. Irradiation of specific surface regions caused detachment of the TAMRA-labeled strands and simultaneous activation of AlexaFluor488 fluorescence (following the addition of the invader strand to displace the photocleavage product strand) resulted in green fluorescence in these areas.

### 4.9 Photodeprotection of ONB-Nucleic Acid Functionalized Hydrogel Materials

Hydrogels represent a diverse class of soft materials that have attracted broad scientific interest.^335−337^ Various applications of hydrogels were suggested including their use for separation,^338^ sensing,^339^ controlled release,^340−343^ and tissue engineering.^344,345^ Particularly, stimuli-responsive hydrogel matrices revealing controlled stiffness properties were demonstrated.^346^ Auxiliary signals such as pH,^347,348^ chemical agents,^349^ magnetic and electrical fields,^350−353^ light,^354−356^ and redox signals^357^ were applied to switch the stiffness of hydrogels and different applications of these matrices for controlled drug release,^358^ switchable catalytic materials,^359^ self-healing,^360,361^ and robotics^362,363^ were introduced. Within the framework of stimuli-responsive hydrogels, DNA-based hydrogels represent an important subclass of materials.^364,365^ The triggered reconfiguration of DNA structures provides versatile means to control the cross-linking and stiffness of DNA hydrogels. Indeed, different switchable triggers such as G-quadruplex/crown ether^359^ or the pH-induced formation and separation of i-motif or triplex structures^366^ were used to control the stiffness of bulk DNA-based hydrogels or of DNA-based hydrogel microcapsules loaded with drugs. Different applications of the DNA hydrogels for controlled drug release,^366^ self-healing/shape memory^367^ and fabrication of actuators^368^ were demonstrated. Not surprisingly, light was applied to trigger stimuli-responsive DNA-based bulk hydrogels and hydrogel microcapsules. These included the reversible application of photoisomerizable intercalators, e.g. *trans–cis* azobenzene,^367^ the use of thermoplasmonic gold nanoparticles or gold nanorods,^126^ and the application of photoprotecting o-nitrobenzyl phosphate functionalities linked to DNA.^369,370^

**Figure 33:**
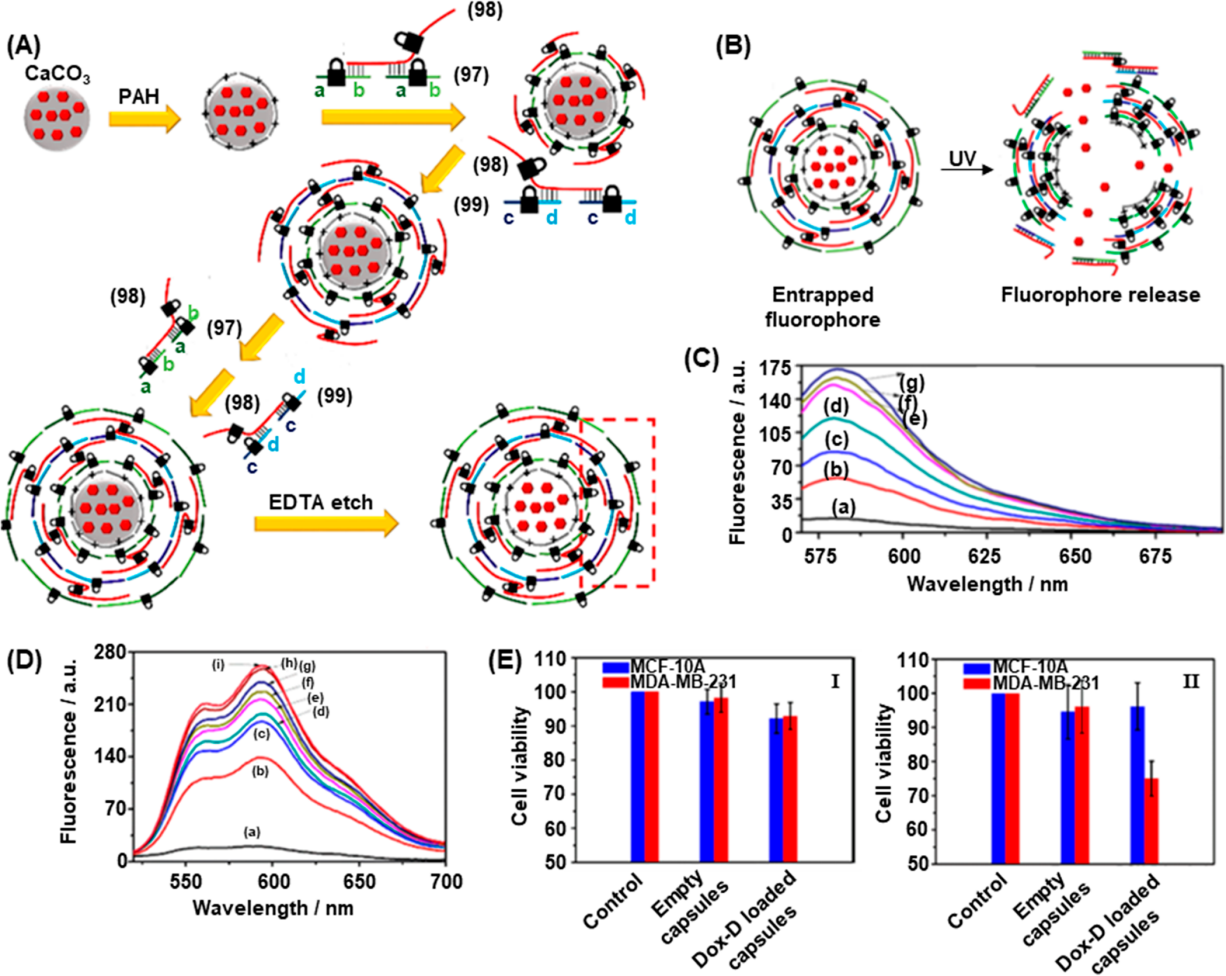
(A) Stepwise synthesis of ONB-modified nucleic acid-based tetramethyl rhodamine dextran (TMR-D) loaded microcapsules. (B) Light-induced degradation of the ODN-modified microcapsules resulting in the release of the load. (C) Fluorescence spectra of TMR-D released from the ONB-modified microcapsules irradiated for different time intervals: (a) 0 s to (g) 5 min. (D) Fluorescence spectra of doxorubicin-dextran (Dox-D) released from the ONB-modified microcapsules upon irradiation for different time intervals: (a) 0 s to (i) 120 s. (E) Cytotoxicity of the Dox-D-loaded microcapsules toward MDA-MB-231 breast cancer cells (red) and epithelial MCF-10A cells (blue) and measuring cell viability after 24 h: Panel I – without irradiation and deprotection of the microcapsules. Panel II – after irradiation for 5 min. Figure adapted with permission from ref. (370). Copyright 2016, American Chemical Society.

Photocleavable DNA strands were successfully applied to engineer light-responsive microcapsules engineered to release a therapeutic cargo in response to photoirradiation, thus allowing spatiotemporal control of drug release (Figure 33).^370^ The microcapsules were engineered by sequential deposition of photocleavable DNA layers onto a prefabricated calcium carbonate microparticle containing the desired load (encapsulated by coprecipitation) and coated with a layer of PAH. The positively charged nature of the PAH enabled it to act as a primer layer for the subsequent coating with DNA duplex (**97**)/(**98**) by electrostatic interactions, while subsequent DNA layers of strands (**97**)/(**98**) and (**98**)/(**99**) duplexes were deposited through strand hybridization. Following the assembly of the multilayered capsule, the calcium carbonate template was etched away with ETDA, leading to hollow capsules containing the payload, Figure 33A. The DNA strands (**97**), (**98**), and (**99**) were engineered to contain the photoresponsive ONB linker such that the capsule structure was broken upon photoirradiation with UV light, Figure 33B. The controlled release of a fluorophore (tetramethyl rhodamine–dextran, TMR-D) was demonstrated. Figure 33C depicts the fluorescence traces of the released fluorophore following exposure of the capsules to UV light for different irradiation times from 0 s (curve a) to 120 s (curve g), after a 30 min release period following irradiation. For nonirradiated capsules, negligible fluorophore was released as the capsules remained intact. Increasing the irradiation dose led to a greater proportion of cleaved microcapsules, increased release of TMR-D, and greater fluorescence intensity.

The photocleavable microcapsules were then deployed to enable the light-triggered release of dextran-conjugated doxorubicin (Dox-D), a potent anticancer agent. Conjugation to dextran was necessary as free doxorubicin was sufficiently small to leak out of the capsules even before photocleavage. Figure 33D depicts the fluorescence spectrum of released Dox-D following different irradiation times of the capsules, after a 1 h release period following the cessation of irradiation. In the absence of photoirradiation, negligible release was observed (curve a) while irradiation doses of 30 s–5 min led to increasing quantities of released proportional to the irradiation time. Finally, the cellular uptake and cytotoxicity of the Dox-D-loaded capsules was investigated. The microcapsules were internalized well by malignant MDA-MB-231 breast cancer cells while poor uptake was observed in normal MCF-10A cells. Intact capsules loaded with Dox-D demonstrated no toxicity to either cell line in the absence of photoirradiation (Figure 33E, Panel I) as the drug was inactive when trapped within the capsule. However, after photoirradiation (Panel II) of cells containing the loaded microcapsule, significant toxicity to the MDA-MB-231 cells was observed, consistent with the temporally controlled release of the drug in these cells. Photoirradiation of the MCF-10A cells exposed to capsules did not result in appreciable toxicity, since the capsules were not efficiently internalized by these cells and Dox-D was only released in the extracellular solution. Control experiments demonstrated that cells loaded with empty capsules remained fully viable, even after photoirradiation, demonstrating the observed toxicity was a direct result of the stimuli-triggered release of the drug payload. These results demonstrate the potential use of doxorubicin-loaded photocleavable capsules as photoresponsive therapeutic drug carriers.

**Figure 34:**
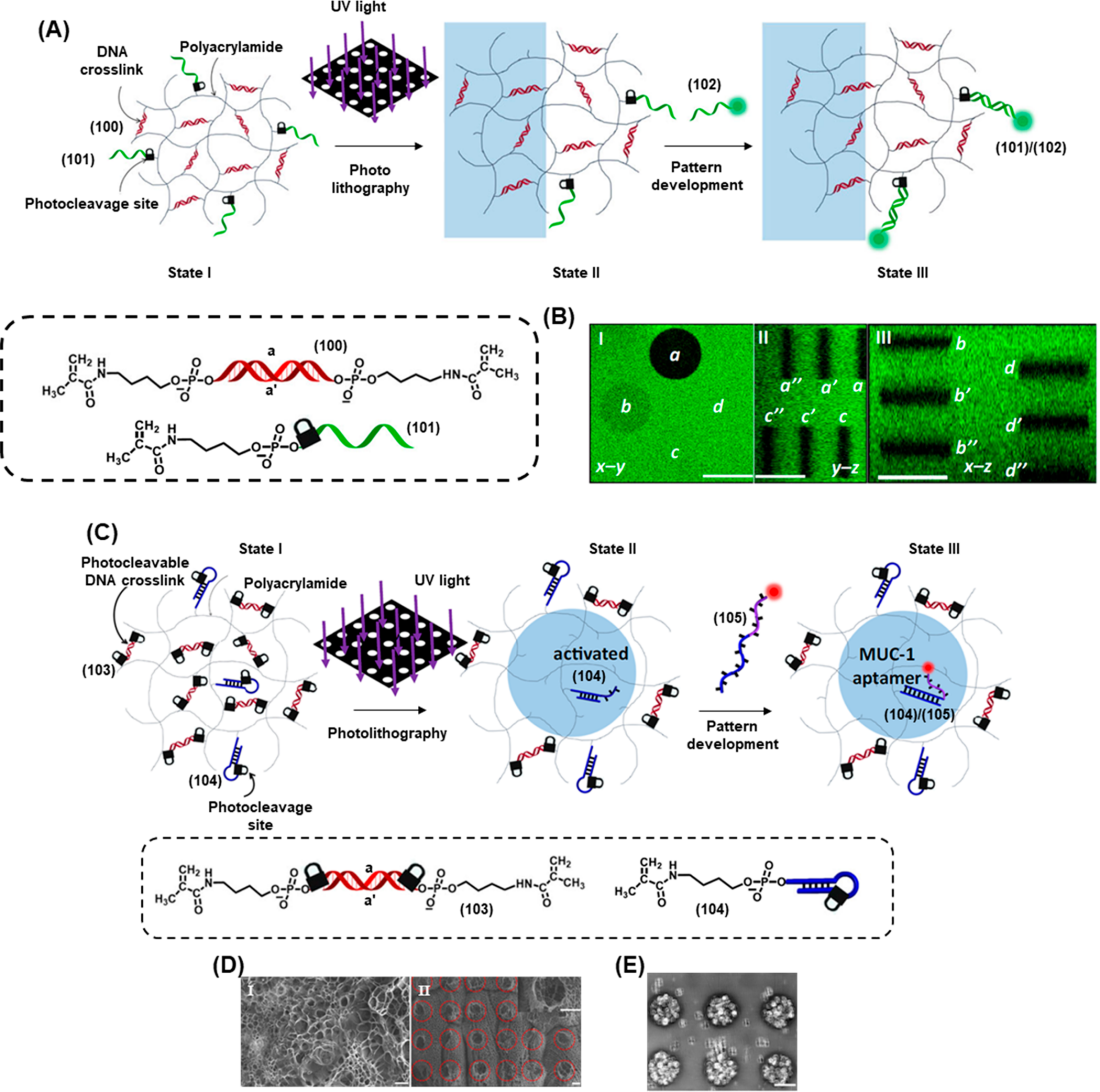
(A) Schematic photolithographic patterning of an ONB nucleic acid functionalized polyacrylamide hydrogel. (B) Fluorescent image of the photodeprotected patterned hydrogel. (C) Schematic photopatterning of an ONB nucleic acid functionalized cross-linked hydrogel matrix leading to photopatterned circular low stiffness quasi-liquid hydrogel domains with toehold functionalized duplex constituents. The subsequent association of the MUC-1 aptamer to the soft patterned domains guides the binding of HeLa cells to the patterned regions. (D) SEM image of the ONB-functionalized hydrogel before deprotection (Panel I) and after light-induced deprotection of the ONB units confirmed by the circular domains of the patterned hydrogel (Panel II). (E) HeLa cells caught in the patterned domains modified with the MUC-1 aptamer. Figure adapted with permission from ref. (369). Copyright 2021, the Authors.

Photocleavable DNA-based hydrogel structures have also been realized (Figure 34).^369^ The initial system was constructed by polymerization of acrydite units consisting of the a/a′ duplex strand (**100**) and the single strand (**101**) to generate the hydrogel matrix consisting of a polyacrylamide scaffold permanently cross-linked by the a/a′ domain of duplex (**100**) and functionalized with photoresponsive strand (**101**) (Figure 34A, State I). The resulting material was photopatterned through a mask containing a pattern of circular holes. Irradiation through the mask cleaved the photoresponsive strand (**101**) from the hydrogel in the irradiated region (blue) while nonirradiated regions remained functionalized with strand (**101**), State II. Following the washing away of the cleaved strands from the hydrogel, the addition of the complementary strand (**102**), modified with a fluorophore, led to labeling of the nonirradiated regions by formation of the (**101**)/(**102**) duplex, State III. The formation of the circular mask pattern on the surface of the hydrogels was observed. Moreover, mask-free lithography was also achieved by employing a two-photon laser scanning confocal microscopy to perform irradiation (λ = 740 nm), allowing the precise three-dimensional patterning throughout the depth of the hydrogel. Figure 34B depicts the three-dimensional patterning of a hydrogel film in which the circular pattern in the *x*–*y* plane (Panel I) is repeated at three focal depths along the *y*–*z* plane (Panel II) and *x*–*z* plane (Panel III) of the hydrogel.

By engineering photocleavable moieties into the cross-linking strands, it was possible to pattern spatially separated non-cross-linked circular domains in the hydrogel matrix (Figure 34C). Accordingly, the hydrogel was engineered to include the photocleavable duplex a/a′ (**103**). Irradiation of regions of the hydrogel through the hole-patterned mask therefore generated separated “hole” domains of non-cross-linked polyacrylamide within a surrounding rigid, cross-linked region composed of the nonirradiated bulk of the hydrogel matrix. Figure 34D shows the SEM images of the hydrogels before (Panel I) and after (Panel II) photoirradiation through the mask. After photoirradiation, the non-cross-linked domains are visible as “holes” in the hydrogel structure. Furthermore, the photoresponsive DNA hairpin (**104**) was also incorporated into the hydrogel as a functional handle. In the rest state, the hairpin contains a photocleavable linker in its loop region and is inert to hybridization to complementary strand (**105**). Upon photocleavage of the circular hydrogel domains, in addition to the cleavage of the duplex units (**103**), a photocleaved toehold-modified duplex generated by photocleavage of the hairpin (**104**) was formed. Hybridization of the fluorophore-labeled strand (**105**) by displacement of the toehold duplex allowed the fluorescent readout of the patterning process. Thus, irradiation of the hydrogel through the circular mask both formed the non-cross-linked domains and activated the resulting “wells” toward functionalization with strand (**105**) or any other functional strand. This approach was used to activate the hydrogel matrix toward the selective binding and proliferation of cells. By engineering the strand (**105**) to include the mucin 1 protein (MUC-1) aptamer sequence, the photopatterning strategy leads to the deposition of this aptamer in the soft wells of the hydrogel after photopatterning. As HeLa cells express MUC-1 on the cell surface, they are selectively absorbed onto the hydrogel in these regions through the MUC-1/aptamer interaction. Figure 34E shows the bright-field image of a hydrogel section that was photopatterned to contain the circular, MUC-1 aptamer functionalized wells after incubation with HeLa cells for 2 h. Selective deposition of the HeLa cells within the circular wells was observed, while cells were almost completely absent in areas of the hydrogel that where shielded from photoirradiation, since these remain both fully cross-linked (intact strand **103**) and inert to hybridization with the MUC-1 aptamer target (intact strand **104**). The photopatterning strategy was also employed to confine HCR to specific regions of the hydrogel surfaces, thus demonstrating the versatile approaches to hydrogel patterning and functionalization afforded by the photocleavable DNA strategy.

### 4.10 ONB-Functionalized DNA Origami Nanostructures

DNA origami structures attract growing interest as two-dimensional scaffolds for the precise positioning of nano-objects such as nanoparticles or macromolecules.^296,371−376^ The two-dimensional origami rafts are formed in diverse shapes by applying computer programmed stapler units that tie together a long viral DNA into the desired structure.^377^ By applying appropriately tethered “stapler” units, origami rafts containing protruding nucleic acid tethers in preprogrammed positions on the upper or lower faces of the tiles or at the edges of the tiles may be engineered. While the protruding tethers at the upper and lower faces may act as anchoring positions for nucleic acid-modified proteins, nanoparticles, or supramolecular DNA constructs through hybridization, the edge functionalization of origami tiles with origami tethers, and particularly, stimuli responsive tethers, the guided and triggered reversible dimerization or oligomerization of origami tiles were demonstrated.^102,103,280,378^ Indeed, the programmed engineering of functional domains on the origami tiles enabled the patterning of the scaffolds and the sequestered patterned positioning of proteins^296,371,372,374,375^ or nanoparticles,^373,376,379^ the triggered programmed motility of nanoobjects along predefined pathways, e.g. walkers,^373,380−382^ the spatial proximate positioning of enzymes and the activation of enzyme cascades,^371,372^ and to the design of gated drug carriers.^296^ Not surprisingly, photochemical control over the functions and properties of the origami rafts by means of photoisomerizable reversible units, e.g. *trans*–*cis* azobenzene units^153,383−386^ or single-cycle photoresponsive ONB-protected DNA functionalities gained scientific interest.

**Figure 35:**
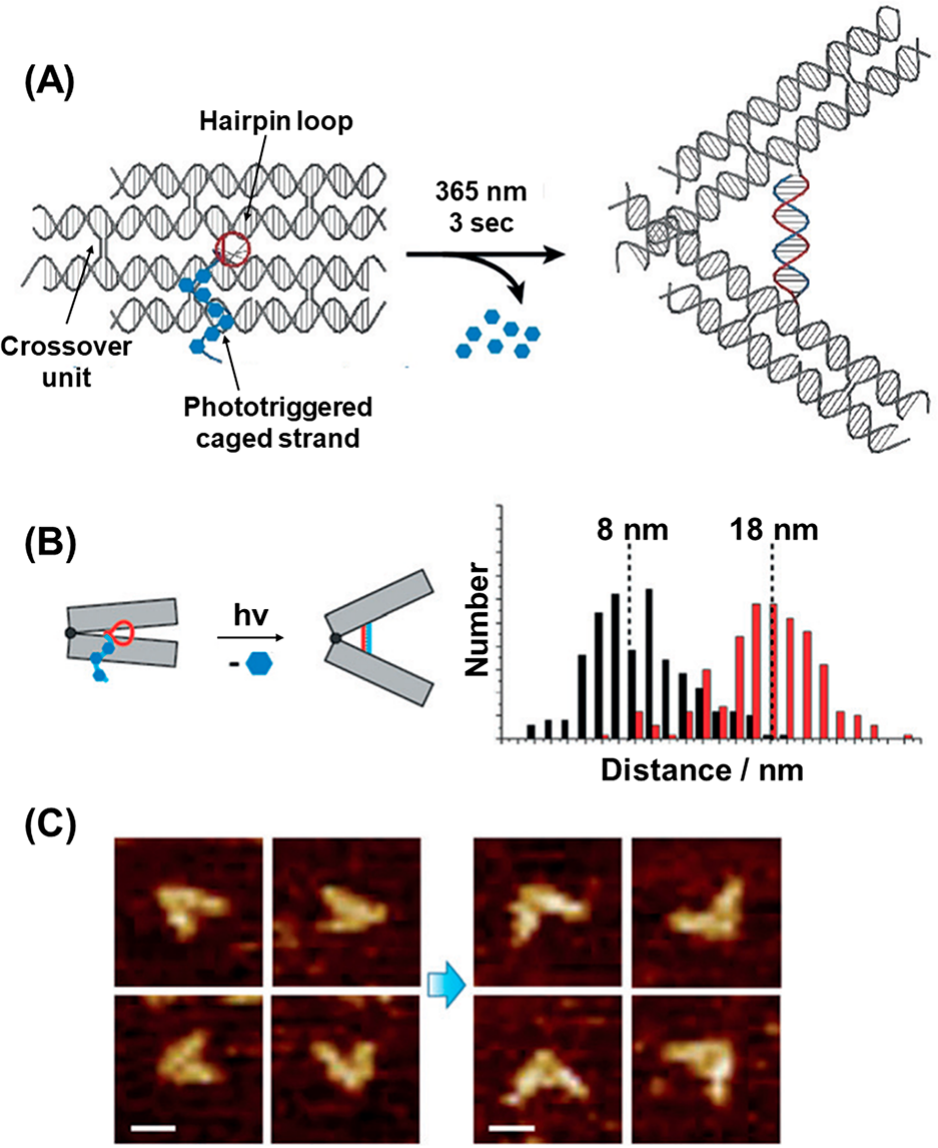
(A) Schematic light-induced “mechanical” unlocking of a closed ONB-locked origami bundle tweezers-type structure leading upon deprotection to an open tweezers structure. (B) Schematic light-induced unlocking and opening of the tweezers structure, accompanied by a histogram corresponding to the populations of closed tweezer structures before illumination and the open tweezers after illumination. (C) Examples of the closed origami bundle tweezer before light-induced separation (left) and after light-induced deprotection of the ONB units and opening of the tweezers (right). Figure adapted with permission from ref. (387). Copyright 2018, John Wiley and Sons.

Photocaged DNA strands were directed toward the realization of a light-responsive DNA nanotweezer (Figure 35).^387^ The tweezer consisted of two arms linked by a crossover unit, Figure 35A. In the initial state, the tweezer was locked closed by the presence of an A-rich hairpin loop between the two arms. The structure was also engineered, however, to contain a caged poly-T strand in proximity to this loop, where hybridization to the hairpin region was prevented by functionalization of seven of the thymidine bases with NPOM photoresponsive caging groups. Upon irradiating the device with a brief pulse (3 s) of 365 nm UV light, the NPOM groups were cleaved which unmasks the hydrogen bonding sites associated with the thymidine residues. Thus, hybridization of the uncaged strand with the hairpin loop proceeded to form an elongated duplex bridging unit which generated the open state of the tweezer. The open/closed states of the tweezers were probed by AFM analysis, Figure 35B and Figure 35C, which revealed a distance distribution of 8 nm between the ends of the tweezer arms when closed, while following photoirradiation to generate the open state of the tweezer, a greater distance of 18 nm was observed. The open and closed states of the tweezer were distinguishable in the AFM images, including the duplex region that holds the tweezers in the open state following photoirradiation. The kinetics of the tweezer opening were also studied, demonstrating that 90% of tweezer opening occurred after only 15 s following the pulse of photoirradiation. The fast kinetics were attributed to the high local concentration of trigger strand because it was incorporated directly into the tweezer structure.

**Figure 36:**
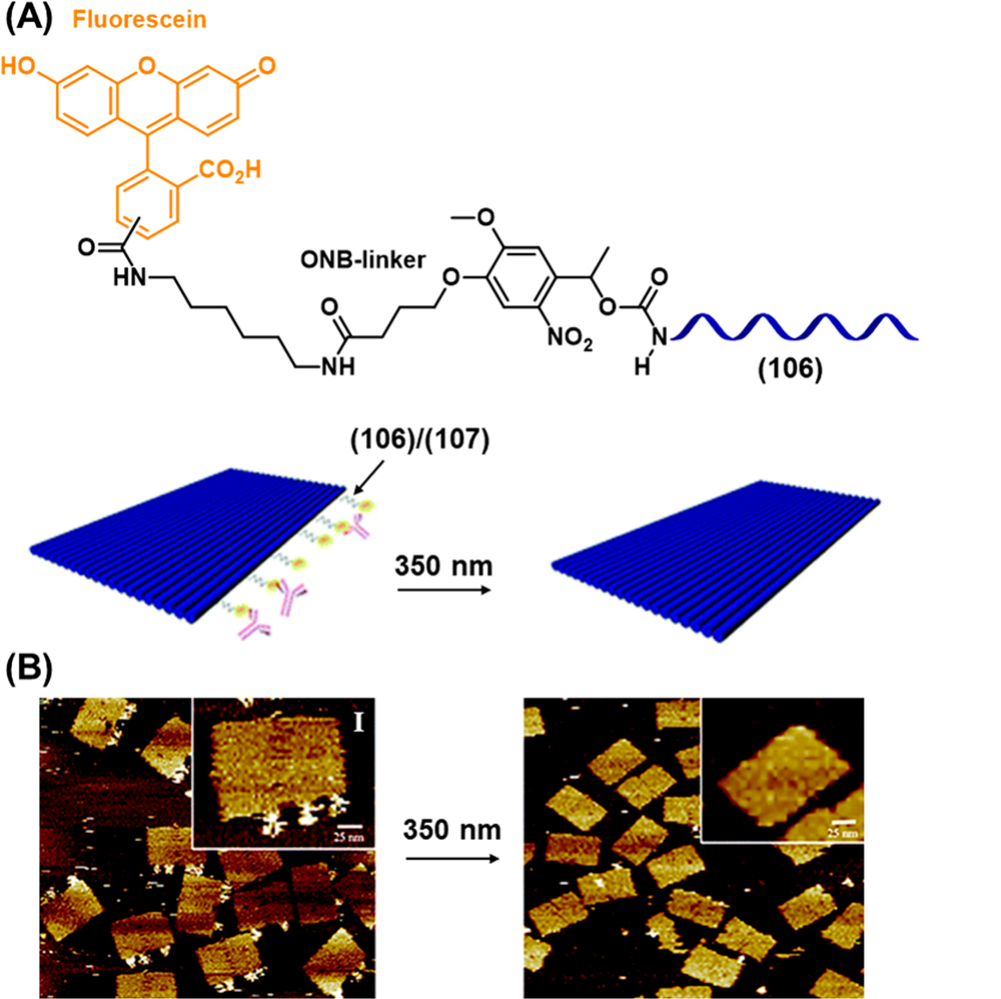
(A) Assembly of a photoresponsive ONB-protected origami tile/antifluorescein antibody hybrid system through the binding of the fluorescein (antigen)-ONB-protected nucleic acid (**111**) to the complementary origami tile edge-modified tether (**112**), and the light-induced separation of the hybrid by deprotection of the ONB linkage. (B) AFM images of the hybrid origami tile fluorescein conjugate before deprotection, Panel I, and after light-stimulated deprotection, Panel II. Figure adapted with permission from ref. (388). Copyright 2015, Royal Society of Chemistry.

The phototriggered dissociation of constituents attached to the edges of origami tiles was demonstrated, Figure 36.^388^ A photoresponsive ONB-functionalized nucleic acid tether (**106**) consisting of the fluorescein antigen linked to the nucleic acid tether through the ONB moiety was synthesized, Figure 36A. The origami raft was engineered to include at its edges complementary tether strands (**107**) to the fluorescein/ONB nucleic acid strand (**106**). The functionalization of the raft with the antigen-modified strand by hybridization of (**106**)/(**107**), followed by the binding of antifluorescein antibody to the antigen anchoring site, generated the origami raft-antibody conjugate. Irradiation of the origami conjugates, λ = 350 nm, resulted in the cleavage of the photoresponsive ONB linkers, leading to the dissociation of the origami raft antibody conjugate. The process was imaged by AFM, Figure 36B.

**Figure 37:**
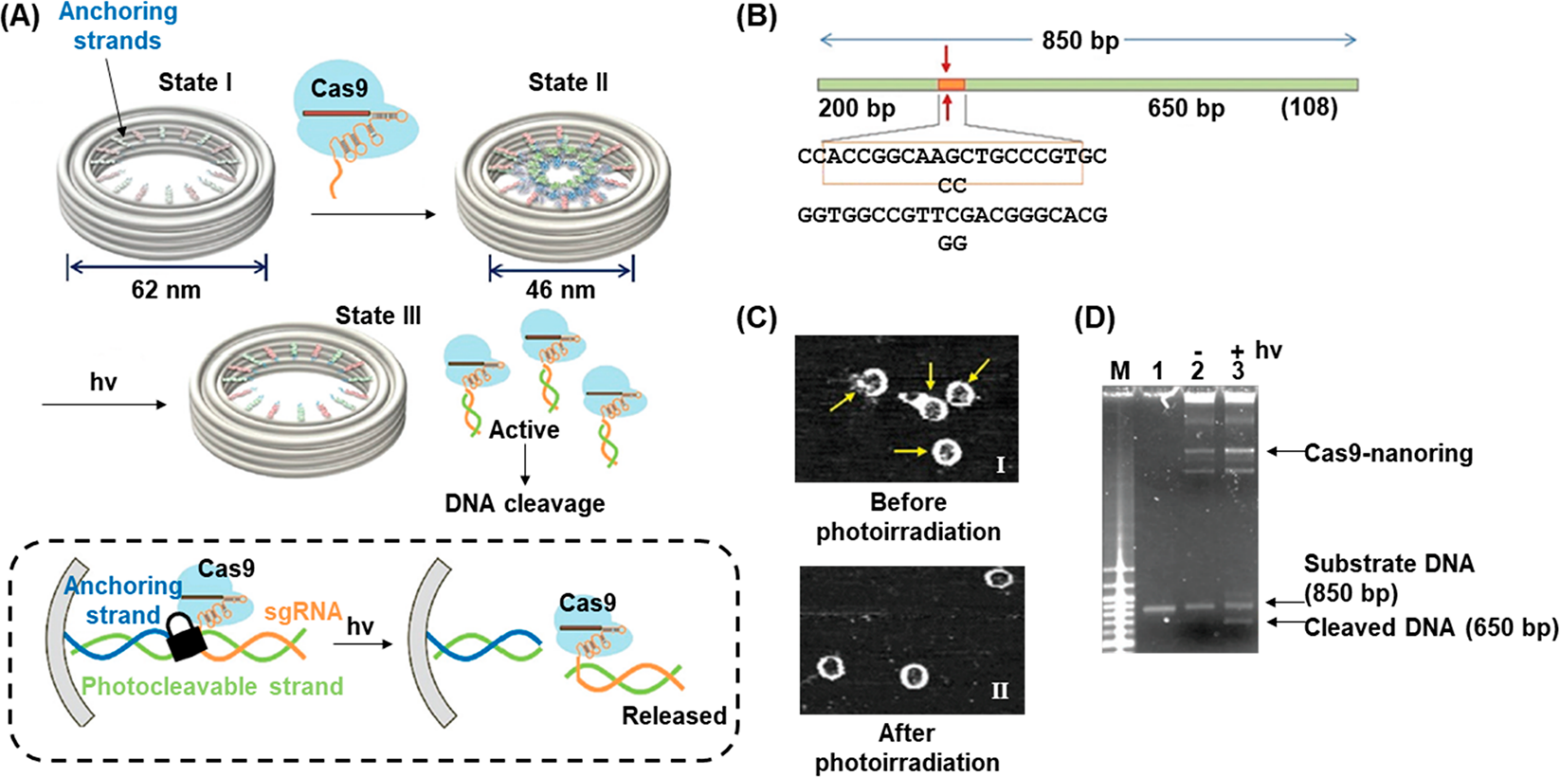
(A) Schematic photochemical activation of the CRISPR-Cas9 machinery caged within an origami DNA ring toward the guided cleavage of a target site in a DNA duplex. The single guide RNA (sgRNA) Cas9 complex is inactivated by trapping in the ring via an ONB-protected strand linked to an anchoring strand protruding in the cavity of the origami ring. (B) The light induced deprotection of the Cas9-sgRNA machinery leads to the site-specific sgRNA-guided cleavage of the target duplex substrate. (C) AFM images corresponding to Panel I – the Cas9-sgRNA caged machinery in the DNA origami ring. Panel II – the vacant origami ring after deprotection of the ONB caging unit. (D) Electrophoretic analysis of the Cas9-sgRNA-guided site-specific cleavage of the duplex shown in (B). The cleavage band is only observed after light-induced release of the Cas9-sgRNA machinery from the cavity of the nanoring. Figure adapted with permission from ref. (389). Copyright 2021, Royal Society of Chemistry.

Optical control of a CRISPR-Cas9 system based on a photocleavable DNA origami strategy was reported (Figure 37).^389^ An origami nanoring of 62 nm in diameter was engineered to include 24 anchoring strands projected into the central cavity Figure 37A, State I. A photocleavable strand (inset) that bridges between the anchoring strands and the single-guide RNA (sgRNA) on the Cas9 protein was employed to entrap the protein inside the nanocavity (State II). The sgRNA was designed to target an 850 bp DNA duplex (**108**) to facilitate the Cas9-mediated cleavage of the strand into two shorter fragments of 200bp and 650bp, Figure 37B. Irradiating the nanoring/Cas9 assembly at 350 nm for 5 min cleaved the photoresponsive bridging strand, leading to release of the Cas9 from the cavity (State III). AFM imaging, Figure 37C demonstrated the encapsulation of the Cas9 (Panel I) and subsequent release following photocleavage (Panel II). Having achieved the controlled and reversible localization of Cas9 inside the nanoring, the effect of entrapment and subsequent release on the activity of the Cas9 was investigated by subjecting the system to strand (**108**). Gel electrophoretic analysis of the system, Figure 37D, demonstrated that before photoirradiation, with the Cas9 incorporated inside the nanoring, no cleavage of the 850 bp DNA was observed (Lane 2), indicating the activity of the enzyme was deactivated through encapsulation, likely because the diameter of (**108**) was too large to enter the ring, preventing the access to the catalytic site of Cas9 in the encapsulated state. After photoirradiation to trigger the release of the Cas9, cleavage of the target DNA was clearly observed by the presence of a faster-running band in electrophoresis experiments (650 bp) corresponding to a cleavage yield of 46% (Lane 3). These results suggest potential promising applications toward the photoregulation of gene editing systems.

**Figure 38:**
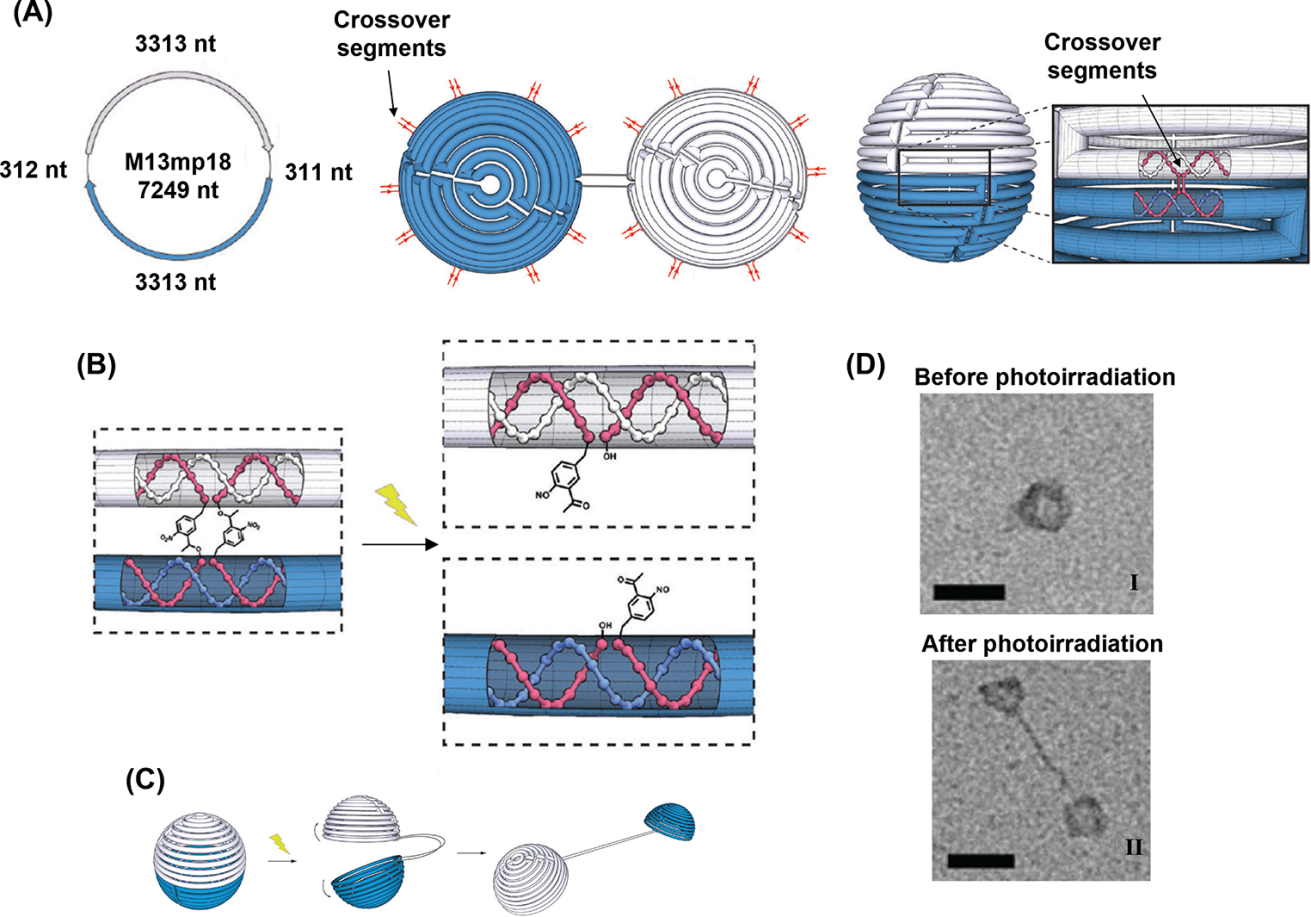
(A) Schematic assembly of a photoresponsive ONB-protected origami sphere consisting of two interlinked origami hemispheres bridged into the sphere structure by complementary ONB-functionalized tethers protruding from the hemisphere edges. (B) Photodeprotection of the hemisphere bridging duplex leads to the separation of the sphere into two tethered hemispheres. (C) AFM images corresponding to Panel I – the intact sphere structure composed of the ONB duplex bridged edges of the hemispheres. Panel II – the separation of the sphere into two tethered hemispheres unpon the light-induced deprotection of the crossover ONB-functionalized bridging units. Figure adapted with permission from ref. (390). Copyright 2015, Royal Society of Chemistry.

A different approach to incorporating photocleavable moieties into DNA origami structures used the photocleavage process to control the global structure of the origami itself. Figure 38 depicts a DNA origami sphere that was cleaved into two tethered hemispheres by photoirradiation.^390^ The sphere was constructed from a single m13mp18 viral DNA sequence, Figure 38A, using staple strands designed to assemble two 3313 nt hemispheres connected by two single-stranded tethers (312 nt and 311 nt). The two hemispheres were joined together via the judicious placement of nine crossover segments along the sphere equator. These segments were engineered to contain the photoresponsive ONB moiety at the joining point of the two spheres, Figure 38B, such that upon cleavage of this moiety by photoirradiation (302 nm, 10 min), the hemispheres were separated, yet remained bridged through the remaining single stranded tethers, Figure 38C. The successful folding of the origami into the intact spherical structure was observed by AFM, Figure 38D, Panel I, and the formation of the separated hemispheres following photoirradiation could be imaged, Panel II. Potential applications of the photocleavable spheres, for example the phototriggered release of drug loads, can be envisaged.

**Figure 39:**
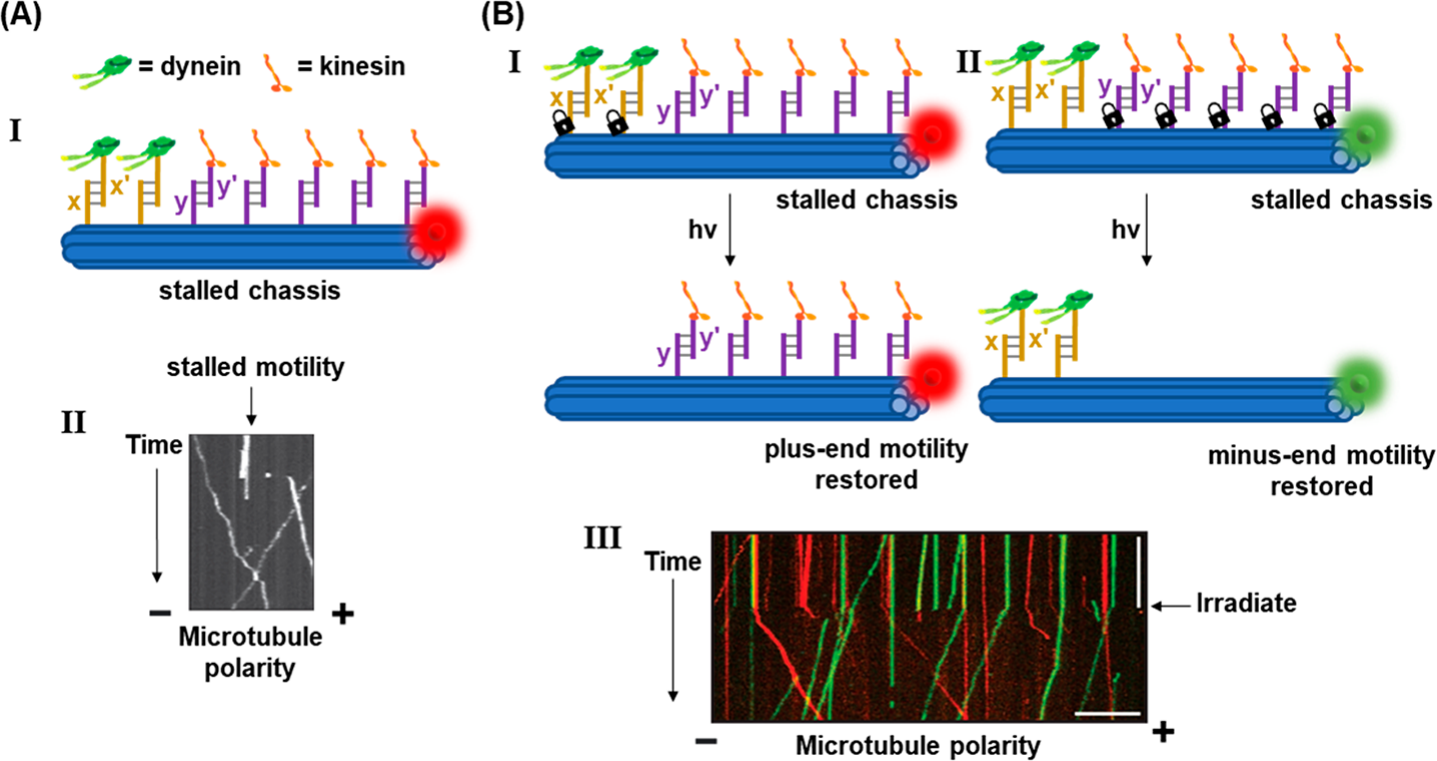
(A) Panel I – Assembly of two motor proteins, dynein (D) and kinesin (K), on a fluorescent DNA origami bundle acting as a chassis for motility along microtubules. The functionalization of D with nucleic acid x′ and modification of K with y′ allowed their linkage, through hybridization, to protruding nucleic acid anchors linked to the origami chassis. At a D:K ratio corresponding to 2:5, stalled motility of the chassis is observed, Panel II. (B) Functionalization of the DNA origami chassis with photocleavable x (Panel I) or photocleavable y (Panel II) yielded photoresponsive bundles modified by the motor proteins. Upon the selective light-induced removal of D (Panel I), the resulting K-rich origami restores, in the presence of ATP, motility toward the plus-end of the microtubule. In turn, photorelease of the K motor protein yields the D-rich origami chassis that restores, in the presence of ATP, motility toward the minus-end of the microtubule (Panel II). By labeling the two chassis with red or green fluorophores, respectively, the resulting restoration of motility of the stalled chassis upon light-induced deprotection is observed (Panel III). Figure adapted with permission from ref. (391). Copyright 2012, The American Association for the Advancement of Science.

A significant application of photocleavable DNA origami was directed toward interrogating the functions of microtubule motor proteins (Figure 39).^391^ The system targeted the “tug of war” effect that exists between two microtubule motors, dynein (D) and kinesin (K), which direct microtubule transport in opposite directions. A fluorophore-labeled chassis consisting of DNA origami bundles was engineered to contain protruding single-stranded DNA tethers acting as anchor sites for the attachment of DNA-functionalized dynein or kinesin units in dictated ratios, Figure 39A, Panel I. By following the motion of the chassis along the microtubules at the single-molecule level using total-internal-reflection fluorescence microscopy, the effect of different ratios of the antagonistic motors was probed. The conjugation of dynein and kinesin in a 2:5 ratio led to the observation of stalled motion, indicating the “tug-of-war” effect between the two motors, Figure 39A, Panel II. To activate motion of the chassis along the microtubule, a means for the selective detachment of one class of motor from the chassis was required. The incorporation of a photocleavable linker into the DNA anchors for the appropriate protein allowed the selective detachment of the respective protein from the chassis, Figure 39B, Panels I and II. The respective photoresponsive chassis were differentiated by labeling with fluorophores of different colors. Upon cleaving the respective protein from the chassis using UV light, motion was restored in the direction of the remaining motor, Figure 39B, Panel III.

### 4.11 Photodeprotection of ONB-Nucleic Acid Probes for Coded Sensing

Single DNA strands act often as promoter strands to activate DNA machineries such as polymerization/nicking machineries,^392−394^ or RCA^246,247^ pathways in the presence of appropriately engineered promoter-recognition templates or circular templates. Also, single-stranded promoter-activated HCRs in the presence of appropriately tailored hairpin structures,^243,395,396^ were applied for the autonomous dynamic synthesis of supramolecular DNA structures. By appropriate design of the DNA machinery scaffolds or machinery constituents, the dynamic synthesis of catalytic reaction products, e.g. DNAzymes, were demonstrated,^397,398^ and amplified sensing platforms for diverse analytes, such as genes,^399,400^ miRNAs,^401−403^ or aptamer-ligand complexes,^404^ were accomplished. Not surprisingly, the conjugation of nucleic acid strands to recognition units, such as antibodies, through *ortho*-nitrobenzylphosphate photoresponsive bridges, provide versatile means for the sequestered photodetachment of the strands from the recognition complexes and their subsequent amplification by different DNA machineries, leading to amplified and selective sensing platforms.^405,406^ This will be exemplified in the following section by introducing photocaged single stranded nucleic acid conjugates for amplified sensing by coupled DNA machineries.

**Figure 40:**
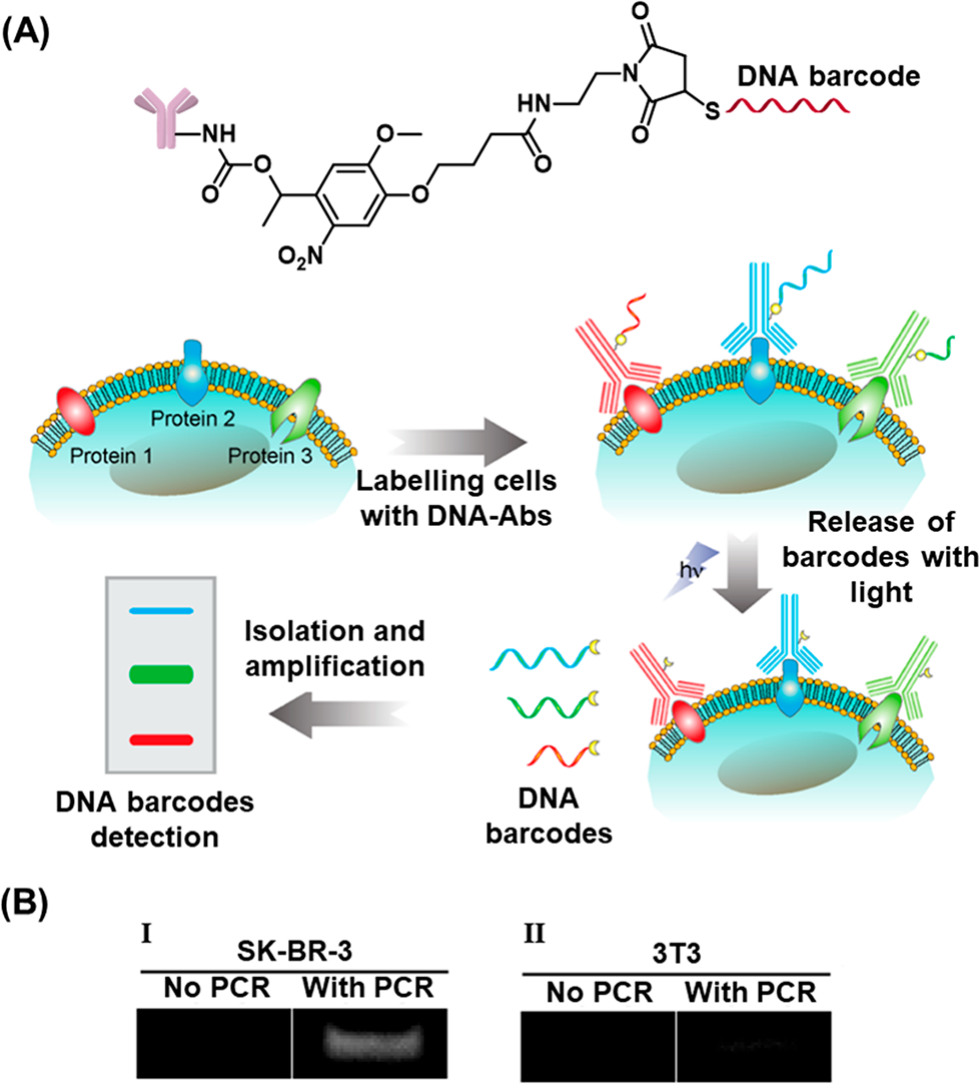
(A) Schematic probing of proteins associated with cell membranes using specific antibody-barcode nucleic acid conjugates bridged by photocleavable ONB bridges. The association of the antibody conjugate(s) to the specific protein followed by the UV-light deprotection of the cell-bound barcode(s) as labels and their PCR amplification provide the means to analyze the respective protein(s). The PCR-amplified barcode is analyzed by electrophoretic separation. (B) Electrophoretic analysis of: Panel I – SK-BR-3 cells, containing the HER2/*neu* receptor protein and Panel II – 3T3 cells lacking the HER2/*neu* receptor protein using the ONB-bridged barcode conjugated HER2/neu antibody using the light/PCR-amplified detection platform outlined in (A). Figure adapted with permission from ref. (405). Copyright 2012, American Chemical Society.

Photocleavable DNA-antibody conjugates to quantify protein expression on the surface of live cells were introduced, Figure 40.^405^ Antibodies for the protein or proteins of interest were conjugated to DNA strands (“barcodes”) of 55–80 bases in length via a bifunctional photocleavable linker, Figure 40A. Cells expressing complementary surface proteins to the antibody were labeled with the respective conjugate while those not expressing the protein remained unlabeled. After washing away the unbound antibody/barcode conjugates, the barcodes labeling the surface proteins were detached from the cells by light-induced cleavage of the ONB linker. Following PCR amplification (−25 cycles) and gel electrophoretic separation of the resulting barcode analytes, the intensity of the corresponding band allowed quantitative assessment of the target protein on the cell surface. Figure 40B, shows the gel electrophoretic images obtained following the exposure of two different cell lines with an antibody/barcode conjugate specific to the HER/*neu* surface protein. In the case of SK-BR-3 cells, Panel I, which express this protein on their surface, a clear band corresponding to the length of the DNA barcode were observed following PCR. Without photodetachment of the DNA barcode probe, no signal was observed. For 3T3 cells, which are HER/neu negative, no signal was detected after the same number of PCR cycles, as the cells were not labeled by the antibody/barcode conjugate, Panel II. The results also demonstrate the importance of combining the photocleavable labeling strategy with DNA technology. While the photoresponsive unit allows for the facile, noninvasive release of the DNA barcode from the cell surface following the washing away of unattached antibody/barcode conjugates, the amplification mechanism of the resulting analyte is made possible by the unique and specific self-replication (PCR) feature of the DNA label. As shown in Figure 40B, without such amplification, no signal was detected for either cell line, while a strong signal was observed following 25 cycles of PCR in the case of the HER2/*neu*-positive cell line.

**Figure 41:**
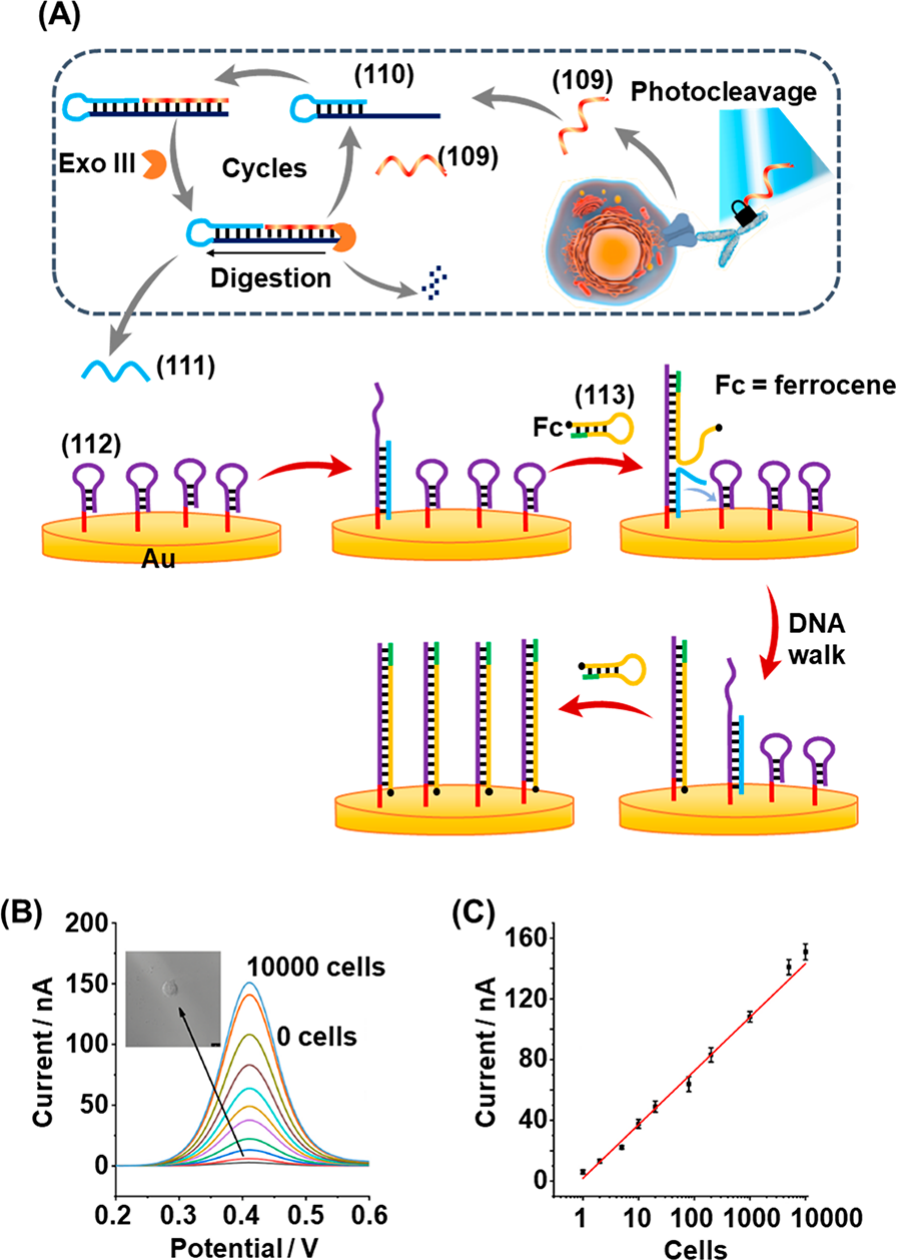
(A) Schematic application of an ONB-bridged antibody-nucleic acid-based conjugate for the detection of the K^+^-ion channel protein in HEK 293 cells using an auxiliary amplification machinery for the DNA label amplification and its electrochemical sensing. The photoresponsive ONB-antibody-nucleic acid barcode conjugate associated with the cells is photocleaved and the resulting cleaved barcode (**109**) is amplified by a probe (**110**)/Exo III machinery, recycling (**109**), and generating strand (**111**) as a “waste” product. The “waste” strand opens the hairpin-monolayer (**112**) functionalized electrode and the resulting toehold-modified monolayer is quantitatively analyzed by a ferrocene reporter unit (**113**). The intensity of the voltammetric response of the functionalized electrode directly relates to the number of cells containing the K^+^-ion channesl. (B) Panel I – Voltammetric responses of the sensing module upon analyzing different number of HEK-293 cells. Panel II – Derived calibration curve. Figure adapted with permission from ref. (406). Copyright 2019, Elsevier.

Another photocleavable DNA/antibody-conjugate strategy, using an alternative signal amplification method, was deployed for quantification of the level of hERG potassium channel expression on the surface of HEK293 cells, Figure 41A.^406^ In this system, the DNA released by photocleavage of the ONB linker (**109**) from the DNA/Ab conjugate was engineered to hybridize with the 3′ single-stranded toehold overhang of DNA duplex (**110**). Within the resulting hybrid the 3′ end termini of (**110**) became reactive toward digestion by the Exonuclease III enzyme, resulting in release and regeneration of (**109**) to participate in further amplification cycles and the release of the single strand (**111**). Strand (**111**) then triggered the opening of a DNA hairpin (**112**), which was immobilized on the surface of a gold electrode. The toehold generated by the opening of hairpin (**112**) triggered the opening of a second DNA hairpin (**113**) labeled with ferrocene (Fc), which displaced the intermediate strand (**111**), which was then available to trigger the opening of another hairpin (**112**) on the electrode surface. Thus, an entropy-driven chain reaction proceeds in which strand (**111**) walks across the electrode surface triggering numerous hybridization reactions with Fc-labeled hairpin (**113**), leading to high coverage of the gold electrode with the redox-labeled strand (**113**). The voltametric response of the Fe-modified electrode was correlated with the number of ion channels associated with variable quantities of HEK 293 cells. Because of the high signal amplification generated by the Exonuclease and entropy-driven chain reaction amplification mechanisms triggered by the photorelease of strand (**109**) from the DNA/antibody conjugate, the sensitivity afforded was sufficient to observe labeled ion channels in a single cell, Figure 41B and Figure 41C.

## 5 Conclusions and Perspectives

The present review article has summarized the roadmap of applications of photoprotected nucleic acid structures, materials, and DNA systems. The base sequence comprising nucleic acids and DNA structures encodes structural and functional properties into the biopolymers and the photoprotection of the nucleic acids provides a versatile means to protect and cage this information in the biopolymer and DNA systems. The photodeprotection of the caging units introduces, then, a general path for the light-induced triggered spatiotemporal uncaging of the nucleic acid structures and the activation of their structural and functional properties and the respective targeted, dictated applications. These include the photoactivation of hairpin caged-protected structures toward catalytic reactions used for gene and RNA detection and imaging, the photodeprotection of caged aptamers, DNAzymes and enzymes, and the spatiotemporal regulation of gene expression and protein synthesis. Also, photodeprotection of caged nucleic acid structures has been used to operate logic gates and computing circuitries. Additional applications employing light-induced deprotection of protected nucleic acid structures included the fusion of biointerfaces, the patterning of hydrogels and microparticles, the light-activated uncaging of drug carriers and biomaterials, and controlled patterning of cells.

While diverse application of photoresponsive caged nucleic acids were demonstrated, the topic is not free of limitations and important challenges are ahead of us. At present, most of the systems made use of the ONB protecting group integrated into the nucleic acid backbone by its incorporation into the biopolymer chain or by modification of the purine or pyrimidine bases of DNA with the ONB protecting group and by the photochemical deprotection of the protecting group by UV light, λ = 365 nm. The use of blue light to deprotect the caging units is certainly a disadvantage, especially for applications in biological environments sensitive to the harmful UV irradiation. Several methods to overcome these limitations were undertaken, including the chemical modification of the ONB moieties with electron donating substituents, red-shifting the absorption of the aryl unit, the application of UCNPs shifting the excitation wavelength to the near-IR region, and the use of two-photon laser excitation. While these approaches provide partial solutions to the photophysical limitations of the ONB groups, the synthetic difficulties and limited chemical means to resolve these difficulties and the need for auxiliary components (UCNPs) and additional instrumental tools suggest that the chemical development of novel, visible-light active protection groups is of urgent importance. In fact, several other photoprotecting groups such as coumarin or quinoline-based dyes were employed to protect bioactive ligands such as ATP.^407^ Not surprisingly, attempts to apply these photoactive constituents to protect nucleic acid structures were reported. For example, the click-chemistry caged aptamer with the photoresponsive coumarin was introduced (c.f. Figure 22)^277^ and the quinoline-based caging of the phosphate constituents of the thrombin aptamer and the photochemical uncaging of the aptamer toward binding of thrombin was reported.^408^ In addition, the coumarin caging of an RNA and its photochemical activation for gene silencing was mentioned in the relevant section (Section 4.3).^271^ Nonetheless, the applications of these photoresponsive protecting groups, as compared to the ONB moiety, are scarce.

In addition, the identification of new applications of photoresponsive protecting groups is important. The major advantage of photoresponsive protecting groups is the spatiotemporal activation of chemical functionalities and processes. Thus, the development of innovative applications of photoresponsive protecting groups rest on their coupling to dynamic chemical systems or their conjugation to microscale or nanoscale structures where spatially resolved activation of chemical reactions is needed. In this context, the review article has emphasized the use of ONB-modified nucleic acids coupled to DNA nanotools, such as nanoelectrodes or supramolecular DNA assemblies, for monitoring intracellular transformations or sensing intracellular agents, such as mRNAs, at the single-cell level. While this area remains challenging and at its infancy, the application of such ONB-modified nanotools for the sensing of other biomarkers, such as aptamer-ligand complexes, or for probing other intracellular transformations, e.g., apoptosis or cell metabolism, may be envisaged.

Substantial recent research efforts are directed toward the development of nucleic acid-based constitutional dynamic networks (CDNs)^286,287,409^ and transient, dissipative reaction modules.^410−412^ The structural reconfiguration of CDNs revealing adaptive,^413^ feedback,^414^ cascaded,^415^ and intercommunicating^416^ properties using different triggering inputs such as fuel strands,^417^ formation and dissociation of G-quadruplexes or triplex structures,^418^ or the applications of *trans*-azobenzene photoisomerizable intercalators^160^ was demonstrated. The use of photoresponsive protected nucleic acid strands could provide versatile means to trigger the dynamic reconfiguration of CDNs. Similarly, recent research efforts are directed to develop transient, out-of-equilibrium systems. Different auxiliary triggers including enzymes,^419−421^ DNAzymes,^398^ and light^159^ were used to fuel these dynamic reaction modules and different applications of the systems were suggested, such as transient aggregation/deaggregation of nanoparticles^422^ or dynamic formation and separation of microfibers were demonstrated.^423^ The use of photoresponsive protected nucleic acid strands could provide caged fuels for the spatiotemporal triggered activation of transient reaction modules.

In addition, extensive research efforts are directed toward the development of gated nucleic acid-based drug carriers. Different drug-loaded nanoparticles or microparticles such as SiO_2_ nanoparticles,^110,114,424,425^ metal–organic framework nanoparticles,^115,426^ or hydrogel microcapsules were caged by nucleic acid gates being unlocked by auxiliary triggers such as pH,^114,115,425^ biomarkers such as ATP,^426^ VEGF^427^ or miRNAs,^428,429^ and enzymes,^430,431^ and their in vitro cytotoxicity were reported. The conjugation of photoresponsive protecting units to the gating units could provide versatile means for the spatiotemporal uncaging of these drug carriers, thereby providing new dimensions for the targeted dynamic therapeutic applications.

Furthermore, DNA nanotechnology provides 2D and 3D nanostructures such as origami scaffolds exhibiting programmable dynamic properties. For example, by appropriate design of two-dimensional origami tiles, the triggered DNAzyme-induced unlocking of origami patches to form programmed nanoholes and guide catalytic transformations in confined nanoenvironments was demonstrated,^432,433^ and by the functionalization of the origami edges with responsive nucleic acid strands, the triggered dimierization of the origami rafts was achieved.^378^ By caging of the functional units of the origami tiles with photoresponsive protecting units, the light-triggered activation of the dynamic functions of the DNA nanostructures can be envisaged.

This review article has emphasized the versatile applications of photoresponsive protected DNA monolayer interfaces or nucleic acid-based materials for the spatial photolithographic patterned deposition of functional nanostructures. Most of the studies made use of photolithography through masks. The development of scanning optical techniques, such as near-field scanning optical microscopy,^434^ could provide, however, further tools for spatial patterning of interfaces.

Finally, the paper has exemplified the use of photoresponsive ONB-protected nucleic acid fluorescent probes or ONB-protected nucleic acid electroactive probes for intracellular sensing applications. These optical and electrochemical tools can be adapted for the spatiotemporal probing of many other chemical and metabolic transformations at the single-cell level.

To conclude, photosensitive protected nucleic acids have a bright future for broad scientific applications in DNA nanotechnology and materials science.
